# Ring‐Expanded N‐Heterocyclic Carbenes for Copper‐Mediated Azide–Alkyne Click Cycloaddition Reactions

**DOI:** 10.1002/cctc.201701992

**Published:** 2018-03-07

**Authors:** Filip Sebest, Jay J. Dunsford, Matthew Adams, Jeremy Pivot, Paul D. Newman, Silvia Díez‐González

**Affiliations:** ^1^ Department of Chemistry Imperial College London Exhibition Road, South Kensington London SW7 2AZ UK; ^2^ School of Chemistry Cardiff University Cardiff CF10 3AT UK

**Keywords:** alkynes, azides, click chemistry, copper, N-heterocyclic carbenes

## Abstract

A series of well‐defined copper(I) complexes bearing ring‐expanded N‐heterocyclic carbene (NHC) ligands has been applied to the azide–alkyne cycloaddition reaction. The obtained results notably showed that the six‐membered NHC ligands outperform well‐established five‐membered ones. [CuI(Mes‐6)] displayed a remarkable catalytic activity while respecting the strict criteria for click reactions.

## Introduction

The development of copper(I) catalysts for the regioselective cycloaddition of azides and alkynes is one of the latest success stories of organometallic catalysis, and it exemplifies the concept of click chemistry.^1^ Even if L'Abbé had already reported a copper(I)‐catalysed [3+2] cycloaddition reaction in 1984,^2^ the full potential of this reactivity was overlooked until 2002, when Sharpless^3^ and Meldal^4^ reported independently that copper(I) species mediated the cycloaddition of azides and alkynes to yield 1,4‐disubstituted‐1,2,3‐triazoles as single products.^5^

Ligandless systems, and aqueous CuSO_4_/sodium l‐ascorbate in particular, have proven suitable for the preparation of many triazoles; however, the use of ligands in this reaction can stabilise the copper(I) centres, increase their catalytic activity, and even modulate it.^6^ Furthermore, ligands have been instrumental for the mechanistic understanding of this transformation. Polytriazoles represent one of the first family of ligands developed specifically for this cycloaddition reaction,^7^ and extensive kinetic studies revealed that the choice of optimal ligand depends on the actual concentration, pH, and coordinating ability of the solvent.^8^

Considering the relatively low configurational stability of these ligands, the actual active species and the rate‐determining step in this reaction might differ depending on the reaction conditions and the employed copper source/ligand combination.^9^ Hence, it is not overly surprising that strongly coordinating ligands, and N‐heterocyclic carbenes (NHCs) in particular,^10^ have played a major role not only in the development of highly performing catalytic systems, but also in important mechanistic studies for this transformation.^11^

**Figure 1:**
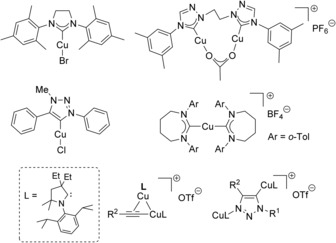
Selected [Cu(NHC)] catalysts for the azide–alkyne cycloaddition and isolated intermediates.

Since the first application of a [Cu(NHC)] to the cycloaddition of azides and alkynes in 2006,^12^ numerous NHC‐based catalysts (including supported ones)^13^ have been disclosed with diverse scaffolds and substituents in a quest for improved catalytic performance (Figure 1).^14^ While C2‐bound NHCs derived from imidazol(in)e remain the most popular motifs, copper complexes bearing non‐classical NHCs have also displayed very good catalytic activities.^15^

Significantly, when using a cyclic (alkyl)(amino) carbene (CAAC) two generally accepted intermediates in this reaction could be isolated and fully characterised: a dinuclear copper acetylide and a bis(copper)triazolide (Figure 1).^16^ It is important to note that kinetic studies showed that both mono‐ and dimeric pathways are active in this case, with the latter being strongly favoured. Unfortunately, the scope and efficiency of [Cu(CAAC)] complexes have not been explored yet. Similarly, to the best of our knowledge, to date there is only one report on the activity of ring‐expanded NHC (^RE^NHC) ligands to this cycloaddition reaction.^17^ [Cu(*o*‐Tol‐7)_2_]BF_4_ was found to be the best catalyst of the series in the absence of solvent (0.5 mol % [Cu]). However, only 2 out of 10 cycloadditions gave conversions above 10 % under the reported conditions.^18^

Still, ^RE^NHCs are known to improve the catalytic efficiency in important transformations such as cross‐coupling,^19^ or allylic boronation^20^ reactions, as well as allowing the study of elusive species (that is, three‐coordinated nickel(I) complexes,^21^ or copper(I)–hydrides^22^). Herein we report the remarkable catalytic activity of several [CuX(^RE^NHC)] complexes in the formation of triazoles under click‐suitable conditions.

## Results and Discussion

### Catalyst preparation and characterisation

**Scheme 1:**
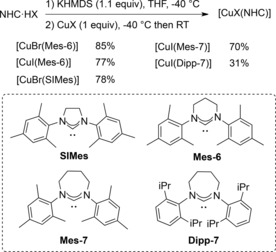
Synthesis of [CuX(NHC)] complexes.

The novel copper catalysts were prepared by the addition of the appropriate CuX salt to the NHC ligand formed in situ by prior deprotonation of the required NHC⋅HX in THF solution (Scheme 1). Previous reports relied on transmetallation reactions from the corresponding silver complexes to prepare related complexes,^23^ but the approach adopted herein has the advantage of yielding the desired complexes in a single step and avoiding the formation of stoichiometric amounts of silver waste. For comparison purposes, benchmark [CuBr(SIMes)]^12^ was also prepared in a similar manner.

The desired complexes were isolated in good yields as cream coloured solids after work‐up and characterised by NMR, HRMS, and elemental analysis. All spectroscopic data showed that the prepared complexes are monomeric in solution at detectable levels, with the notable exception of [CuBr(Mes‐6)]. In this particular case, the isolated product contained 20 % of [Cu(Mes‐6)_2_]^+^ in solution, according to the ^1^H NMR spectrum.^24^ The counterion for this complex would presumably be CuBr_2_^−^.^25^, ^26^ Such a ligand redistribution process led to the formation of an homoleptic complex as the only reaction product in a similar synthesis of a chloro derivative,^25^ but it could not be detected for the related iodo analogue. The reasons for this are not clear, but we had already observed improved bridging abilities for copper(I)–bromo complexes compared to iodo ones with triphosphorous macrocycles.^27^ It is conceivable that μ‐Br species might facilitate the formation of bis‐NHC complexes in this context.

### Catalytic studies

With a series of [CuX(NHC)] complexes in hand, we next tested them on a model reaction under identical reaction conditions. A first set of experiments for the cyclisation of benzyl azide and phenylacetylene with 0.5 mol % [Cu] led to complete conversion with several of the tested catalysts, and in consequence, the metal loading was then lowered to 0.05 mol % to differentiate them (Table 1).

**Table 1:** Catalyst screening.

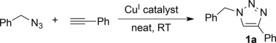				
Entry	Catalyst	[Cu] [mol %]	*t* [h]	Conv [%]^[a]^
1	[CuBr(SIMes)]	0.5	1	>95
		0.05	2	8
		0.05	24	9
				
2	[CuBr(Mes‐6)]	0.5	1	>95
		0.05	2	8
		0.05	4	17
		0.05	8	36
		0.05	24	>95
				
3	[CuI(Mes‐6)]	0.5	1	>95
		0.05	2	>95
				
4	[CuI(Mes‐7)]	0.5	1	<5
		0.5	4	36
		0.5	24	>95
				
5	[CuI(Dipp‐7)]	1.0	24	NR

[a] ^1^H NMR conversions are the average of at least two independent experiments. NR=No reaction.

Overall, [CuI(Mes‐6)] displayed the best catalytic performance and **1 a** was formed quantitatively in 2 h with only 0.05 mol % metal loading (Table 1, entry 3). In these reactions, it was clearly observed that with [CuX(NHC)] complexes, the activity could be ordered as NHC‐6>NHC‐5≫NHC‐7. It is important to note that no catalyst decomposition was observed in these reactions, particularly with [CuI(Dipp‐7)]. The disappointing catalytic activity of copper complexes bearing seven‐membered NHC ligands is uncharacteristic of this family of ligands. Indeed, this trend is the reverse of that observed in other catalytic reactions such as palladium‐mediated C−C cross‐couplings.19b, 28 This might be due to the greater steric hindrance of NHC‐7 ligands when compared to their smaller ring analogues,^29^ but general correlations in catalysis are hard to define owing to the complexity of the mechanisms involved and insufficient comparative data reported to date.

Results in Table 1 confirmed the previously established trend where [CuI(NHC)]>[CuBr(NHC)]>[CuCl(NHC)].^30^ Nevertheless, in this particular case, not only the halogen, but also the speciation in solution needs to be taken into account. Hence, it appears that the specific behaviour of [CuBr(Mes‐6)] in solution is linked to its diminished catalytic activity and therefore, [CuX(^RE^NHC)] are privileged complexes for this cycloaddition reaction when compared to [Cu(^RE^NHC)_2_]X complexes. As previously mentioned,^17^ related [Cu(*o*‐Tol‐7)_2_]BF_4_ only displayed poor catalytic activity in azide–alkyne cycloadditions and we hypothesised this might be due to an inefficient activation of the latter under the reaction conditions.

**Scheme 2:**
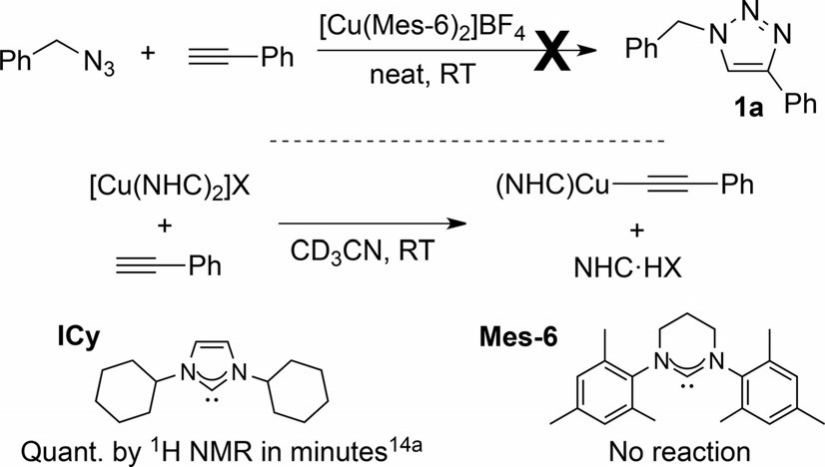
Catalytic tests and proposed activation step for [Cu(NHC)_2_]^+^ complexes.

To be able to draw a direct comparison, [Cu(Mes‐6)_2_]BF_4_ was prepared following a similar procedure to that shown in Scheme 1 and it was then used as catalyst in our model reaction but no triazole formation was observed with either 0.5 or 1 mol % of the homoleptic complex after 24 h (Scheme 2). As we had previously proposed that the first step in the catalytic cycle with homoleptic imidazol‐2‐ylidene complexes involves the displacement of one of the NHC ligand by the alkyne, with the generation of a copper acetylide intermediate,14a we then carried out a similar reaction with [Cu(Mes‐6)_2_]BF_4_, and again no reaction was observed in CD_3_CN, even in the presence of an excess of alkyne (Scheme 2).^24^

While these experiments explain the low catalytic activity of homoleptic copper complexes bearing ring‐expanded NHCs (as well as heteroleptic ones that might rearrange into homoleptic complexes, such as [CuBr(Mes‐6)]), the reasons behind this lack of reactivity are not obvious owing to the lack of data. While tetrahydropyrimidinyl‐2‐ylidenes have been found to be more basic than analogous imidazol‐ or imidazolin‐2‐ylidenes,^31^ and are better electron donor ligands,^32^ little is known about relative dissociation energies and strength of the newly formed NHC−H bonds.

In the light of these results, we also reassessed the activity of [CuBr(Mes‐6)] in azide–alkyne cycloadditions. The model reaction was carried out with 0.05 mol % of [CuBr(Mes‐6)]. In this case, the metal loading was calculated taking into account the presence of inactive [Cu(Mes‐6)_2_][CuBr_2_] and it was assumed that the copper complexes did not interconvert under catalytic conditions. Higher conversions into triazole **1 a** were then obtained, as expected.^33^ However, this complex still failed to match the performance of the iodo analogue.

**Scheme 3:**
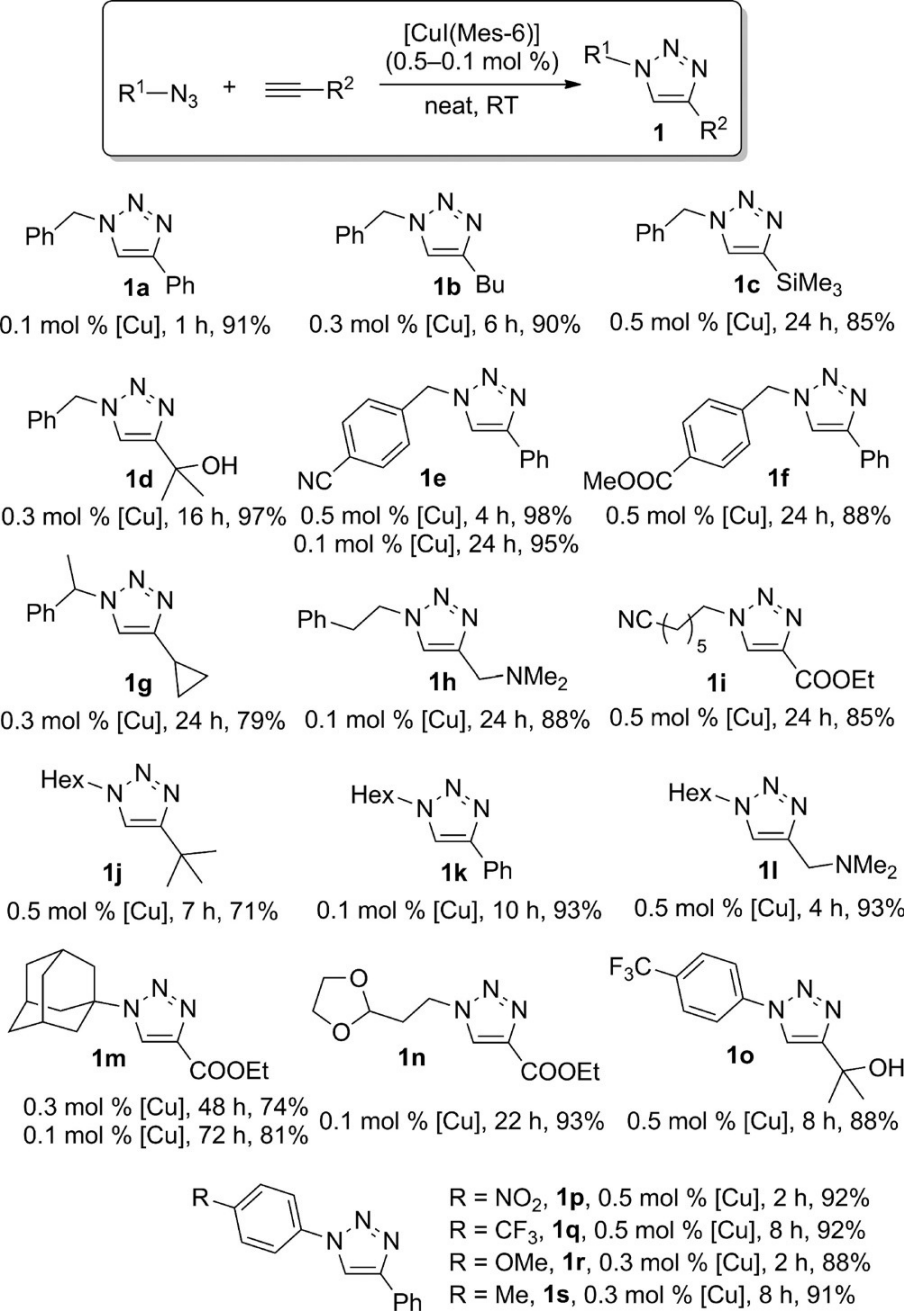
[CuI(Mes‐6)]‐catalysed azide–alkyne cycloaddition reaction.

Having established [CuI(Mes‐6)] as the catalyst of choice, we next explored the scope of the reaction (Scheme 3). Isolated yields in these reactions ranged from 71 to 98 % with most triazoles obtained in 85 % yield or higher. Benzyl, alkyl, and aryl azides were successfully employed under the optimised conditions. Also, a variety of functional groups (such as alcohol, nitrile, amine, silyl, nitro) were tolerated by the catalytic system. Sterically hindered substrates could also be employed, even if a substantially lower reaction rate was observed when adamantyl azide was used as a cycloaddition partner (**1 m** in Scheme 3). Nevertheless, these reactions also show how robust the complex is, since [CuI(Mes‐6)] remained an active catalyst even after three days.

This observation led us to test some of the reactions shown in Scheme 3 with lower metal loadings. The obtained results are summarised in Table 2. Gratifyingly, all four azides tested led to the corresponding triazoles in good to excellent conversions with only 500 ppm of catalyst (100 ppm in the case of **1 a**) and a TON ranging from 1220 and 10^4^. These experiments were not limited to activated substrates and triazoles with bulky substituents (that is, **1 j**) or bearing an additional functional group (for example, amino group on **1 h**) could be prepared under these conditions. In some reactions, though, the catalytic activity eventually seemed to level off, which might be due to the presence of a coordinating group in the substrates (**1 h**) or a significant steric hindrance (**1 j**).

**Table 2:** Low catalytic loading experiments.

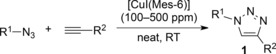					
Triazole	**1**	[Cu] [ppm]	*t* [h]	Conv [%]^[a]^	TON
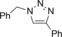	**1 a**	100	24	>95	10 000
					
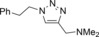	**1 h**	500	24 48 72	57 64 71	1420
					
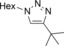	**1 j**	500	24 48	50 61	1220
					
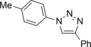	**1 s**	500	24 48 72	30 60 95	1900

[a] ^1^H NMR conversions are the average of at least two independent experiments.

## Conclusions

The screening of different [CuX(NHC)] on a model azide–alkyne cycloaddition reaction has clearly established six‐membered NHC ligands as promising scaffolds for the preparation of 1,4‐disubstituted triazoles under click‐suitable reaction conditions.

These seem to have an optimal stereoelectronic profile for this transformation and a range of triazoles could be efficiently prepared with metal loadings between 0.5 mol % and 100 ppm. We have also shown that the very low catalytic activity of related [Cu(^RE^NHC)_2_]X complexes is most probably due to an inefficient activation step. Further catalytic applications of copper(I) complexes bearing ring‐expanded NHC ligands are currently ongoing in our laboratories and will be reported in due course.

## Experimental Section

Catalytic reactions were carried out in air and using technical‐grade solvents without any particular precautions to exclude moisture or oxygen. The reported isolated yields for the catalytic studies are the average of two independent reactions.

**CAUTION**: Although we did not experience any problems, the cycloaddition of azides and alkynes is highly exothermic and, as a consequence, adequate cooling should always be available when performing these reactions in the absence of solvent.

**Model procedure for click cycloaddition reactions**: In a vial fitted with a screw cap, [CuI(Mes‐6)] (0.5 mol %–100 ppm), azide (0.5 mmol), and alkyne (0.5 mmol) were loaded. The reaction was allowed to proceed at room temperature until full (or no further) conversion was observed by ^1^H NMR spectroscopy. Then, saturated aqueous ammonium chloride solution (10 mL) was added and the resulting mixture was stirred vigorously for 3 h. The resulting precipitate was filtered and washed with water and pentane and then dried under reduced pressure. In all examples, the crude products were estimated to be >95 % pure by ^1^H NMR.

## Conflict of interest

*The authors declare no conflict of interest*.

## Supporting information

As a service to our authors and readers, this journal provides supporting information supplied by the authors. Such materials are peer reviewed and may be re‐organized for online delivery, but are not copy‐edited or typeset. Technical support issues arising from supporting information (other than missing files) should be addressed to the authors.

Supplementary
