# Supplementary material for: Ring‐Expanded N‐Heterocyclic Carbenes for Copper‐Mediated Azide–Alkyne Click Cycloaddition Reactions
Source: ChemCatChem. 2018 Mar 7;10(9):2041–5. doi: 10.1002/cctc.201701992 (PMC5969269; doi:10.1002/cctc.201701992)
Supplement: Supplementary file 1 — Supplementary [file CCTC-10-2041-s001.pdf]

Heterogeneous & Homogeneous & Bio- & Nano-
CHEMCATCHEM
CATALYSIS

Supporting Information

Ring-Expanded N-Heterocyclic Carbenes for Copper-Mediated Azide–Alkyne Click Cycloaddition Reactions

Filip Sebest,[a] Jay J. Dunsford,[b] Matthew Adams,[a] Jeremy Pivot,[a] Paul D. Newman,[b] and Silvia Díez-González*[a]

cctc_201701992_sm_miscellaneous_information.pdf

Table of Contents

1. General Considerations S2
2. Synthesis and Characterisation of [Cu(NHC)] Complexes S2
3. Stoichiometric Reactions S15
4. Synthesis of Azides S16
5. Preparation of [1,2,3]-Triazoles (**1**) S19
6. References S24

NMRs of [1,2,3]-Triazoles (**1**)

1. GENERAL CONSIDERATIONS

All reagents were purchased from commercial sources and used without any further purification. Anhydrous THF was obtained by distillation over sodium/benzophenone under a nitrogen atemosphere. ^{1}H NMR (400 MHz, 500 MHz) and ^{13}C NMR (100 MHz, 125 MHz) spectra were recorded at room temperature unless stated otherwise. Chemical shifts, δ, are reported in ppm with respect to tetramethylsilane (^{1}H NMR), $CDCl_3$ or DMSO-d6 (^{13}C NMR) or monofluorobenzene (^{19}F NMR) as internal standards. Multiplicity is abbreviated to s (singlet), d (doublet), t (triplet), q (quartet), quint (quintet), m (multiplet), and br (broad). IR spectra were recorded with neat samples using a Perkin Elmer Spectrum 100 spectrometer fitted with an ATR accessory. Mass spectra (MS) were recorded on a Micromass Autospec Premier, Micromass LCT Premier or a VG Platform II spectrometer using EI or ESI techniques at the Mass Spectroscopy Service of Imperial College London or using a Waters LCT Premier XE mass spectrometer at Cardiff University. Melting points were determined using an Electrothermal Gallenhamp apparatus fitted with a calibrated thermometer with an error of ±2 °C and are uncorrected. Elemental analyses were performed by London Metropolitan University elemental analysis service.
Catalytic reactions were carried out in air and using technical solvents without any particular precautions to exclude moisture or oxygen. All reported yields are isolated yields and in the catalytic studies are the average of at least two independent experiments.

2. SYNTHESIS AND CHARACTERISATION OF [Cu(NHC)] COMPLEXES

All neutral copper complexes were prepared following the same following procedure: Inside a glove-box, the required NHC·HX salt was suspended in anhydrous THF and cooled to -40 °C whereupon KHMDS (1.1 equiv.) was added as a solid. The mixture was stirred for a further 60 minutes at this temperature before adding the appropriate CuX (1 equiv) as a solid. After stirring for a further 10 minutes at -40 °C the reaction mixture was allowed to reach room temperature and stirred overnight. The resulting mixture was filtered and the volatiles removed under reduced pressure to give a cream solid, which was purified by dissolving it in toluene, filtering through a small Celite plug and removing the solvent *in vacuo*.

[CuBr(SIMes)]

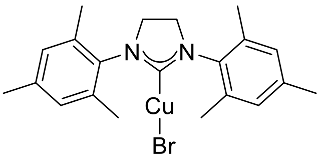

From SIMes·HBr (100 mg, 0.26 mmol) and CuBr (45 mg, 031 mmol), the title compound was isolated in 78% yield (91 mg). Spectroscopic data was in accordance with the literature.[1]
^{1}H NMR (acetone-d6, 400 MHz) δ 7.02 (s, 4H, H^{Ar}), 4.15 (s, 4H, NCH_2), 2.37 (s, 12H, *o*-CH_3), 2.29 (s, 6H, *p*-CH_3); ^{13}C NMR (acetone-d6, 125 MHz) δ 157.2 (NCN), 138.5 (C), 137.0 (C), 135.3 (C), 129.7 (CH), 77.1 (CH_2), 20.9 (CH_3), 18.0 (CH_3).

[CuBr(Mes-6)]

From [Mes-6]HBr (200 mg, 0.50 mmol) and CuBr (86 mg, 0.60 mmol), the title compound was isolated in 85% yield (196 mg).

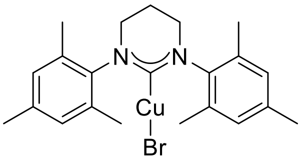

^{1}H NMR (CD_2Cl_2, 400 MHz) δ ([CuBr(Mes-6)]) 6.90 (s, 4H, H^{Ar}), 3.27 (t, J = 5.9 Hz, 4H, NCH_2), 2.25–2.22 (m containing a singlet at 2.23 ppm, 8H, NCH_2CH_2 + p-CH_3), 2.21 (s, 12H, o-CH_3); ^{13}C NMR ($CDCl_3$, 125 MHz) δ 201.3 (NCN), 141.9 (C), 138.0 (C), 134.5 (C), 129.8 (CH), 44.2 (NCH_2), 21.1 (CH_3), 20.8 (CH_2), 18.0 (CH_3); ^{1}H NMR ($CDCl_3$, 400 MHz) δ ([Cu(Mes-6)$_2$][$CuBr_2$]) 6.86 (s, 8H, H^{Ar}), 2.98 (t, J = 5.8 Hz, 8H, NCH_2), 2.27 (s, 12H, p-CH_3), 1.95 (quintet, J = 5.8 Hz, 4H, NCH_2CH_2), 1.67 (s, 24H, o-CH_3); ^{13}C NMR ($CDCl_3$, 125 MHz) δ 198.8 (NCN), 141.5 (C), 138.3 (C), 134.8 (C), 129.8 (CH), 44.3 (NCH_2), 21.2 (CH_3), 20.5 (CH_2), 18.0 (CH_3); HRMS (ES) calculated for $C_{44}H_{56}N_4Cu_2Br$: 845.2280, found: 845.2313 ($[M]_2^+$ – Br); $C_{44}H_{56}N_4Cu$: 703.3801, found: 703.3806 ($[Cu(6\text{-}Mes)_2]^+$); $C_{24}H_{31}N_3Cu$: 424.1814, found: 424.1819 ($[M]^+$ – Br + MeCN). Elemental analysis calc. for $C_{22}H_{28}N_2CuBr$: C, 56.96; H, 6.08; N, 6.04%. Found: C, 56.84; H, 6.17; N, 6.04%

^{1}H NMR spectrum of [CuBr(Mes-6)] in CD_2Cl_2

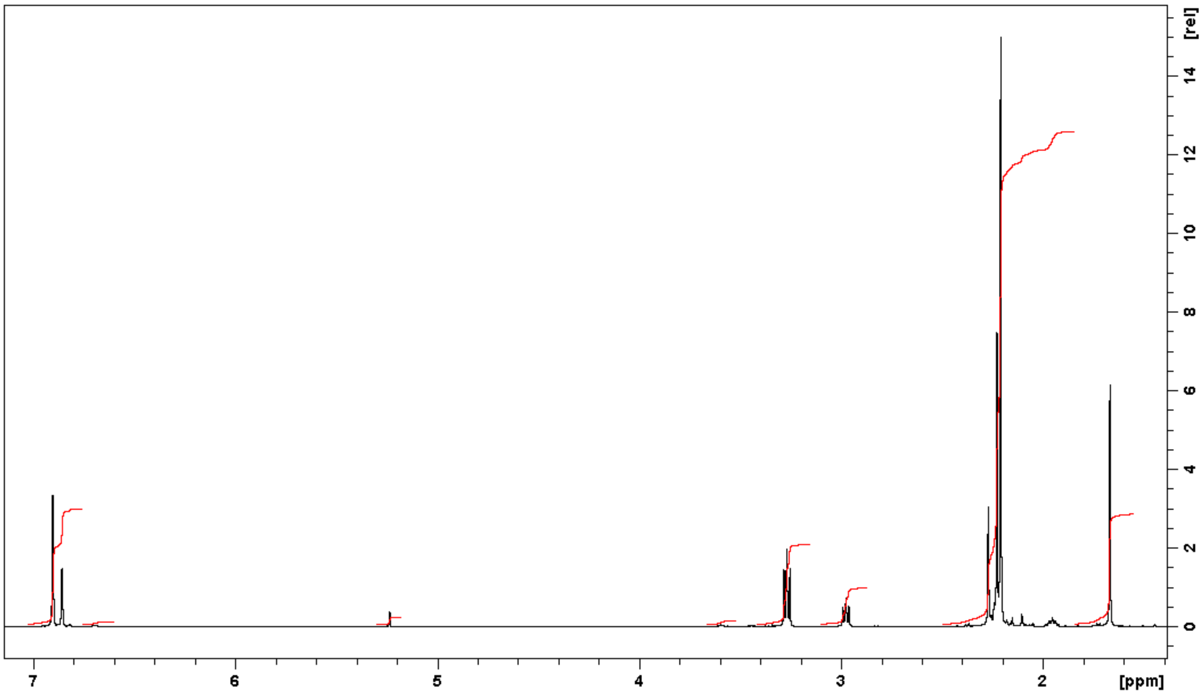

^{1}H-^{1}H COSY NMR spectrum of [CuBr(Mes-6)] in CD_2Cl_2

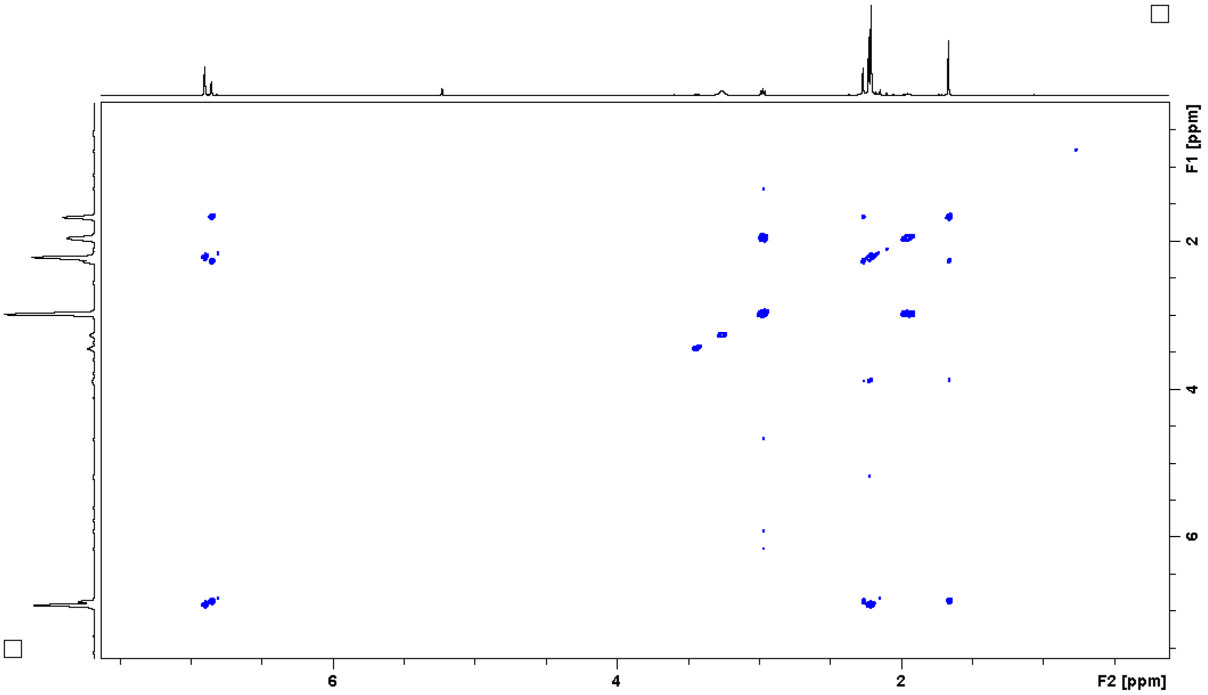

$^{13}C\{^{1}H\}$ spectrum of [CuBr(Mes-6)] in $CDCl_3$

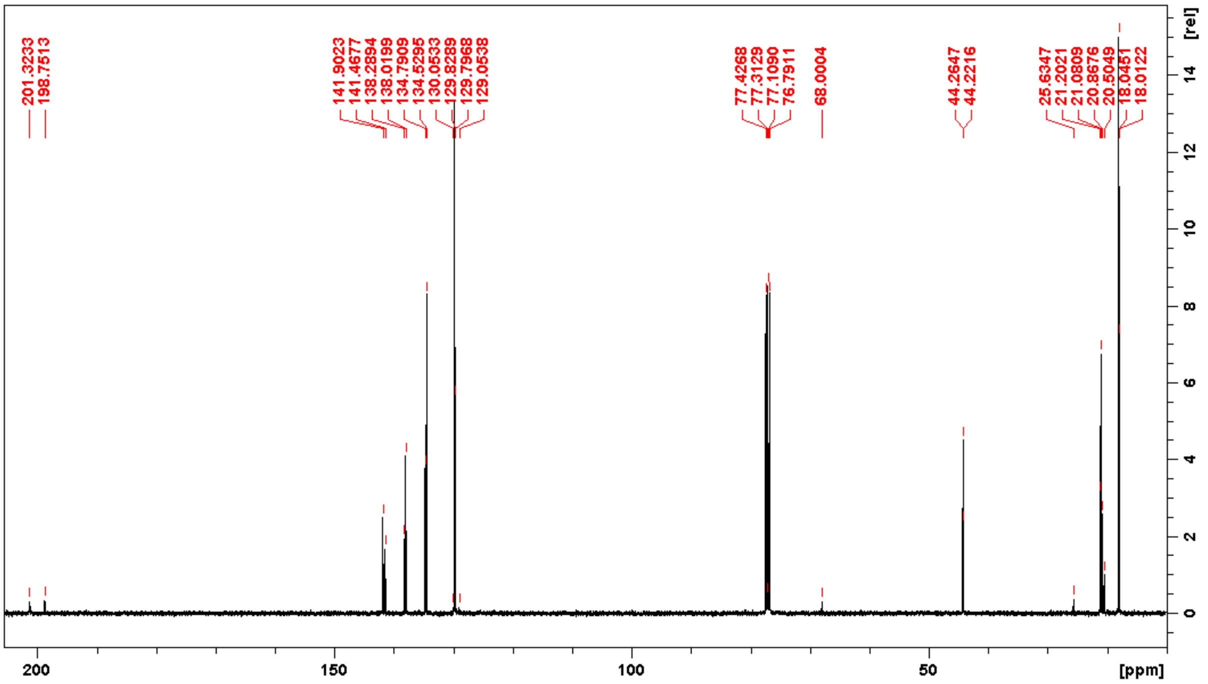

HRMS of [CuBr(Mes-6)]

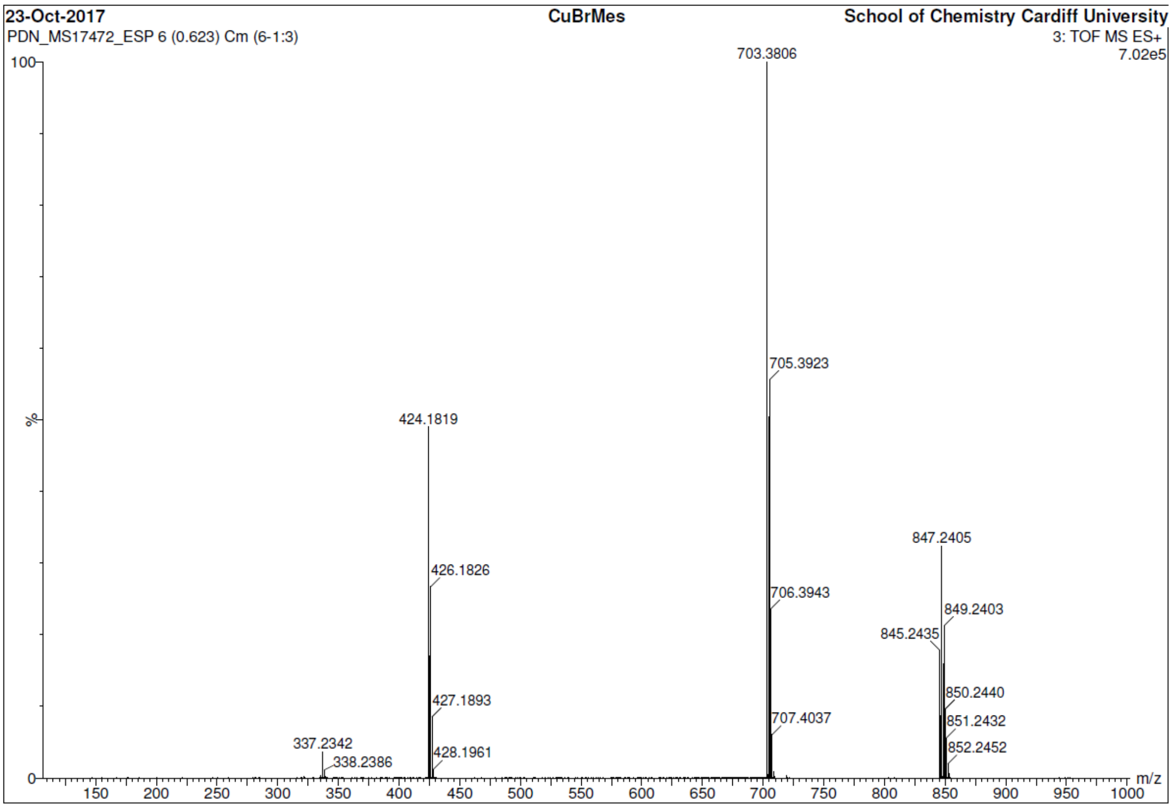

Monoisotopic Mass, Odd and Even Electron Ions
27 formula(e) evaluated with 1 results within limits (up to 50 best isotopic matches for each mass)
Elements Used:
C: 0-44 H: 0-56 N: 0-4 63Cu: 0-2 Br: 0-1

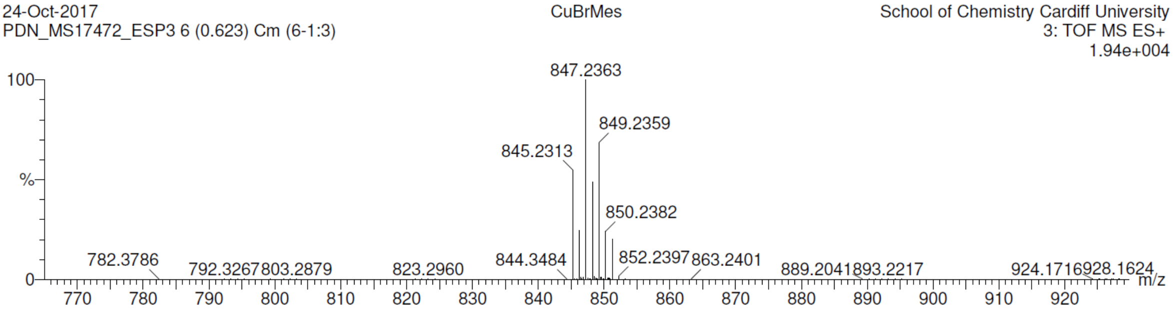

Mass	Calc. Mass	mDa	PPM	DBE	i-FIT	i-FIT (Norm)	Formula
Minimum:				-1.5			
Maximum:		5.0	5.0	100.0			
845.2313	845.2280	3.3	3.9	18.5	254.0	0.0	C44 H56 N4 63Cu2 Br

Monoisotopic Mass, Odd and Even Electron Ions
7 formula(e) evaluated with 1 results within limits (up to 50 best isotopic matches for each mass)
Elements Used:
C: 0-44   H: 0-56   N: 0-4   63Cu: 0-1

23-Oct-2017 CuBrMes School of Chemistry Cardiff University
PDN_MS17472_ESP 6 (0.623) 3: TOF MS ES+
7.02e+005

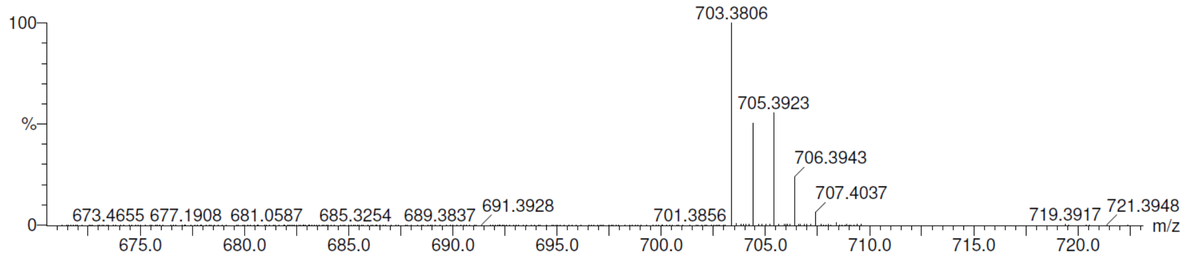

Minimum:				-1.5			
Maximum:		5.0	5.0	100.0			
Mass	Calc. Mass	mDa	PPM	DBE	i-FIT	i-FIT (Norm)	Formula
703.3806	703.3801	0.5	0.7	19.0	505.4	0.0	C44 H56 N4 63Cu

Monoisotopic Mass, Odd and Even Electron Ions
6 formula(e) evaluated with 1 results within limits (up to 50 best isotopic matches for each mass)
Elements Used:
C: 0-24   H: 0-31   N: 0-3   63Cu: 0-1

23-Oct-2017 CuBrMes School of Chemistry Cardiff University
PDN_MS17472_ESP 6 (0.623) Cm (6-1:3) 3: TOF MS ES+
3.44e+005

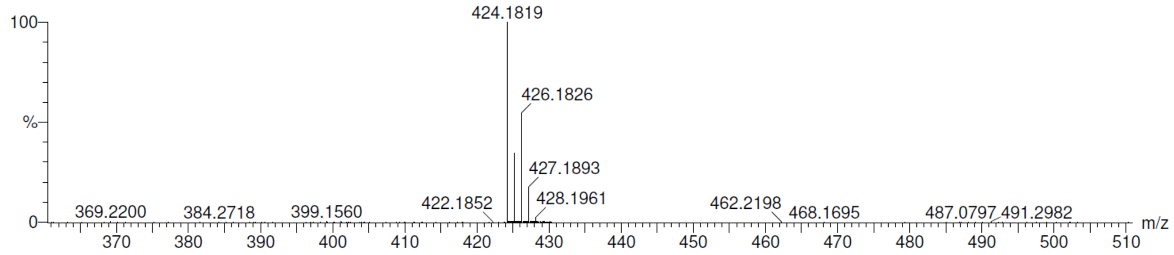

Minimum:				-1.5			
Maximum:		5.0	5.0	100.0			
Mass	Calc. Mass	mDa	PPM	DBE	i-FIT	i-FIT (Norm)	Formula
424.1819	424.1814	0.5	1.2	11.0	831.0	0.0	C24 H31 N3 63Cu

[CuI(Mes-6)]

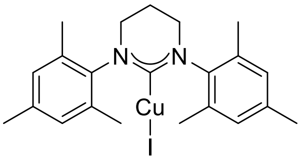

From [Mes-6]HI (200 mg, 0.45 mmol) and CuI (102 mg, 0.53 mmol), the title compound was isolated in 77% yield (227 mg).
^{1}H NMR ($CDCl_3$, 400 MHz) δ 6.87 (s, 4H, H^{Ar}), 3.29 (t, *J* = 5.9 Hz, 4H, NCH_2), 2.27 (quint, *J* = 5.9 Hz, 2H, NCH_2CH_2), 2.21 (s, 18H, *o*-CH_3, *p*-CH_3); ^{13}C NMR ($CDCl_3$, 125 MHz) δ NCN (not observed), 141.6 (C), 138.2 (C), 134.6 (C), 129.8 (CH), 44.3 (NCH_2), 21.1 (CH_3), 20.9 (NCH_2CH_2), 18.1 (CH_3); HRMS (ES) calculated for $C_{24}H_{31}N_3Cu$: 424.1814, found: 424.1819 ($[M]^+$ – I + MeCN). Anal: Calc. for $C_{22}H_{28}N_2CuI$: C, 51.72; H, 5.52; N, 5.48%. Found: C, 51.38; H, 5.56; N, 5.19%

^{1}H NMR spectrum of [CuI(Mes-6)] in $CDCl_3$

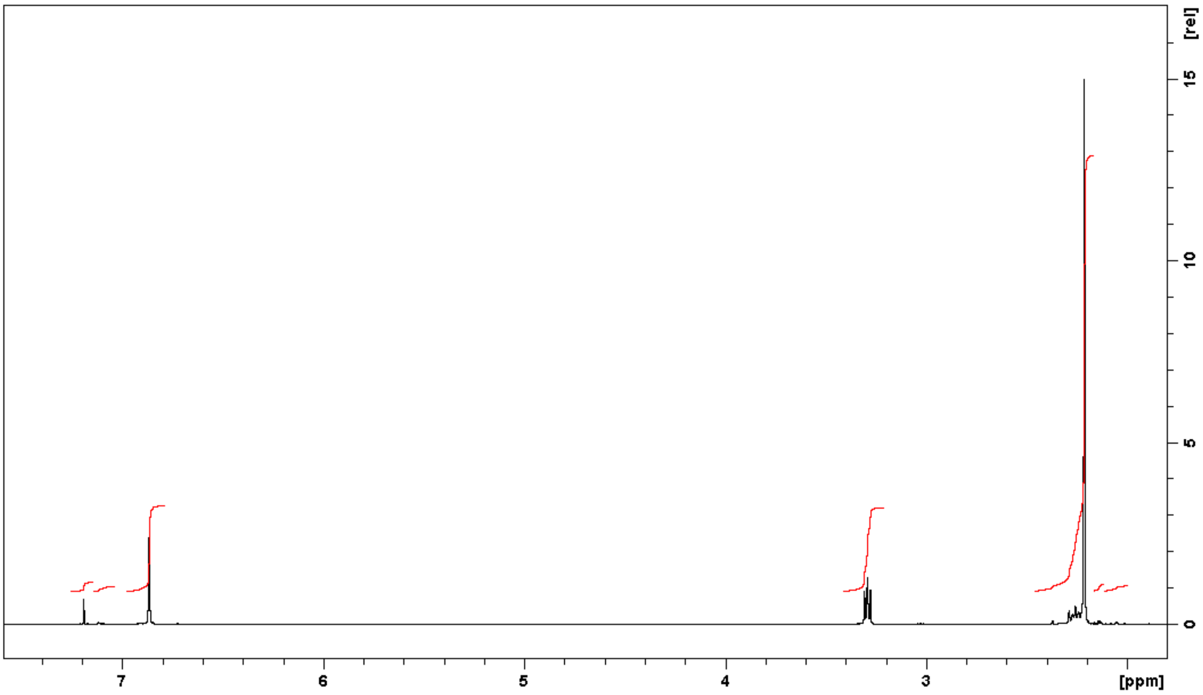

$^{13}C\{^{1}H\}$ NMR spectrum of [CuI(Mes-6)] in $CDCl_3$

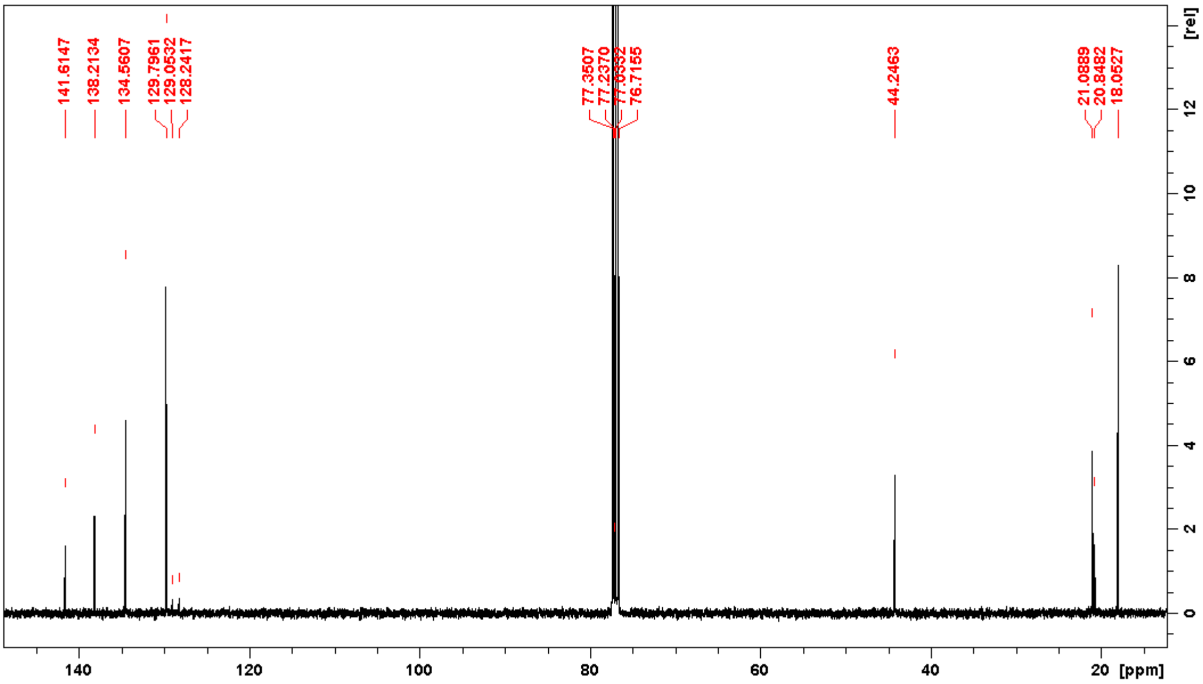

HRMS of [CuI(Mes-6)]

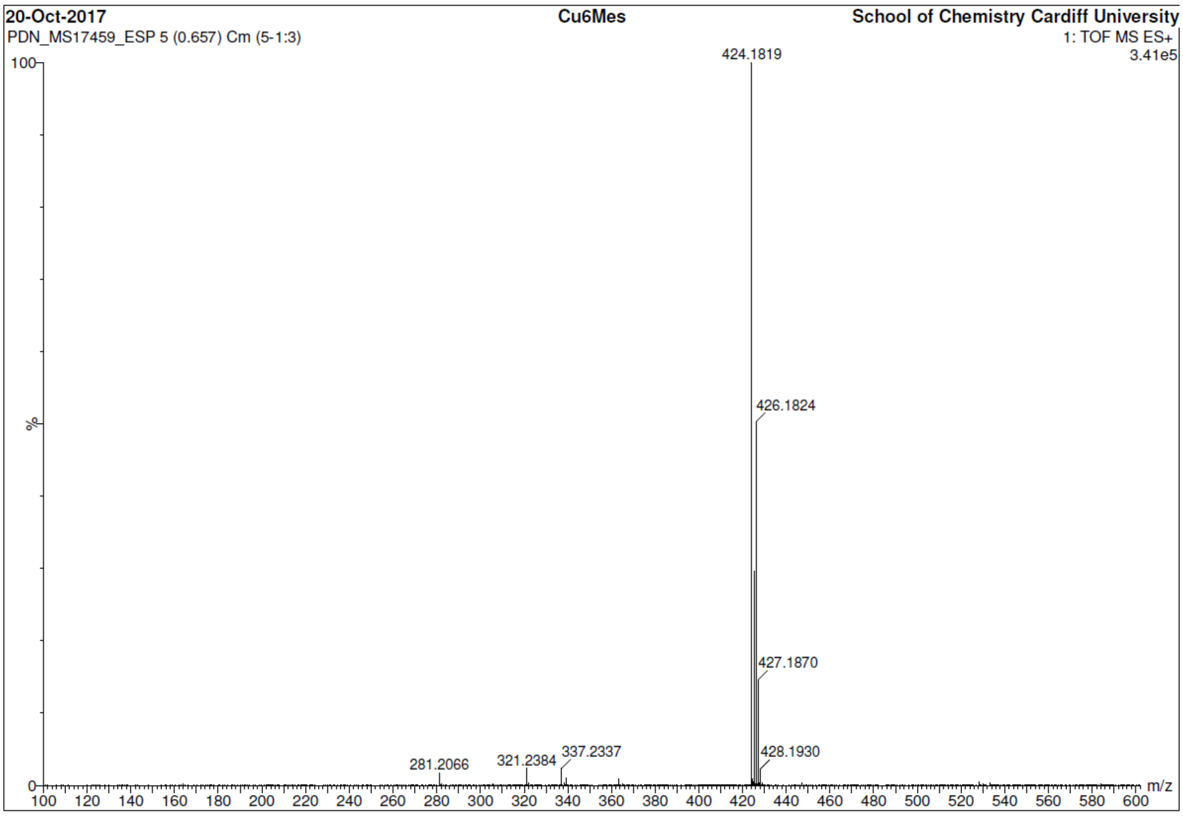

Monoisotopic Mass, Odd and Even Electron Ions
6 formula(e) evaluated with 1 results within limits (up to 50 best isotopic matches for each mass)
Elements Used:
C: 0-24 H: 0-31 N: 0-3 63Cu: 0-1

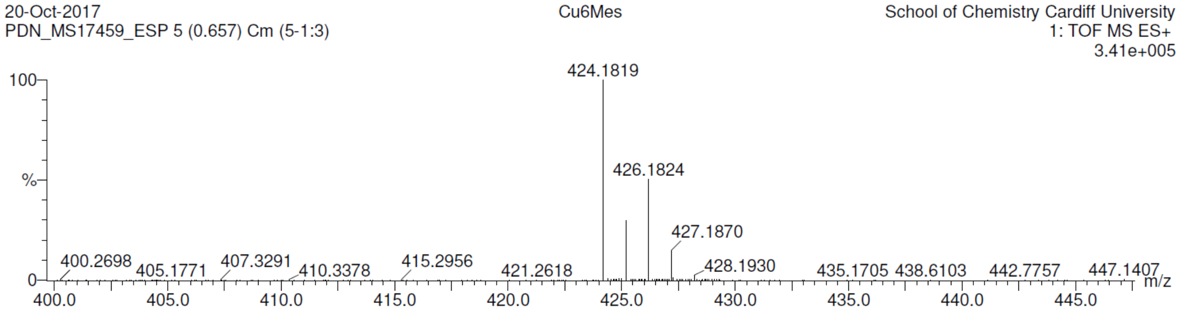

Minimum:				-1.5			
Maximum:		5.0	5.0	100.0			
Mass	Calc. Mass	mDa	PPM	DBE	i-FIT	i-FIT (Norm)	Formula
424.1819	424.1814	0.5	1.2	11.0	730.4	0.0	C24 H31 N3 63Cu

[CuI(Mes-7)]

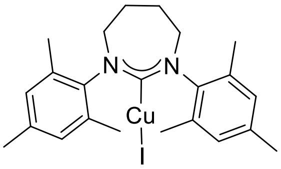

From [Mes-7]HI (100 mg, 0.22 mmol) and CuI (50 mg, 0.26 mmol), the title compound was isolated in 70% yield (113 mg).
^{1}H NMR ($CDCl_3$, 400 MHz) δ 6.86 (s, 4H, H^{Ar}), 3.83–3.78 (m, 4H, NCH_2), 2.28 (s, 12H, *o*-CH_3), 2.26–2.21 (m, 4H, N$CH_2$$CH_2$), 2.20 (s, 6H, *p*-$CH_3$); ^{13}C NMR ($CDCl_3$, 125 MHz): δ NCN (not observed),

144.0 (C), 137.9 (C), 134.2 (C), 129.9 (CH), 52.6 (NCH_2), 25.7 (NCH_2*CH_2*), 21.0 (CH_3), 18.7 (CH_3); HRMS (ES) calculated for $C_{25}H_{33}N_3Cu$: 438.1970, found: 438.1971 ($[M]^+ - I + MeCN$).

^{1}H NMR spectrum of [CuI(Mes-7)] in $CDCl_3$

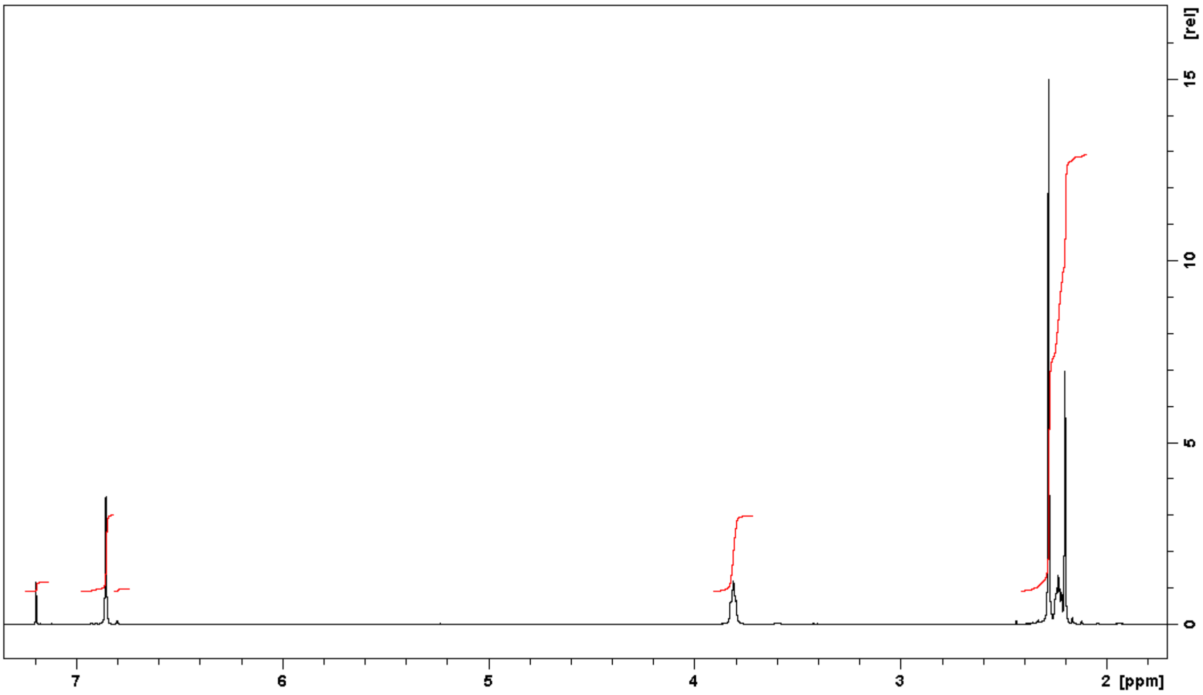

^{13}C{^{1}H} NMR spectrum of [CuI(Mes-7)] in $CDCl_3$

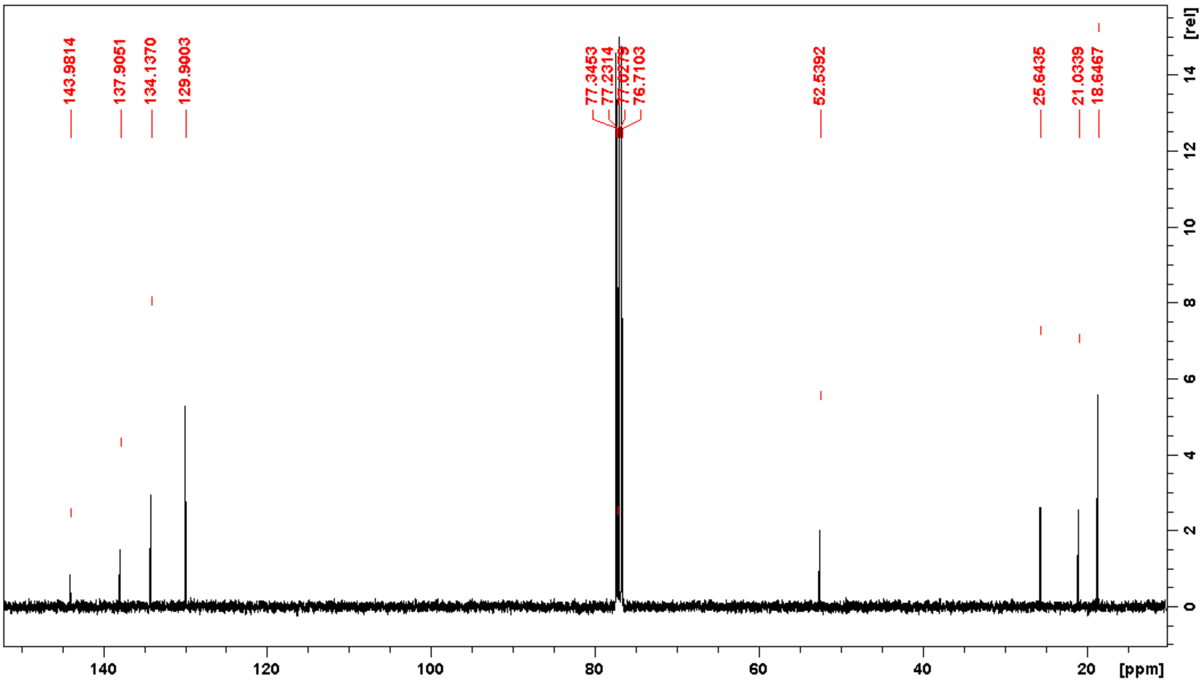

HRMS of [CuI(Mes-7)]

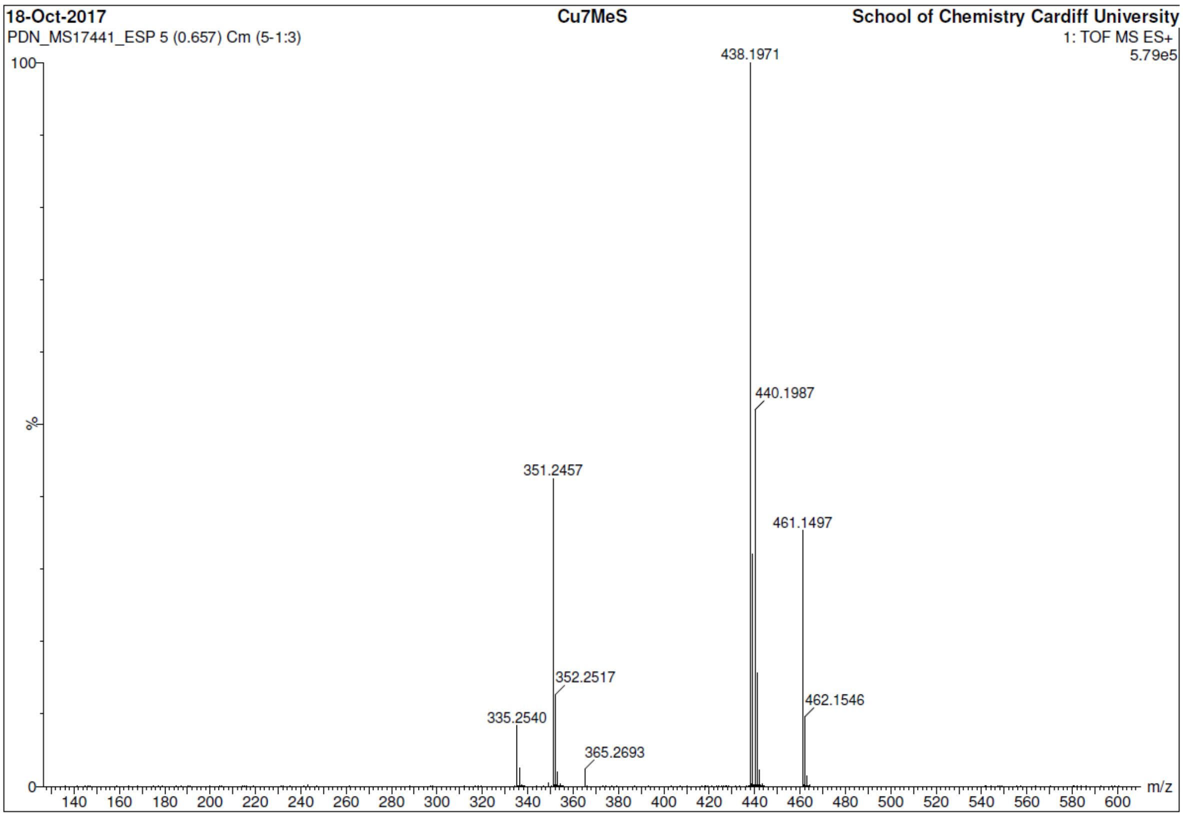

Monoisotopic Mass, Odd and Even Electron Ions
6 formula(e) evaluated with 1 results within limits (up to 50 best isotopic matches for each mass)
Elements Used:
C: 0-25 H: 0-33 N: 0-3 63Cu: 0-1

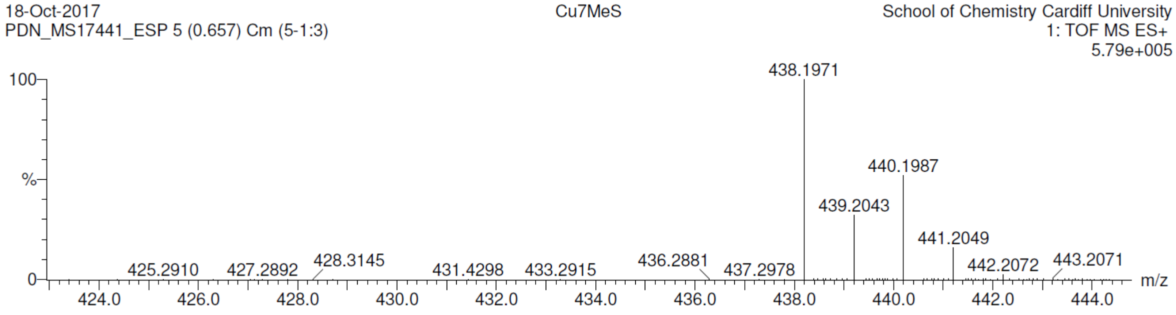

Minimum:				-1.5			
Maximum:		5.0	5.0	100.0			
Mass	Calc. Mass	mDa	PPM	DBE	i-FIT	i-FIT (Norm)	Formula
438.1971	438.1970	0.1	0.2	11.0	780.4	0.0	C25 H33 N3 63Cu

[CuI(Dipp-7)]

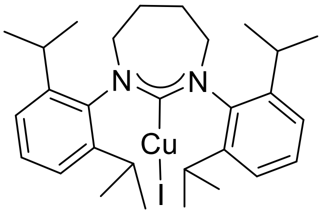

From [Dipp-7]HI (200 mg, 0.37 mmol) and CuI (71 mg, 0.37 mmol), the title compound was isolated in 31% yield (70 mg) after recrystallisation from CH_2Cl_2/pentane.
^{1}H NMR ($CDCl_3$, 400 MHz) δ 7.27 (t, J = 7.7 Hz, 2H, H^{Ar}), 7.13 (d, J = 7.7 Hz, 4H, H^{Ar}), 3.94–3.89 (m, 4H, NCH_2), 3.19 (sept, J = 6.8 Hz, 4H, CH), 2.29 (m, 4H, NCH_2CH_2), 1.30 (d, J = 6.8 Hz, 12H,

CH3), 1.25 (d, *J* = 6.8 Hz, 12H, CH3); ^{13}C NMR ($CDCl_3$, 125 MHz): δ NCN (not observed), 145.0 (C), 143.6 (C), 129.1 (CH), 124.8 (CH), 54.0 (NCH_2), 28.8 (CH), 25.3 (NCH_2CH_2), 24.9 (CH_3), 24.8 (CH_3); HRMS (ES) calculated for $C_{31}H_{45}N_3Cu$: 522.2909, found: 522.2930 ($[M]^+$ – I + MeCN).

<u>^{1}H NMR spectrum of [CuI(Dipp-7)] in $CDCl_3$</u>

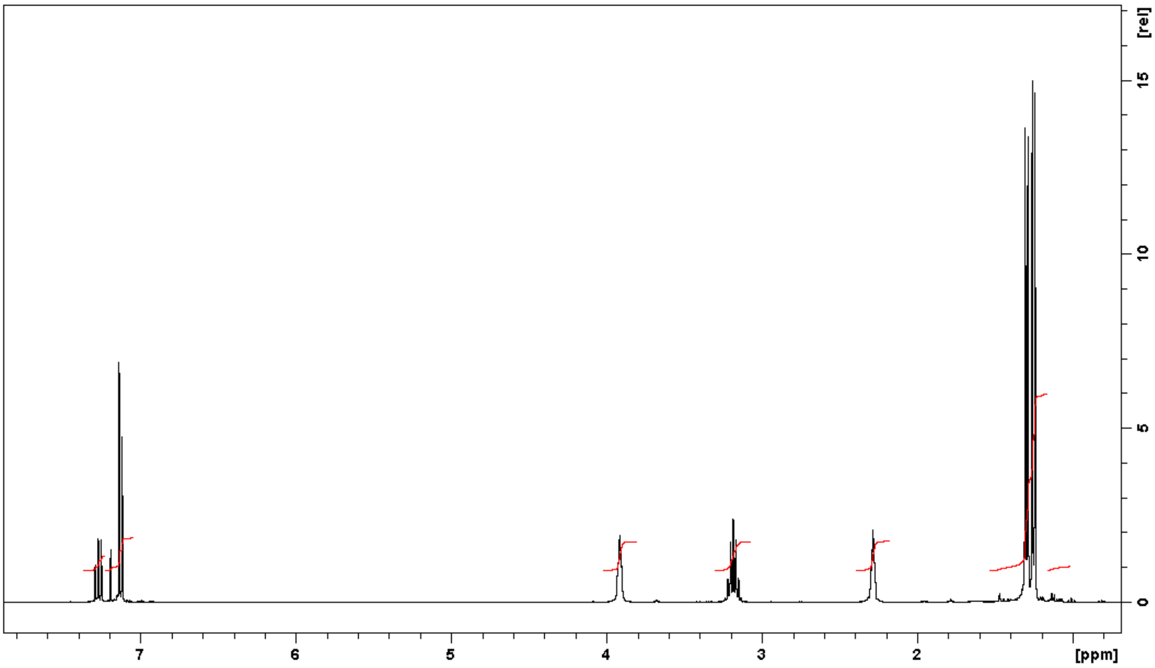

<u>^{13}C{^{1}H} NMR spectrum of [CuI(Dipp-7)] in $CDCl_3$</u>

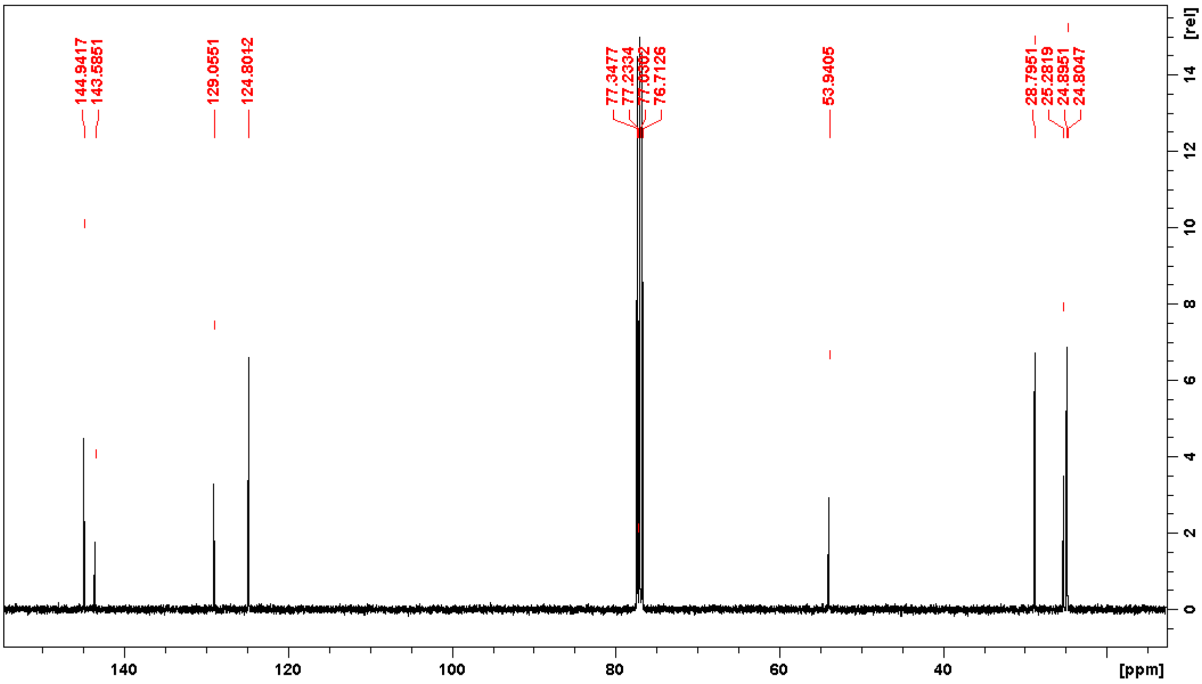

HRMS of [CuI(Dipp-7)]

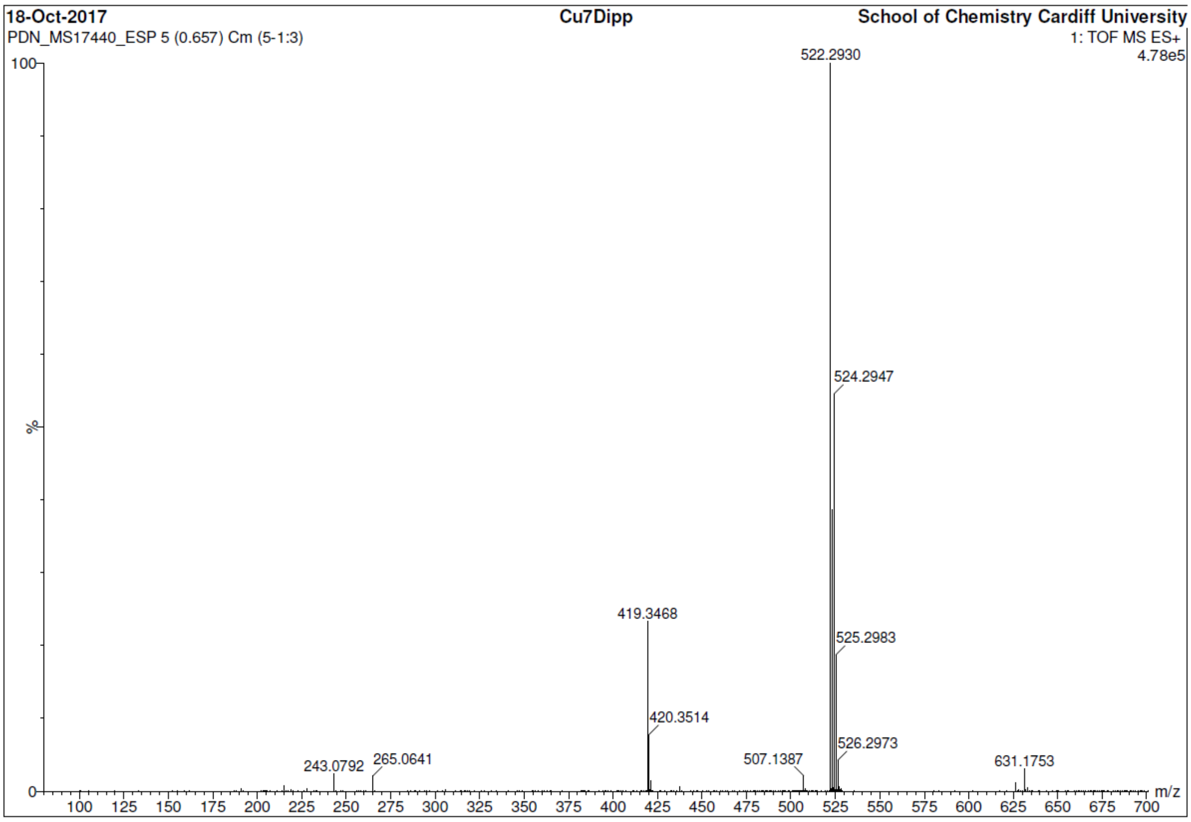

Monoisotopic Mass, Odd and Even Electron Ions
6 formula(e) evaluated with 1 results within limits (up to 50 best isotopic matches for each mass)
Elements Used:
C: 0-31 H: 0-45 N: 0-3 63Cu: 0-1

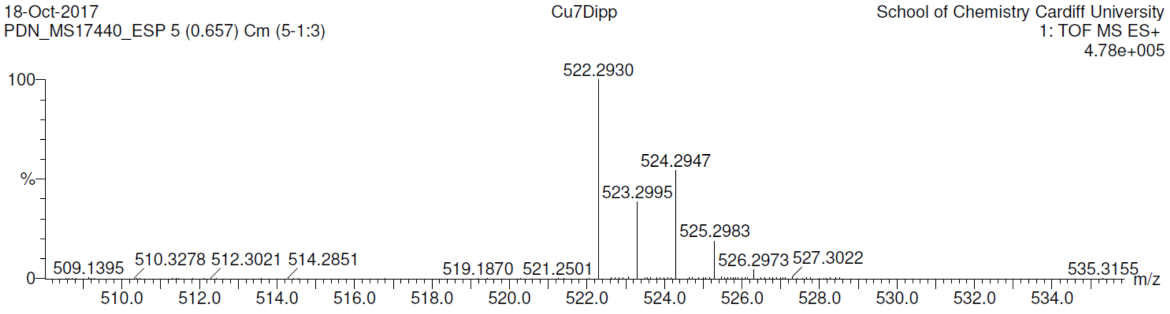

Minimum:				-1.5			
Maximum:		5.0	5.0	100.0			
Mass	Calc. Mass	mDa	PPM	DBE	i-FIT	i-FIT (Norm)	Formula
522.2930	522.2909	2.1	4.0	11.0	692.4	0.0	C31 H45 N3 63Cu

[Cu(Mes-6)$_2$]BF$_4$

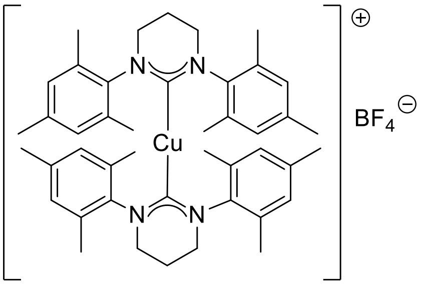

To a solution of [Mes-6]HBF$_4$ (1.0 g, 2.48 mmol) in THF (20 mL) KHMDS (0.53 g, 1.1 equiv) was added and the mixture was stirred for 1 hour. After this time, the mixture was filtered under N_2 onto solid [Cu(MeCN)$_4$]BF$_4$ (0.58 g, 1.84 mmol) and stirred for 24 h. The formed white solid was isolated by filtration, washed sparingly with THF and dried under vacuum. Yield = 0.88 g (90%). Spectroscopic data was in accordance with the literature.[2]

^{1}H NMR (CD$_2$Cl$_2$, 400 MHz) δ 6.85 (s, 8H, H^{Ar}), 2.98 (t, J = 5.8 Hz, 8H, NC*H*$_2$), 2.27 (s, 12H, *p*-CH$_3$), 1.96 (quint, J = 5.8 Hz, 4H, NCH$_2$C*H*$_2$), 1.67 (s, 24H, *o*-CH$_3$); ^{13}C NMR (CD$_2$Cl$_2$, 125 MHz) δ 198.9 (N*C*N), 141.5 (C), 138.2 (C), 134.9 (C), 129.8 (CH), 44.2 (NCH$_2$), 20.8 (CH$_3$), 20.4 (NCH$_2$*C*H$_2$), 17.8 (CH$_3$); HRMS (ES) calculated for C$_{44}$H$_{56}$N$_4$CuBF$_4$: 703.3801, found: 703.3802 ([M]$^+$).

^{1}H NMR spectrum of [Cu(Mes-6)$_2$]BF$_4$ in CD$_2$Cl$_2$

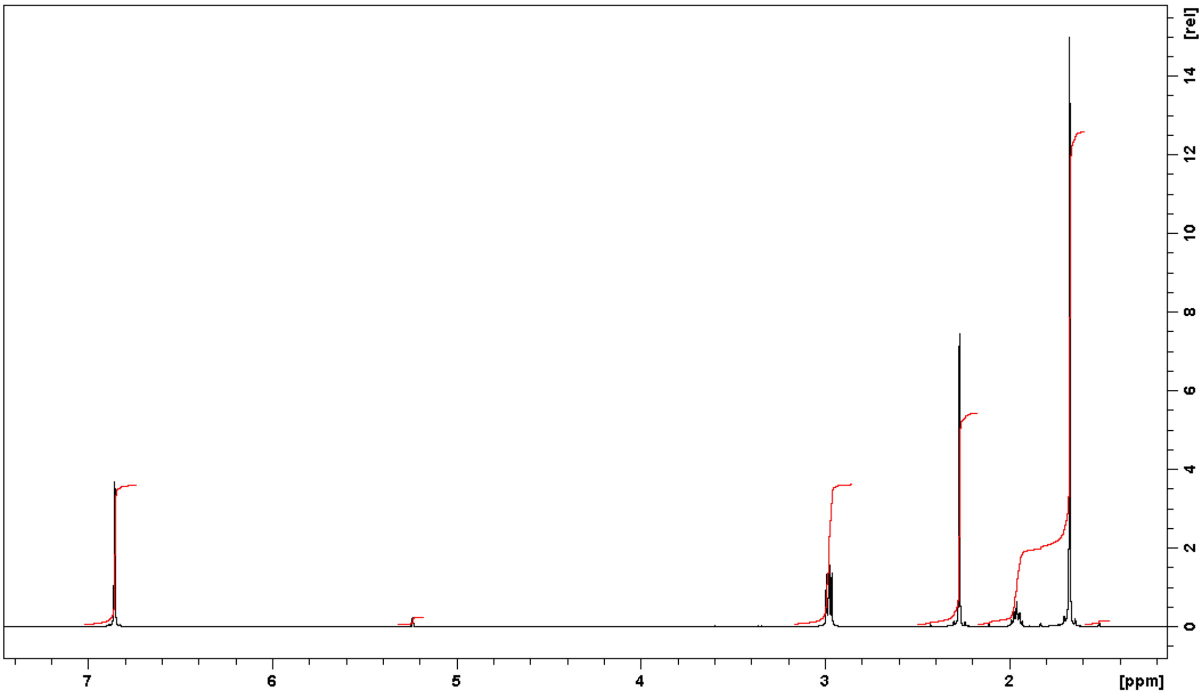

$^{13}C\{^{1}H\}$ NMR spectrum of $[Cu(Mes\text{-}6)_2]BF_4$ in CD_2Cl_2

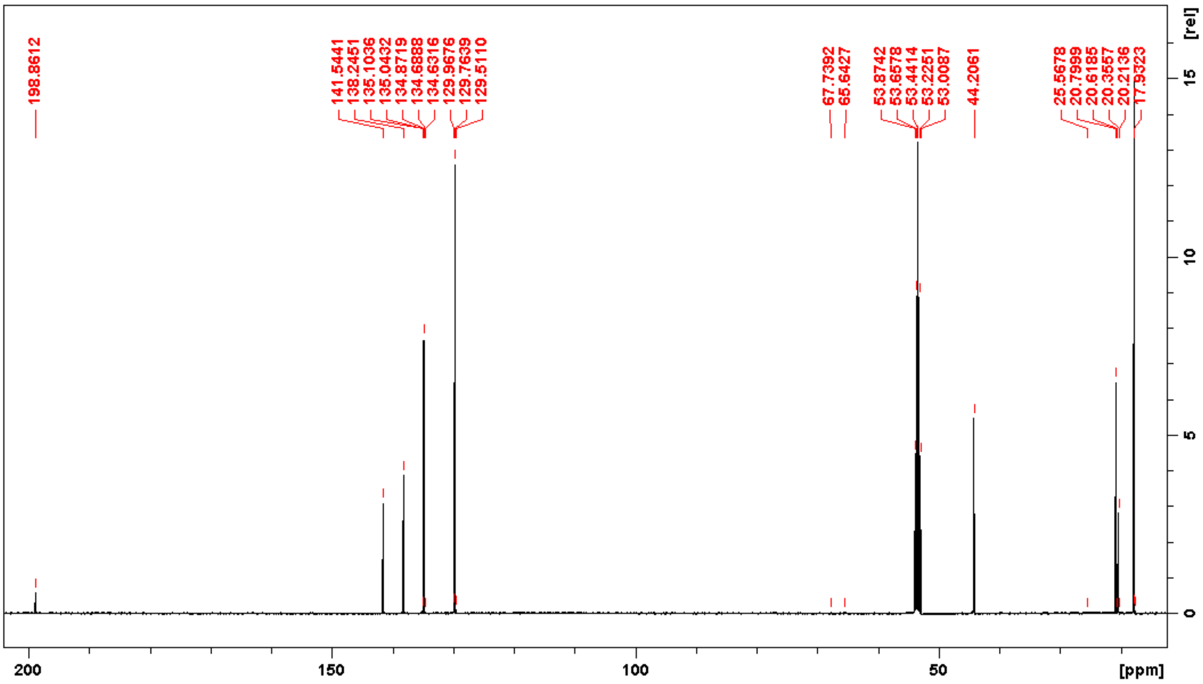

HRMS of $[Cu(Mes\text{-}6)_2]BF_4$

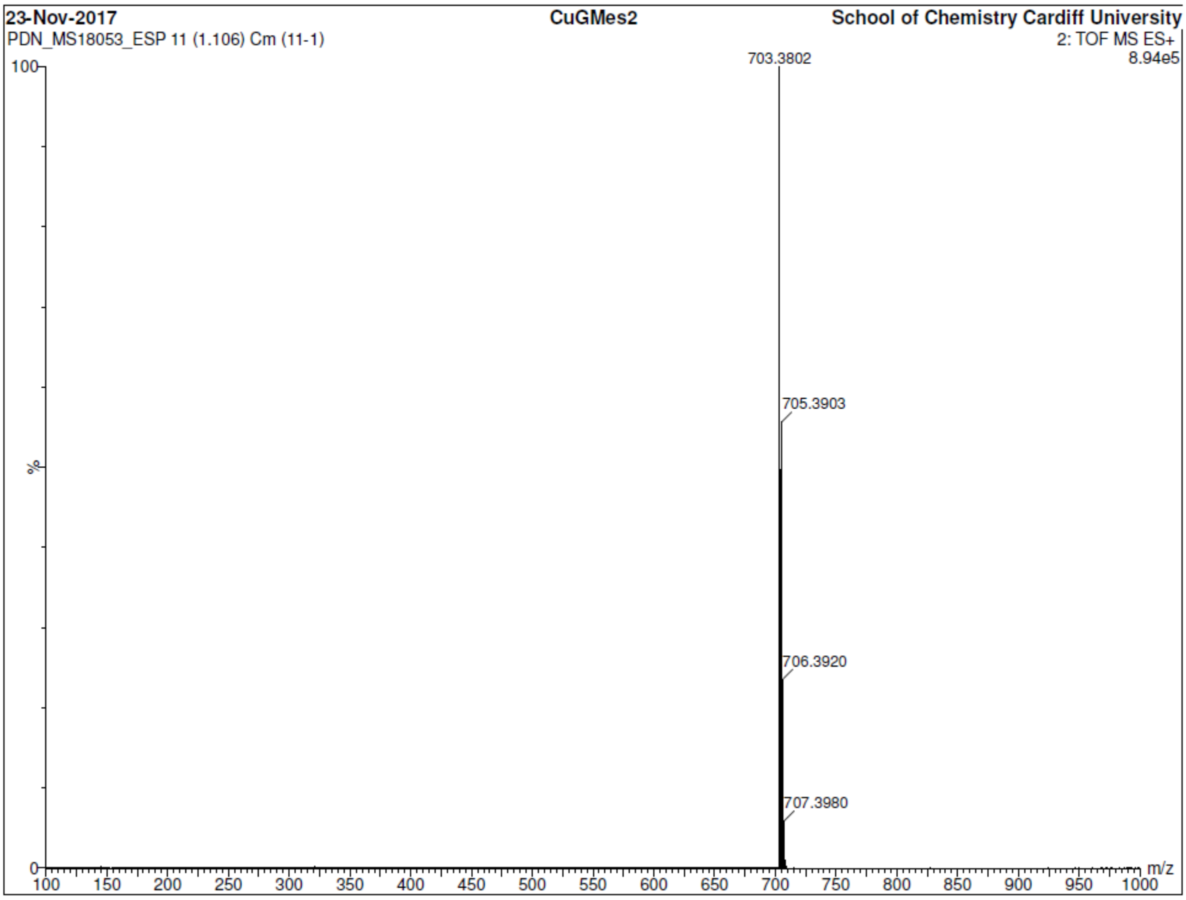

Monoisotopic Mass, Odd and Even Electron Ions
7 formula(e) evaluated with 1 results within limits (up to 50 best isotopic matches for each mass)
Elements Used:
C: 0-44 H: 0-56 N: 0-4 63Cu: 0-1

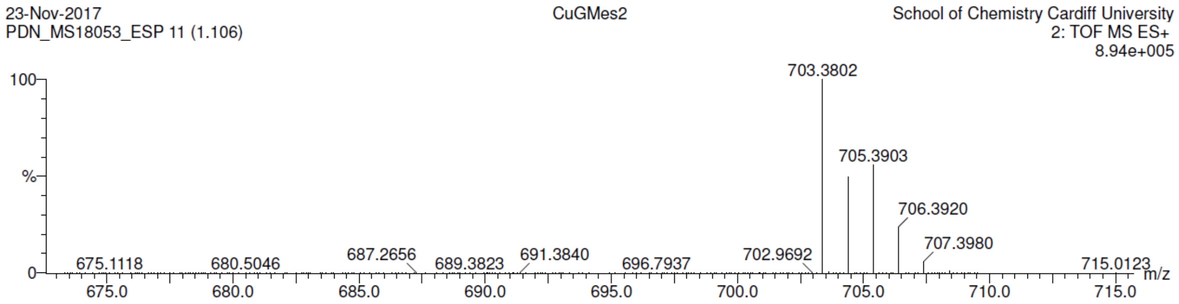

	Calc. Mass	mDa	PPM	DBE	i-FIT	i-FIT (Norm)	Formula
Minimum:				-1.5			
Maximum:		5.0	5.0	100.0			
Mass	Calc. Mass	mDa	PPM	DBE	i-FIT	i-FIT (Norm)	Formula
703.3802	703.3801	0.1	0.1	19.0	553.0	0.0	C44 H56 N4 63Cu

3. STOICHIOMETRIC REACTIONS

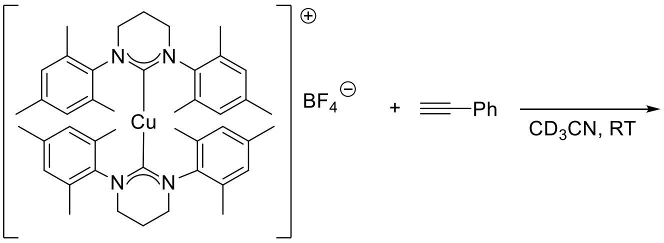

1 equivalent of phenylacetylene:

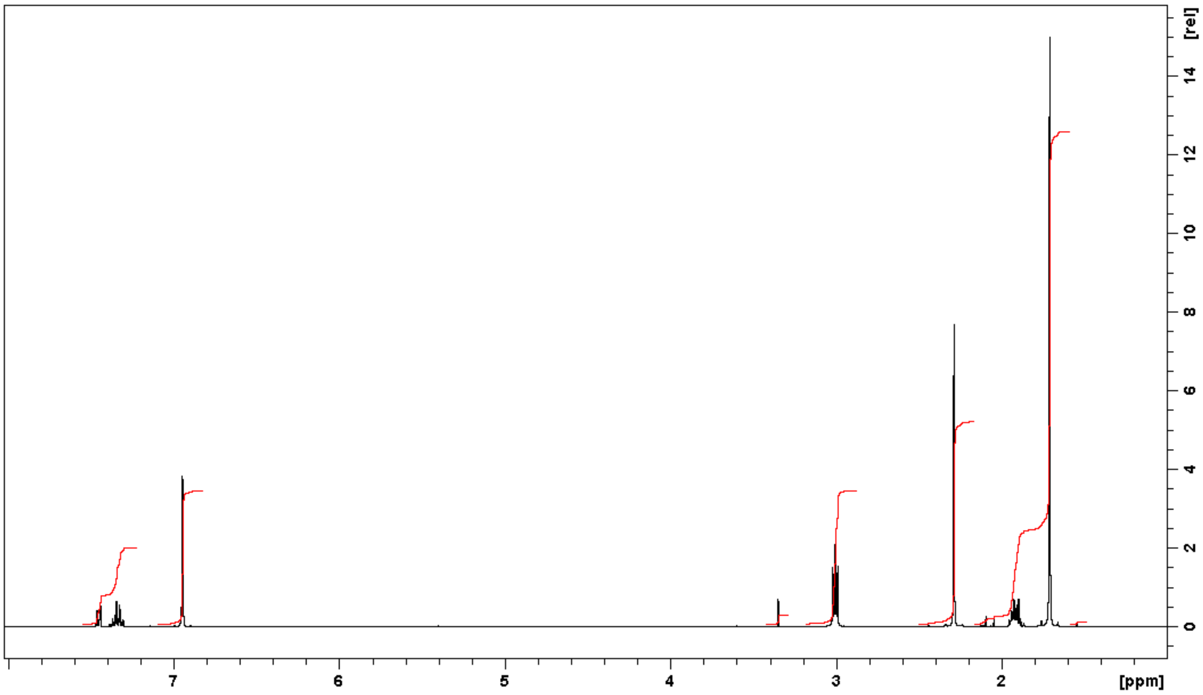

2 equivalents of phenylacetylene:

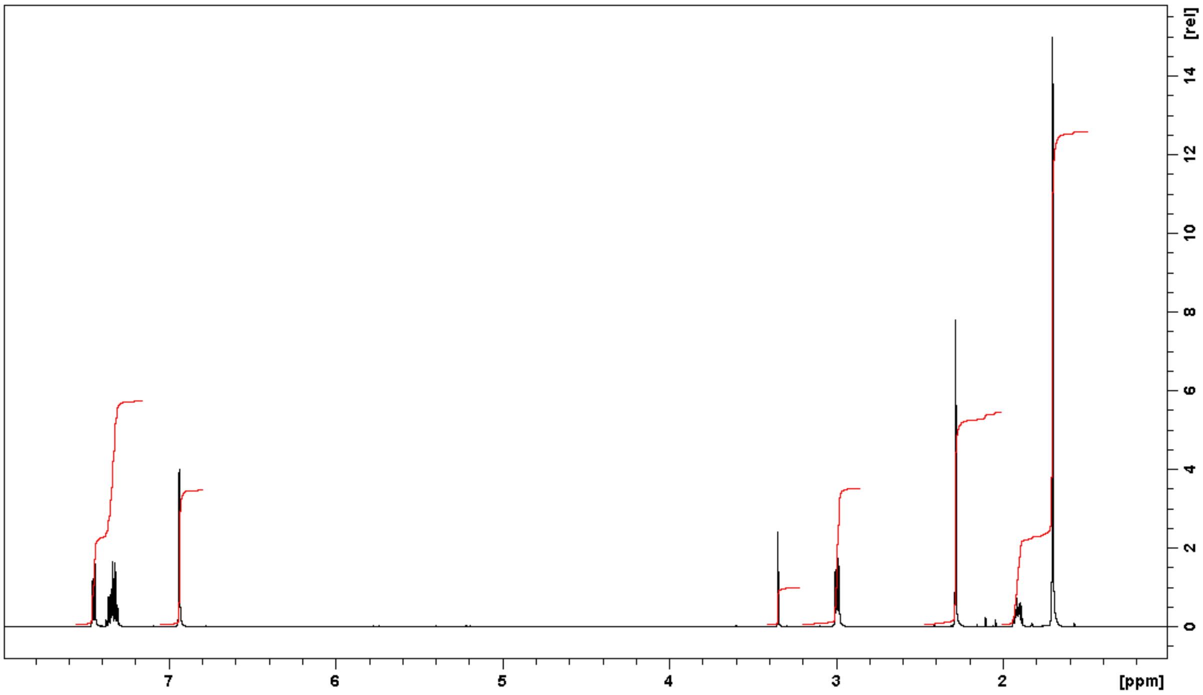

4. SYNTHESIS OF AZIDES

Azides were synthesised at room temperature from the corresponding bromides or chlorides by nucleophilic substitution with sodium azide in DMSO (Alvarez procedure, Eq. 1).[3] Adamantyl azide (Eq. 2),[4] 6-azidohexan-1-ol (Eq. 3),[5] and aryl azides (Eq. 4) were prepared following previously reported procedures.

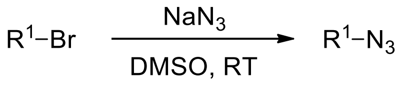

(1)

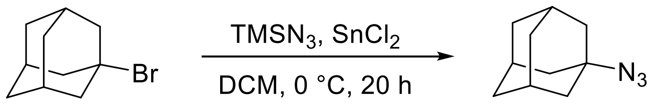

(2)

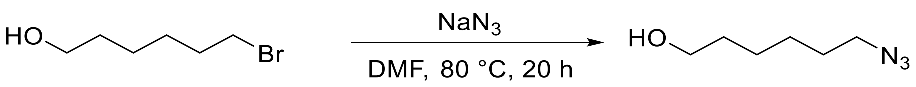

(3)

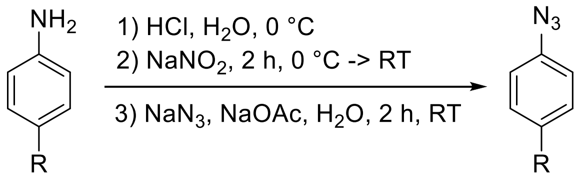

(4)

Benzyl azide

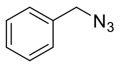

From benzyl bromide (2.40 mL, 20.0 mmol) and following the Alvarez procedure[3] (1 h, RT), the title compound was isolated as a pale yellow oil (2.60 g, 98%) with spectroscopic data in accordance with the literature.[3]

^{1}H NMR (400 MHz, $CDCl_3$) δ 7.43–7.27 (m, 5H), 4.30 (s, 2H); ^{13}C NMR (100 MHz, $CDCl_3$) δ 135.4, 128.9, 128.4, 128.3, 54.8.

4-(Azidomethyl)benzonitrile

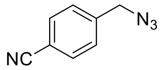

From (4-bromomethyl)benzonitrile (1.96 g, 10 mmol) and following the Alvarez procedure[3] (3 h, RT), the title compound was isolated as a pale yellow oil (1.48 g, 94%) with spectroscopic data in accordance with the literature.[6]

^{1}H NMR (400 MHz, $CDCl_3$) δ 7.68 (d, *J* = 8.5 Hz, 2H), 7.43 (d, *J* = 8.5 Hz, 2H), 4.45 (s, 2H); ^{13}C NMR (100 MHz, $CDCl_3$) δ 140.7, 132.5, 128.4, 112.0, 109.2, 53.9.

Methyl 4-(azidomethyl)benzoate

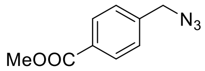

From methyl 4-(bromomethyl)benzoate (3.44 g, 15 mmol) and following the Alvarez procedure[3] (18 h, RT), the title compound was isolated as a white solid (2.29 g, 80%) with spectroscopic data in accordance with the literature.[7]

^{1}H NMR (400 MHz, $CDCl_3$) δ 8.05 (d, *J* = 8.0 Hz, 2H), 7.38 (d, *J* = 8.0 Hz, 2H), 4.41 (s, 2H), 3.92 (s, 3H); ^{13}C NMR (100 MHz, $CDCl_3$) δ 166.9, 140.7, 130.4, 128.2, 54.5, 52.4.

(1-Azidoethyl)benzene

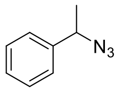

From 1-phenylethylbromide (1.36 mL, 10 mmol) and following the Alvarez procedure[3] (12 h, RT), the title compound was isolated as a pale yellow oil (1.34 g, 91%) with spectroscopic data in accordance with the literature.[3]

^{1}H NMR (400 MHz, $CDCl_3$) δ 7.32–7.27 (m, 5H), 4.59 (q, *J* = 6.9 Hz, 1H), 1.51 (d, *J* = 6.9 Hz, 3H); ^{13}C NMR (100 MHz, $CDCl_3$) δ140.8, 128.7, 128.1, 126.3, 61.1, 21.5.

(2-Azidoethyl)benzene

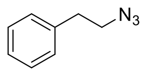

From 2-phenylethyl bromide (1.37 mL, 10.0 mmol) and following the Alvarez procedure[3] (2 h, RT), the title compound was isolated as a pale yellow oil (1.38 g, 94%) with spectroscopic data consistent with the literature.[1,8]

^{1}H NMR (400 MHz, $CDCl_3$) δ 7.36–7.17 (m, 5H), 3.49 (t, *J* = 7.3 Hz, 2H), 2.88 (t, *J* = 7.3 Hz, 2H); ^{13}C NMR (100 MHz, $CDCl_3$) δ 138.0, 128.7, 128.6, 126.7, 52.4, 35.3.

6-Azidohexan-1-ol

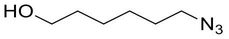

From 6-bromohexan-1-ol (1.96 mL, 15 mmol) and following the Alvarez procedure[3] in DMF (18 h, 80 °C), the title compound was isolated as a colorless oil (1.82 g, 85%) with spectroscopic data in accordance with the literature.[5]

^{1}H NMR (400 MHz, $CDCl_3$) δ 3.65 (t, *J* = 6.0 Hz, 2H), 3.27 (t, *J* = 6.0 Hz, 2H), 1.69–1.53 (m, 4H), 1.47–1.34 (m, 4H); ^{13}C NMR (100 MHz, $CDCl_3$) δ 62.4, 51.2, 32.3, 28.6, 26.3, 25.2.

7-Azidoheptanenitrile

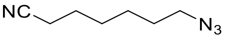

From 7-bromoheptanenitrile (1.67 mL, 11.1 mmol) and following the Alvarez procedure[3] (20 h, RT), the title compound was isolated as a colorless oil (1.66 g, 98%) with spectroscopic data in accordance with the literature.[9]

^{1}H NMR (400 MHz, $CDCl_3$) δ 3.31 (t, *J* = 5.0 Hz, 2H), 2.37 (t, *J* = 6.0 Hz, 2H), 1.76–1.59 (m, 4H), 1.59–1.47 (m, 2H); ^{13}C NMR (100 MHz, $CDCl_3$) δ 120.1, 51.2, 28.4, 26.3, 25.3, 17.4.

1-Azidohexane

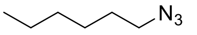

From 1-bromohexane (4.25 mL, 30.3 mmol) and following the Alvarez procedure[3] (18 h, RT), the title compound was isolated as a colorless oil (3.39 g, 88%) with spectroscopic data consistent with the literature.[10,11]

^{1}H NMR (400 MHz, $CDCl_3$) δ 3.26 (t, *J* = 7.0 Hz, 2H), 1.63–1.56 (m, 2H), 1.41–1.25 (m, 6H), 0.90 (t, *J* = 6.9 Hz, 3H); ^{13}C NMR (100 MHz, $CDCl_3$) δ 51.5, 31.3, 28.8, 26.4, 22.5, 14.0.

1-Azidoadamantane

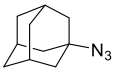

To a stirred solution of 1-bromoadamantane (2.15 g, 10 mmol) in dry DCM (50 mL) at 0 °C under nitrogen atmosphere, azidotrimethylsilane (1.59 mL, 12 mmol) and stannic chloride (1 mL, 1.72 mmol) were added. The mixture was stirred for 12 h before the reaction was quenched with ice-cold water (100 mL) and extracted with DCM (2 x 100 mL). The organic layer was dried over anhydrous magnesium sulphate and concentrated to give a crude white solid which was purified by silica gel column chromatography (hexanes/DCM, 20:1; R_f = 0.43) to give the title compound as a white solid (1.05 g, 5.9 mmol, 59%). Spectroscopic data for the title compound were consistent with the previously reported ones.[12]

^{1}H NMR (400 MHz, $CDCl_3$) δ 2.15 (br s, 3H), 1.84–1.76 (m, 6H), 1.74–1.59 (m, 6H); ^{13}C NMR (100 MHz, $CDCl_3$) δ 59.0, 41.5, 35.9, 29.8.

2-(2-Azidoethyl)-1,3-dioxolane

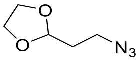

From 2-(2-bromoethyl)-1,3-dioxolane (1.34 mL, 10 mmol) and following the Alvarez procedure[3] (14 h, RT), the title compound was isolated as a light yellow oil (1.41 g, 99%) with spectroscopic data in accordance with the literature.[1,13]

^{1}H NMR (400 MHz, $CDCl_3$) δ 4.96 (t, *J* = 4.5 Hz, 1H), 4.03–3.92 (m, 2H), 3.92–3.82 (m, 2H), 3.43 (t, *J* = 6.9 Hz, 2H), 1.95 (td, *J* = 6.9; 4.5 Hz, 2H); ^{13}C NMR (100 MHz, $CDCl_3$) δ 101.9 (CH), 64.9 (CH_2), 46.5 (CH_2), 33.0 (CH_2).

1-Azido-4-trifluoromethylbenzene

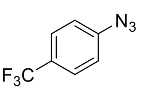

4-Trifluoromethylaniline (10.0 mL, 79.6 mmol) was added to HCl (100 mL, 12 M) in water (100 mL) at 0 °C. A solution of $NaNO_2$ (6.59 g, 95.6 mmol, 1.2 equiv) in water (100 mL) was added portion-wise and the solution was stirred at 0 °C for 2 h. A solution of NaN_3 (7.77 g, 119.5 mmol, 1.5 equiv) in water (50 mL) was added drop-wise at 0 °C (CAUTION: vigorous release of N_2) and the reaction was slowly allowed to warm to room temperature and it was stirred for 2 h. The organic layer was extracted twice with diethyl ether, washed with sodium bicarbonate, dried over $MgSO_4$ and concentrated under reduced

pressure to yield the title compound (12.0 g, 80%) as an orange oil with spectroscopic data in accordance with the literature. [14]
^{1}H NMR (400 MHz, $CDCl_3$) δ 7.61 (d, J = 8.5 Hz, 2H), 7.12 (d, J = 8.5 Hz, 2H); ^{13}C NMR (100 MHz, $CDCl_3$) δ 143.7, 127.1 (q, J = 33.0 Hz), 127.0 (q, J = 3.0 Hz), 124.0 (q, J = 270 Hz), 119.2.

1-Azido-4-nitrobenzene

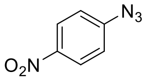

Following the procedure for the preparation of 1-azido-4-trifluoromethylbenzene from 4-nitroaniline (10.0 g, 72.4 mmol), the title compound (11.8 g, 99%) was isolated as a yellow solid with spectroscopic data in accordance with the literature.[15]
^{1}H NMR (400 MHz, $CDCl_3$) δ 8.25 (d, J = 9.0 Hz, 2H), 7.14 (d, J = 9.0 Hz, 2H); ^{13}C NMR (100 MHz, $CDCl_3$) δ 146.9, 144.6, 125.6, 119.4.

1-Azido-4-methoxybenzene

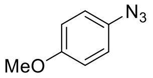

Following the procedure for the preparation of 1-azido-4-trifluoromethylbenzene from 4-methoxyaniline (10.0 g, 81.2 mmol), the title compound (11.3 g, 93%) was isolated as a red oil with spectroscopic data in accordance with the literature.[16]
^{1}H NMR (400 MHz, $CDCl_3$) δ 6.96 (d, J = 9.0 Hz, 2H), 6.89 (d, J = 9.0 Hz, 2H), 3.79 (s, 3H); ^{13}C NMR (100 MHz, $CDCl_3$) δ 157.0, 132.3, 120.0, 115.1, 55.5.

1-Azido-4-methylbenzene

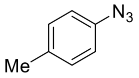

Following the procedure for the preparation of 1-azido-4-trifluoromethylbenzene from 4-methylaniline (10.0 g, 93.4 mmol), the title compound (11.0 g, 89%) was isolated as a brown solid with spectroscopic data in accordance with the literature.[17]
^{1}H NMR (400 MHz, $CDCl_3$) δ 7.16 (d, J = 8.5 Hz, 2H), 6.93 (d, J = 8.5 Hz, 2H), 2.33 (s, 3H); ^{13}C NMR (400 MHz, $CDCl_3$) δ 137.1, 134.6, 130.4, 118.9, 20.9.

4. PREPARATION OF [1,2,3]-TRIAZOLES (1)

General Procedure: In a vial fitted with a screw cap, [CuI(Mes-6)] (0.5–0.1 mol%), azide (0.5 mmol) and alkyne (0.5 mmol) were loaded. The reaction was allowed to proceed at room temperature until full (or no further) conversion was observed by ^{1}H NMR. Then, saturated aqueous ammonium chloride solution (10 mL) was added and the resulting mixture was stirred vigorously for 3 h. The resulting precipitate was filtered and washed with water and pentane, then dried under reduced pressure. In all examples, the crude products were estimated to be >95% pure by ^{1}H NMR.

CAUTION: Although we did not experience any problems, the cycloaddition of azides and alkynes is highly exothermic and, as a consequence, adequate cooling should always be available when performing these reactions in the absence of solvent.

1-Benzyl-4-phenyl-1*H*-[1,2,3]triazole (1a)

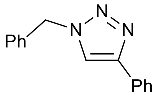

Using the general procedure from 62 μL (0.5 mmol) of benzyl azide and 55 μL (0.5 mmol) of phenylacetylene with 0.1 mol% [Cu], the title compound (0.107 g, 91%) was isolated after 1 h as an off white solid with spectroscopic data in accordance with the literature.[18]

^{1}H NMR (400 MHz, $CDCl_3$) δ 7.83–7.78 (m, 2H), 7.67 (s, 1H), 7.44–7.36 (m, 5H), 7.35–7.29 (m, 3H), 5.59 (s, 2H); ^{13}C NMR (400 MHz, $CDCl_3$) δ 148.1, 134.7, 130.5, 129.1, 128.8, 128.7, 128.1, 128.0, 125.6, 119.6, 54.1.

1-Benzyl-4-butyl-1*H*-[1,2,3]triazole (1b)

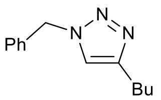

Using the general procedure from 62 μL (0.5 mmol) of benzyl azide and 59 μL (0.5 mmol) of 1-hexyne with 0.3 mol% [Cu], the title compound (0.096 g, 90%) was isolated after 6 h as an off white solid with spectroscopic data in accordance with the literature.[19]

^{1}H NMR (400 MHz, $CDCl_3$) δ 7.41–7.33 (m, 3H), 7.28–7.24 (m, 2H), 7.19 (s, 1H), 5.50 (s, 2H, PhCH2), 2.70 (t, J = 7.6 Hz, 2H), 1.69–1.57 (m, 2H), 1.41–1.30 (m, 2H), 0.92 (t, J = 7.4 Hz); ^{13}C NMR (400 MHz, $CDCl_3$) δ 148.9, 135.0, 129.1, 128.6, 128.0, 121.0, 54.0, 31.4, 25.4, 22.3, 13.8.

1-Benzyl-4-(trimethylsilyl)-1*H*-[1,2,3]triazole (1c)

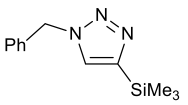

Using the general procedure from 62 μL (0.5 mmol) of benzyl azide and 71 μL (0.5 mmol) of trimethylsilylacetylene with 0.5 mol% [Cu], the title compound (0.105 g, 85%) was isolated after 24 h as an off white solid with spectroscopic data in accordance with the literature.[18]

^{1}H NMR (400 MHz, $CDCl_3$) δ 7.40 (s, 1H), 7.39–7.34 (m, 3H), 7.29–7.25 (m, 2H), 5.54 (s, 2H), 0.28 (s, 9H); ^{13}C NMR (100 MHz, $CDCl_3$) δ 147.0, 134.9, 129.0, 128.7, 128.5, 128.0, 53.4, -1.2.

2-(1-Benzyl-1*H*-1,2,3-triazol-4-yl)propan-2-ol (1d)

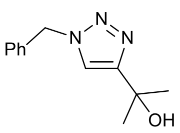

Using the general procedure from 62 μL (0.5 mmol) of benzyl azide and 49 μL (0.5 mmol) of 2-methyl-3-butyn-2-ol with 0.3 mol% [Cu], the title compound (0.099g, 97%) was isolated after 16 h as an off white solid with spectroscopic data in accordance with the literature.[20]

^{1}H NMR (400 MHz, $CDCl_3$) δ 7.44–7.36 (m, 3H), 7.35 (s, 1H), 7.33–7.26 (m, 2H), 5.51 (s, 2H), 2.34 (bs, 1H), 1.62 (s, 6H); ^{13}C NMR (100 MHz, $CDCl_3$) δ 156.1, 134.6, 129.1, 128.7, 128.1, 119.1, 68.5, 54.1, 30.4.

4-[(4-Phenyl-1*H*-1,2,3-triazol-1-yl)methyl]benzonitrile (1e)

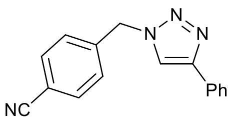

A) Using the general procedure from 72 μL (0.5 mmol) of 4-(azidomethyl)benzonitrile and 55 μL (0.5 mmol) of phenylacetylene with 0.5 mol% [Cu], the title compound (0.120 g, 98%) was isolated after 4 h as an off white solid with spectroscopic data in accordance with the literature.[188]

B) Using the general procedure from 144 μL (1.0 mmol) of 4-(azidomethyl)benzonitrile and 110 μL (1.0 mmol) of phenylacetylene with 0.1 mol% [Cu], the title compound (0.247g, 95%) was isolated after 24 h as an off white solid with spectroscopic data in accordance with the literature.[18]

^{1}H NMR (400 MHz, $CDCl_3$) δ 7.81 (d, *J* = 8.0 Hz, 2H), 7.73 (s, 1H), 7.68 (d, *J* = 8.0 Hz, 2H), 7.44–7.32 (m, 5H), 5.65 (s, 2H); ^{13}C NMR (100 MHz, $CDCl_3$) δ 134.5, 129.1, 128.8, 128.1, 68.4, 54.2, 30.4.

Methyl 4-[(4-phenyl-1H-1,2,3-triazol-1-yl)methyl]benzoate (1f)

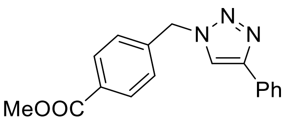

Using the general procedure from 191 mg (1.0 mmol) of methyl 4-(azidomethyl)benzoate and 110 μL (1.0 mmol) of phenylacetylene with 0.5 mol% [Cu], the title compound (0.258 g, 88%) was isolated after 24 h as an off white solid.

m.p. 175.5–178.5 °C; ν_{max} (film) 1712, 1613 cm^{-1}; ^{1}H NMR (400 MHz, $CDCl_3$) δ 8.05 (d, *J* = 8.5 Hz, 2H), 7.81 (d, *J* = 8.5 Hz, 2H), 7.70 (s, 1H), 7.43–7.39 (m, 2H), 7.37–7.31 (m, 3H), 5.64 (s, 2H), 3.92 (s, 3H); ^{13}C NMR (100 MHz, $CDCl_3$) δ 166.4, 148.4, 139.6, 130.5, 130.4, 130.3, 128.8, 128.3, 127.8, 125.7, 119.7, 53.7, 52.3; HRMS (ES) calculated for $C_{17}H_{16}N_3O_2$: 294.1243, found: 294.1248 [(M+H)$^+$].

4-Cyclopropyl-1-(1-phenylethyl)-1*H*-[1,2,3]triazole (1g)

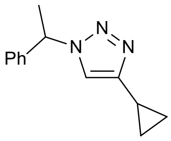

Using the general procedure from 147 mg (1.0 mmol) of (1-azidoethyl)benzene and 85 μL (1.0 mmol) of cyclopropylacetylene azide with 0.3 mol% [Cu], the title compound (0.168 g, 79%) was isolated after 24 h as an off white solid with spectroscopic data in accordance with the literature.[11]

^{1}H NMR (400 MHz, $CDCl_3$) δ 7.41–7.28 (m, 3H), 7.28–7.23 (m, 2H), 7.11 (s, 1H), 5.76 (q, *J* = 7.1 Hz, 1H), 1.95 (d, *J* = 7.1 Hz, 3H), 1.95–1.87 (m, 1H), 0.95–0.85 (m, 2H), 0.85–0.79 (m, 2H); ^{13}C NMR (100 MHz, $CDCl_3$) δ 150.1, 140.2, 128.9, 128.3, 126.4, 118.4, 59.9, 21.2, 7.7, 6.8.

Dimethyl-(1-phenethyl-1*H*-[1,2,3]triazol-4-ylmethyl) amine (1h)

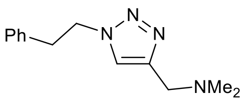

Using the general procedure from 74 mg (0.5 mmol) of (2-azidoethyl)benzene and 55 μL (0.5 mmol) of dimethylprop-2-ynyl amine with 0.1 mol % [Cu], the title compound (0.102 g, 88%) was isolated after 24 h as a light brown oil with spectroscopic data in accordance with the literature.[1]

^{1}H NMR (400 MHz, $CDCl_3$) δ 7.32–7.23 (m, 3H), 7.22 (s, 1H), 7.13–7.07 (m, 2H), 4.59 (t, *J* = 7.2 Hz, 2H), 3.58 (s, 2H), 3.21 (t, *J* = 7.2 Hz, 2H), 2.24 (s, 6H); ^{13}C NMR (100 MHz, $CDCl_3$) δ 144.5, 137.1, 128.7, 128.7, 127.0, 123.0, 54.0, 51.6, 44.9, 36.7.

Ethyl 1-(2-cyanohexyl)-1*H*-[1,2,3]triazole-4-carboxylate (1i)

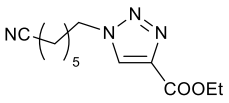

Using the general procedure from 152 mg (1.0 mmol) of 7-azidoheptanenitrile and 102 μL (1.0 mmol) of ethyl propiolate with 0.5 mol% [Cu], the title compound (0.214 g, 86%) was isolated after 8 h as a pale brown solid.

m.p. 74.0–76.5 °C; ν_{max} (film) 2245, 1720, 1539 cm^{-1}; ^{1}H NMR (400 MHz, $CDCl_3$) δ 8.13 (s, 1H), 4.47–4.40 (m, 4H), 2.36 (t, *J* = 7.0 Hz, 2H), 2.01–1.94 (m, 2H), 1.70–1.63 (m, 2H), 1.55–1.48 (m, 2H), 1.43–1.34 (m, 5H); ^{13}C NMR (100 MHz, $CDCl_3$) δ 160.8, 140.3, 127.4, 119.6, 61.3, 50.4, 29.9, 28.0, 25.6, 25.1, 17.0, 14.3; HRMS (ES) calculated for $C_{12}H_{19}N_4O_2$: 251.1508, found: 251.1517 [$(M+H)^+$].

4-(*tert*-Butyl)-1-hexyl-1*H*-[1,2,3]-triazole (1j)

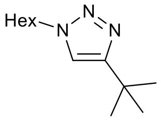

Using the general procedure from 64 mg (0.5 mmol) of hexyl azide and 62 μL (0.5 mmol) of 3,3-dimethylbutyne with 0.5 mol% [Cu], the title compound (0.075 g, 71%) was isolated after 7 h as a yellow oil.

ν_{max} (film) 1542 cm^{-1}; ^{1}H NMR (400 MHz, $CDCl_3$) δ 7.23 (s, 1H), 4.31–4.26 (m, 2H), 1.90–1.87 (m, 2H), 1.35 (s, 9H), 1.34–1.27 (m, 6H), 0.88 (t, *J* = 6.5 Hz, 3H); ^{13}C NMR (100 MHz, $CDCl_3$) δ 157.6, 118.3, 50.1, 31.2, 30.7, 30.4, 30.3, 26.2, 22.4, 13.9; HRMS (ES) calculated for $C_{12}H_{24}N_3$: 210.1970, found: 210.1964 [$(M+H)^+$].

1-Hexyl-4-phenyl-1*H*-[1,2,3]-triazole (1k)

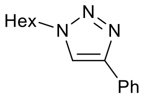

Using the general procedure from 127 mg (1.0 mmol) of hexyl azide and 110 μL (1.0 mmol) of phenylacetylene with 0.1 mol% [Cu], the title compound (0.214 g, 93%) was isolated after 10 h as a white solid with spectroscopic data in accordance with the literature.[21]

^{1}H NMR (400 MHz, $CDCl_3$) δ 7.85–7.83 (m, 2H), 7.74 (s, 1H), 7.44–7.40 (m, 2H), 7.34–7.31 (m, 1H), 4.39 (t, *J* = 7.0 Hz, 2H), 1.98–1.90 (m, 2H), 1.33–1.30 (m, 6H), 0.88 (t, *J* = 7.0 Hz, 3H); ^{13}C NMR (100 MHz, $CDCl_3$) δ 147.7, 130.8, 128.8, 128.0, 125.7, 119.5, 50.4, 31.2, 30.3, 26.2, 22.4, 13.9.

Dimethyl-(1-hexyl-1*H*-[1,2,3]triazol-4-yl) amine (1l)

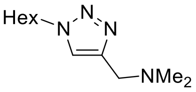

Using the general procedure from 127 mg (1.0 mmol) of hexyl azide and 108 μL (1.0 mmol) of dimethylprop-2-ynyl amine with 0.5 mol% [Cu], the title compound (0.214 g, 93%) was isolated after 4 h as a yellow oil.

ν_{max} (film) 1457 cm^{-1}; ^{1}H NMR (400 MHz, $CDCl_3$) δ 7.48 (s, 1H), 4.34 (t, *J* = 7.0 Hz, 2H), 3.61 (s, 2H), 2.40–2.20 (br m, 6H), 1.94–1.85 (m, 2H), 1.30 (s, 6H), 0.88 (br t, *J* = 6.5 Hz, 3H); ^{13}C NMR (100 MHz, $CDCl_3$) δ 145.1, 122.2, 54.5, 50.3, 45.1, 31.1, 30.2, 26.2, 22.4, 13.9; HRMS (ES) calculated for $C_{11}H_{23}N_4$: 211.1923, found: 211.1914 [$(M+H)^+$].

Ethyl 1-(adamant-1-yl)-1*H*-[1,2,3]-triazole-4-carboxylate (1m)

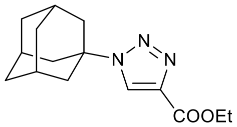

A) Using the general procedure from 89 mg (0.5 mmol) of adamantyl azide and 51 µL (1.0 mmol) of ethyl propiolate with 0.3 mol% [Cu], the title compound (0.102 g, 74%) was isolated after 48 h as a pale orange solid with spectroscopic data in accordance with the literature.[12]

B) Using the general procedure from 89 mg (0.5 mmol) of adamantyl azide and 51 µL (0.5 mmol) of ethyl propiolate with 0.1 mol% [Cu], the title compound (0.106 g, 81%) was isolated after 72 h as a pale orange solid with spectroscopic data in accordance with the literature.[12]

^{1}H NMR (400 MHz, $CDCl_3$) δ 8.14 (s, 1H), 4.42 (q, J = 7.0 Hz, 2H), 2.32–2.25 (m, 9H), 1.84–1.76 (m, 6H), 1.41 (t, J = 7.0 Hz, 3H); ^{13}C NMR (100 MHz, $CDCl_3$) δ 161.2, 139.3, 124.1, 61.1, 60.4, 42.9, 35.8, 29.4, 14.4.

Ethyl 1-(2-(1,3-dioxolan-2-yl)ethyl)-1*H*-1,2,3-triazole-4-carboxylate (1n)

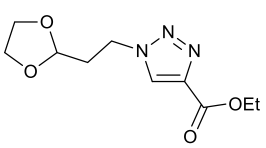

Using the general procedure from 72 mg (0.5 mmol) of 2-(2-azidoethyl)-1,3-dioxolane and 50 µL (0.5 mmol) of ethyl propiolate with 0.1 mol% [Cu], the title compound (0.115 g, 93%) was isolated after 22 h as a light yellow oil with spectroscopic data in accordance with the literature.[22]

^{1}H NMR (400 MHz, $CDCl_3$) δ 8.13 (s, 1H), 4.93 (t, J = 4.0 Hz, 1H), 4.58 (t, J = 7.0 Hz, 2H), 4.44 (q, J = 7.0 Hz, 2H), 4.04–3.94 (m, 2H), 3.94–3.84 (m, 2H), 2.34 (dt, J = 7.0; 4.0 Hz, 2H), 1.42 (t, J = 7.0 Hz, 3H); ^{13}C NMR (100 MHz, $CDCl_3$) δ 160.8, 140.2, 127.7, 101.3, 65.1, 61.2, 45.5, 33.7, 14.3.

2-[1-(4-Trifluoromethylphenyl)-1*H*-[1,2,3]-triazol-4-yl]propan-2-ol (1o)

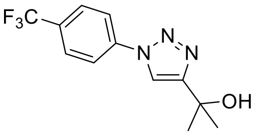

Using the general procedure from 140 µL (1.0 mmol) of 4-trifluoromethylphenyl azide and 97 µL (1.0 mmol) of 3,3-dimethyl-1-propynol with 0.5 mol% [Cu], the title compound (0.239 g, 88%) was isolated after 8 h as a pale yellow solid.

m.p. 168.0–175.5°C; ν_{max} (film) 3244, 1618, 1530 cm^{-1}; ^{1}H NMR (400 MHz, $CDCl_3$) δ 7.99 (s, 1H), 7.90 (d, J = 8.5 Hz, 2H), 7.80 (d, J = 8.5 Hz, 2H), 2.62 (s, 1H), 1.73 (s, 3H); ^{13}C NMR (100 MHz, $CDCl_3$) δ 156.9, 139.5, 130.7 (q, J = 33.5 Hz), 127.1 (q, J = 3.0 Hz), 123.5 (q, J = 271 Hz), 120.4, 117.4, 68.8, 30.5; ^{19}F NMR (377 MHz, $CDCl_3$) δ -62.7; HRMS (ES) calculated for $C_{12}H_{13}N_3OF_3$: 272.1011, found: 272.1010 [$(M+H)^+$].

1-(4-Nitrophenyl)-4-phenyl-1*H*-[1,2,3]-triazole (1p)

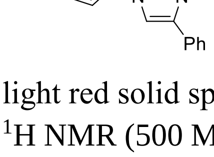

Using the general procedure from 82 mg (0.5 mmol) of 1-azido-4-trifluoromethylbenzene and 55 µL (0.5 mmol) of phenylacetylene with 0.5 mol% [Cu], the title compound (0.123 g, 92%) was isolated after 2 h as a light red solid spectroscopic data in accordance with the literature.[23]

^{1}H NMR (500 MHz, DMSO-d6, 110 °C): 9.26 (s, 1H), 8.48–8.41 (m, 2H), 8.28–8.21 (m, 2H), 7.99–7.91 (m, 2H), 7.54–7.38 (m, 2H), 7.44–7.38 (m, 1H); ^{13}C NMR (125 MHz, DMSO-d6, 110°C): 147.5, 146.6, 140.5, 129.5, 128.3, 127.8, 125.09, 124.8, 120.2, 119.3.

1-(4-Trifluoromethylphenyl)-4-phenyl-1*H*-[1,2,3]-triazole (1q)

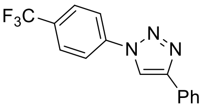

Using the general procedure from 94 mg (0.5 mmol) of 1-azido-4-trifluoromethylbenzene and 55 μL (0.5 mmol) of phenylacetylene with 0.5 mol% [Cu], the title compound (0.133 g, 92%) was isolated after 8 h as an off-white solid spectroscopic data in accordance with the literature.[24]

^{1}H NMR (500 MHz, DMSO-d6, 110 °C): 9.21 (s, 1H, CH), 8.22–8.16 (m, 2H), 8.00–7.92 (m, 4H), 7.53–7.46 (m, 2H), 7.43–7.37 (m, 1H); ^{13}C NMR (125 MHz, DMSO-d6, 110°C): 147.3, 139.1, 129.7, 128.5 (q, *J* = 31.3 Hz), 128.2, 127.7, 126.4, 125.1, 124.3 (q, *J* = 271.2 Hz), 120.1, 119.1.

1-(4-Methoxyphenyl)-4-phenyl-1H-[1,2,3]-triazole (1r)

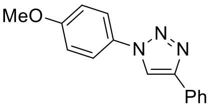

Using the general procedure from 76 mg (0.5 mmol) of 1-azido-4-methoxybenzene and 55 μL (0.5 mmol) of phenylacetylene with 0.3 mol% [Cu], the title compound was isolated after 2 h as an off-white solid (0.111 g, 88%) with spectroscopic data in accordance with the literature.[25]

^{1}H NMR (400 MHz, $CDCl_3$) δ 8.12 (s, 1H), 7.91 (d, *J* = 8.0 Hz, 2H), 7.68 (d, *J* = 8.0 Hz, 2H), 7.46 (t, *J* = 8.0 Hz, 3H), 7.39 (t, *J* = 8.0 Hz, 1H), 7.06–7.00 (d, *J* = 8.0 Hz, 2H), 3.87 (s, 3H); ^{13}C NMR (100 MHz, $CDCl_3$) δ 159.8, 148.1, 130.5, 130.3, 128.8, 128.3, 125.8, 122.1, 117.8, 114.7, 55.6.

1-(4-Methylphenyl)-4-phenyl-1H-[1,2,3]-triazole (1s)

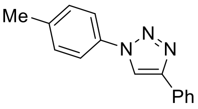

Using the general procedure from 67 mg (0.5 mmol) of 1-azido-4-methylbenzene and 55 μL (0.5 mmol) of phenylacetylene with 0.3 mol% [Cu], the title compound was isolated after 8 h as an off-white solid (0.107 g, 91%) with spectroscopic data in accordance with the literature.[26]

^{1}H NMR (400 MHz, $CDCl_3$) δ 8.16 (s, 1H), 7.92 (d, *J* = 8.0 Hz, 2H), 7.67 (d, *J* = 8.0 Hz, 2H), 7.46 (t, *J* = 8.0 Hz, 3H), 7.40–7.37 (m, 1H), 7.36–7.30 (m, 2H), 2.44 (s, 3H); ^{13}C NMR (100 MHz, $CDCl_3$) δ 148.2, 138.8, 134.7, 130.3, 130.2, 128.8, 128.3, 125.8, 123.4, 117.6, 21.1.

6. REFERENCES

[1] S. Díez-González, A. Correa, L. Cavallo, S. P. Nolan, *Chem. Eur. J.* **2006**, *12*, 7558–7564.

[2] L. R. Collins, T. M. Rookes, M. F. Mahon, I. M. Riddlestone, M. K. Whittlesey, *Organometallics* **2014**, *33*, 5882–5887.

[3] S. G. Alvarez, M. T. Alvarez, *Synthesis* **1997**, 413–414.

[4] G. K. S. Prakash, M. A. Stephenson, J. G. Shih, G. A. Olah, *J. Org. Chem.* **1986**, *51*, 3215–3217.

[5] H. Li, A. C. Fahrenbach, A. Coskun, Z. Zhu, G. Barin, Y.-L. Zhao, Y. Y. Botros, J.-P. Sauvage, J. F. Stoddart, *Angew. Chem. Int. Ed.* **2011**, *50*, 6782–6788.

[6] U. Sirion, H. J. Kim, J. H. Lee, J. W. Seo, B. S. Lee, S. J. Lee, S. J. Oh, D. Y. Chi, *Tetrahedron Lett.* **2007**, *48*, 3953–3957.

[7] M. Malkoch, K. Schleicher, E. Drockenmuller, C. J. Hawker, T. P. Russell, P. Wu, V. V. Fokin, *Macromolecules* **2005**, *38*, 3663–3678.
[8] M. Ito, K.-i. Koyakumaru, T. Ohta, H. Takaya, *Synthesis* **1995**, 376–378.
[9] L. Luo, C. Wilhelm, A. Sun, C. P. Grey, J. W. Lauher, N. S. Goroff, *J. Am. Chem. Soc.* **2008**, *130*, 7702–7709.
[10] Y. Masuda, M. Hoshi, A. Arase, *Bull. Chem. Soc. Jpn.* **1984**, *57*, 1026–1030.
[11] S. Lal, S. Díez-González, *J. Org. Chem*. **2011**, *76*, 2367–2373.
[12] D. Margosian, P. Kovacic, *J. Org. Chem.* **1981**, *46*, 877–880.
[13] B. Carboni, M. Vaultier, R. Carrié, *Tetrahedron* **1987**, *43*, 1799–1810.
[14] C. C. Ciocoiu, N. Nikolić, H. H. Nguyen, G. H. Thoresen, A. J. Aasen and T. V. Hansen, *Eur. J. Med. Chem.* **2010**, *45*, 3047–3055.
[15] A. W. Gann, J. W. Amoroso, V. J. Einck, W. P. Rice, J. J. Chambers and N. A. Schnarr, *Org. Lett.* **2014**, *16*, 2003–2005.
[16] L. Benati, G. Bencivenni, R. Leardini, M. Minozzi, D. Nanni, R. Scialpi, P. Spagnolo, G. Zanardi, *J. Org. Chem.* **2006**, *71*, 5822–5825.
[17] H. Yang, Y. Li, M. Jiang, J. Wang, H. Fu, *Chem. Eur. J.* **2011**, *17*, 5652–5660.
[18] P. Appukkuttan, W. Dehaen, V. V. Fokin and E. Van der Eycken, *Org. Lett.* **2004**, *6*, 4223–4225.
[19] N. Candelon, D. Lastécouères, A. K. Diallo, J. R. Aranzaes, D. Astruc, J. M. Vincent, *Chem. Commun.* **2008**, 741–743.
[20] A. Cwiklicki, K. Rehse, *Arch. Pharm*. **2004**, *337*, 156–163.
[21] L. Huang, W. Liu, J. Wu, Y. Fu, K. Wang, C. Huo, Z. Du, *Tetrahedron Lett*. **2014**, *55*, 2312–2316.
[22] S. Díez-González, S. P. Nolan, *Angew. Chem. Int. Ed.* **2008**, *47*, 8881–8884.
[23] K. Barral, A. D. Moorhouse, J. E. Moses, *Org. Lett.* **2007**, *9*, 1809–1811.
[24] D. B. Ramachary, A. B. Shashank, S. Karthik, *Angew. Chem. Int. Ed.* **2014**, *53*, 10420–10424.
[25] Z.-X. Wang, H.-L. Qin, *Chem. Commun.* **2003**, 2450–2451.
[26] D. Kumar, V. B. Reddy, *Synthesis* **2010**, 1687–1691.

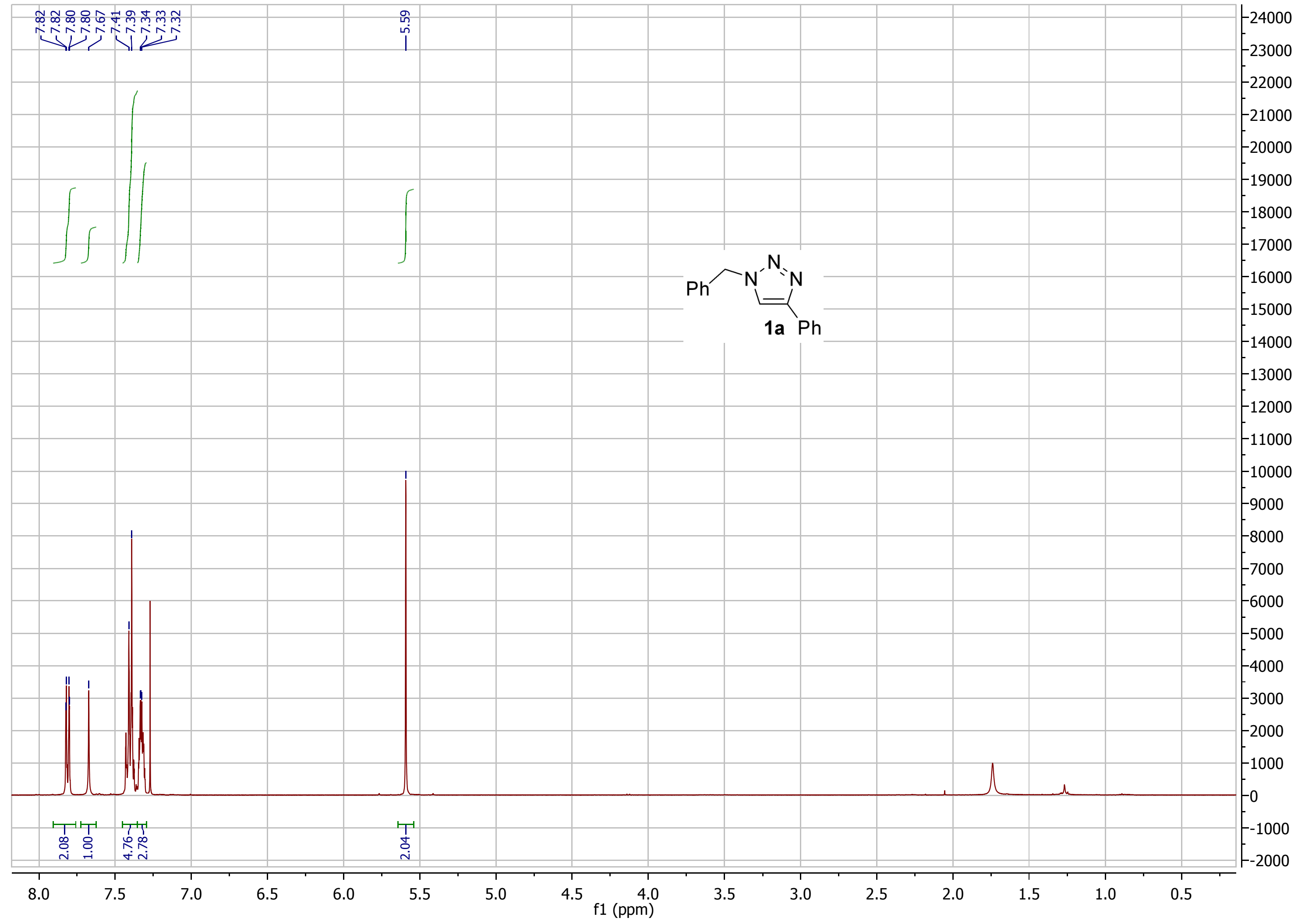
7.82
7.82
7.80
7.80
7.67
7.41
7.39
7.34
7.33
7.32
5.59
Ph
N
N
N
1a
Ph
2.08
1.00
4.76
2.78
2.04
f1 (ppm)

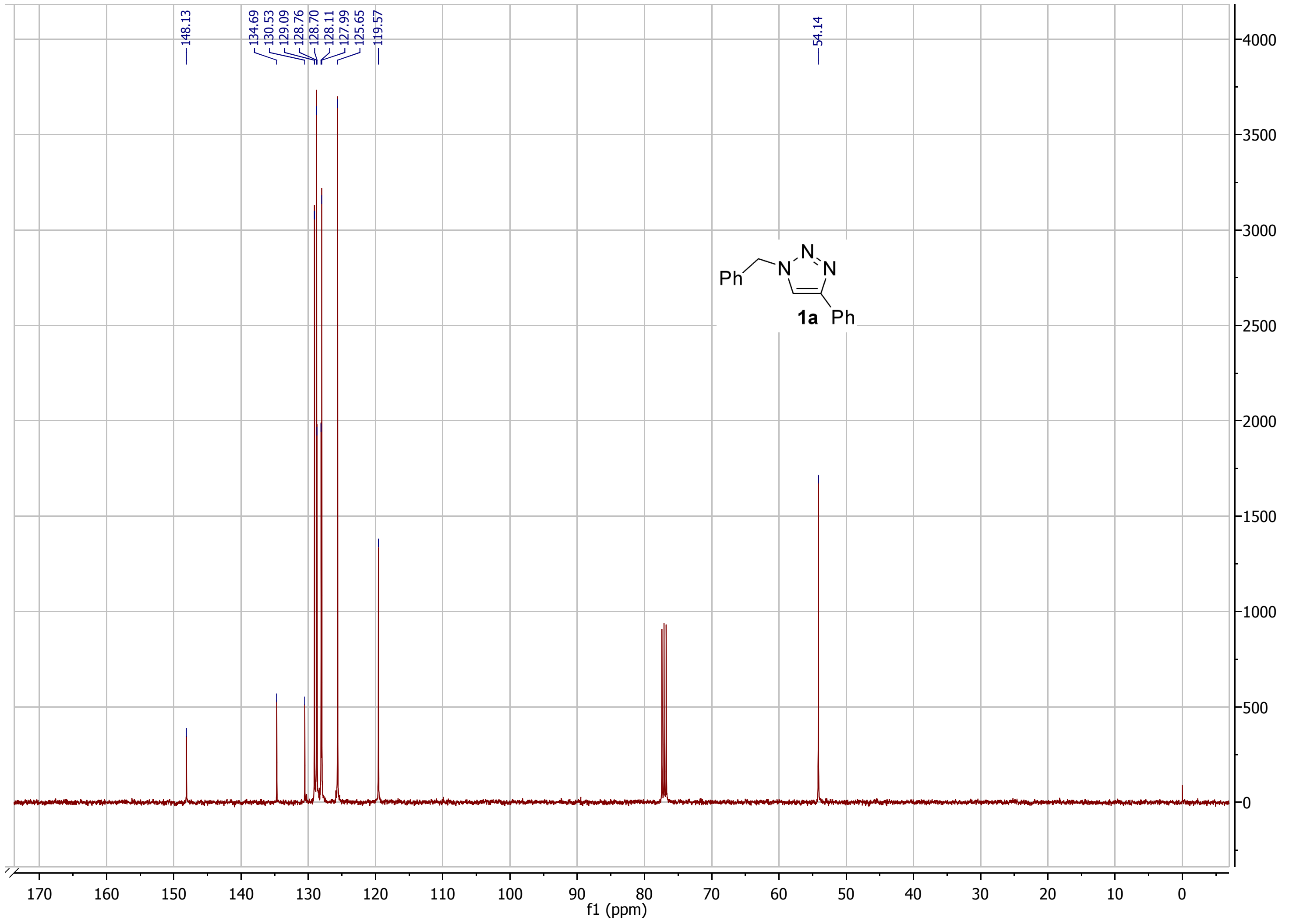
148.13
134.69
130.53
129.09
128.76
128.70
128.11
127.99
125.65
119.57
54.14
Ph
N
N
N
1a
Ph
f1 (ppm)

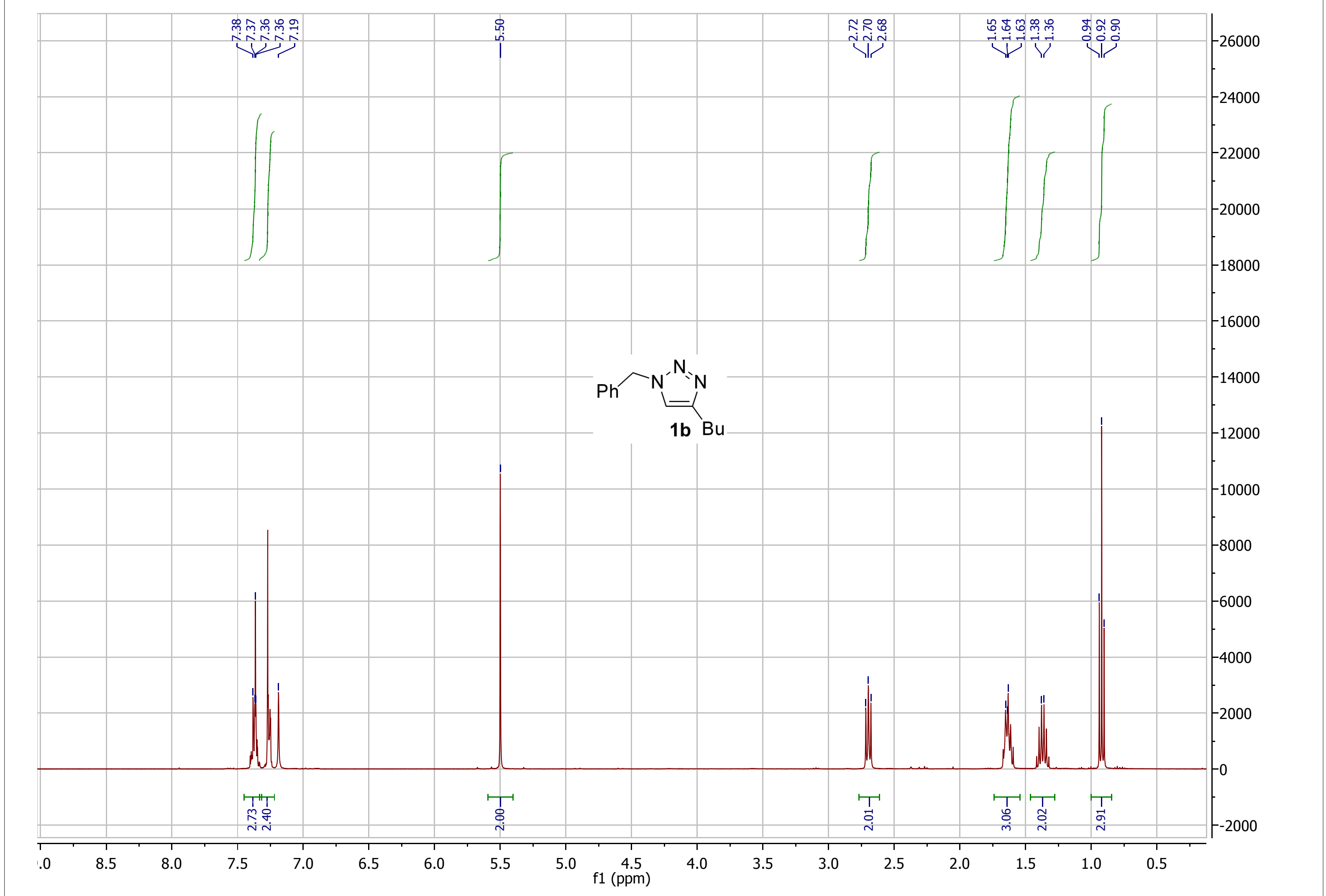
7.38
7.37
7.36
7.36
7.19
5.50
2.72
2.70
2.68
1.65
1.64
1.63
1.38
1.36
0.94
0.92
0.90
Ph
N
N
N
1b
Bu
2.73
2.40
2.00
2.01
3.06
2.02
2.91
f1 (ppm)

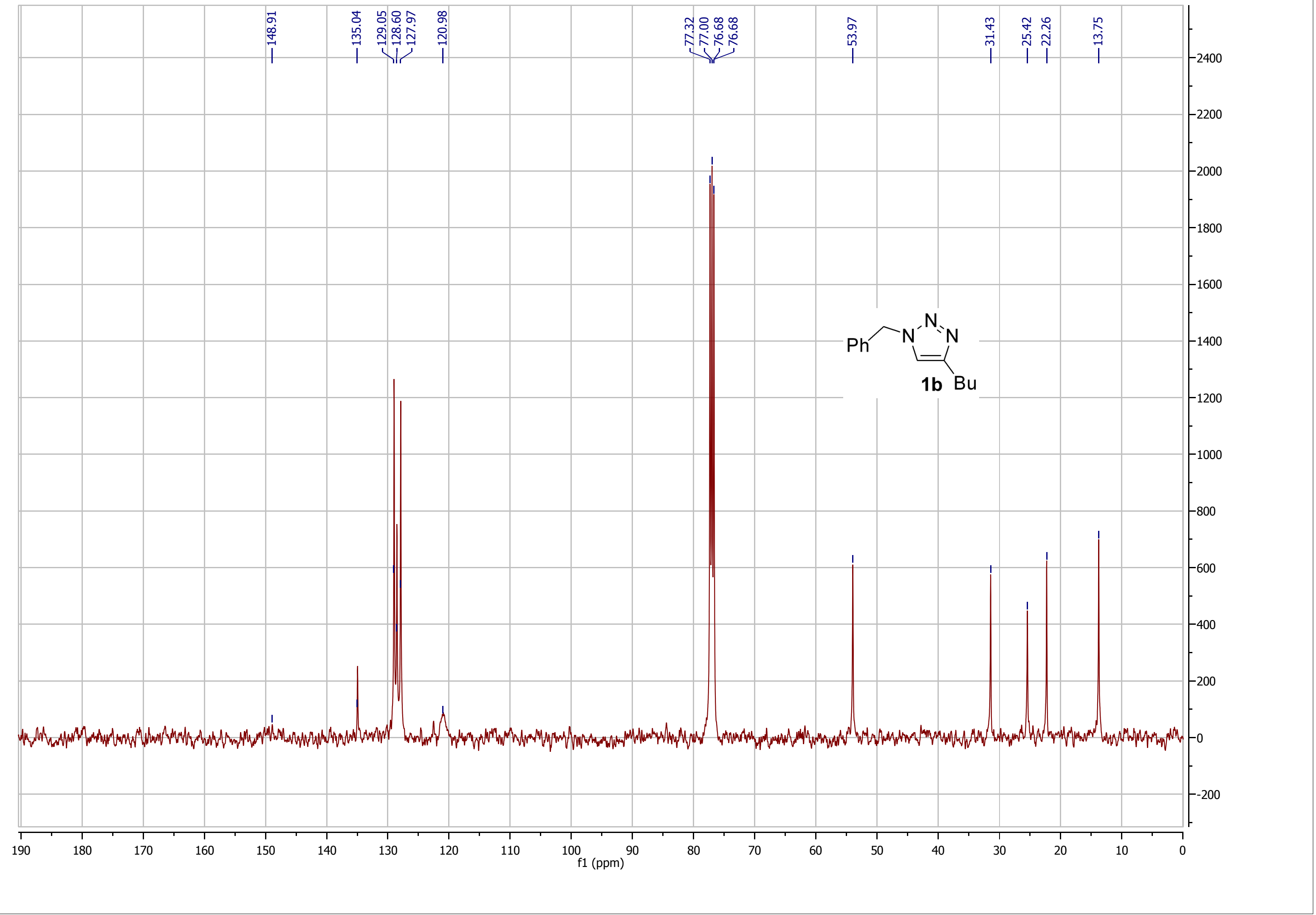
148.91
135.04
129.05
128.60
127.97
120.98
77.32
77.00
76.68
76.68
53.97
31.43
25.42
22.26
13.75
Ph
N
N
N
1b
Bu
f1 (ppm)

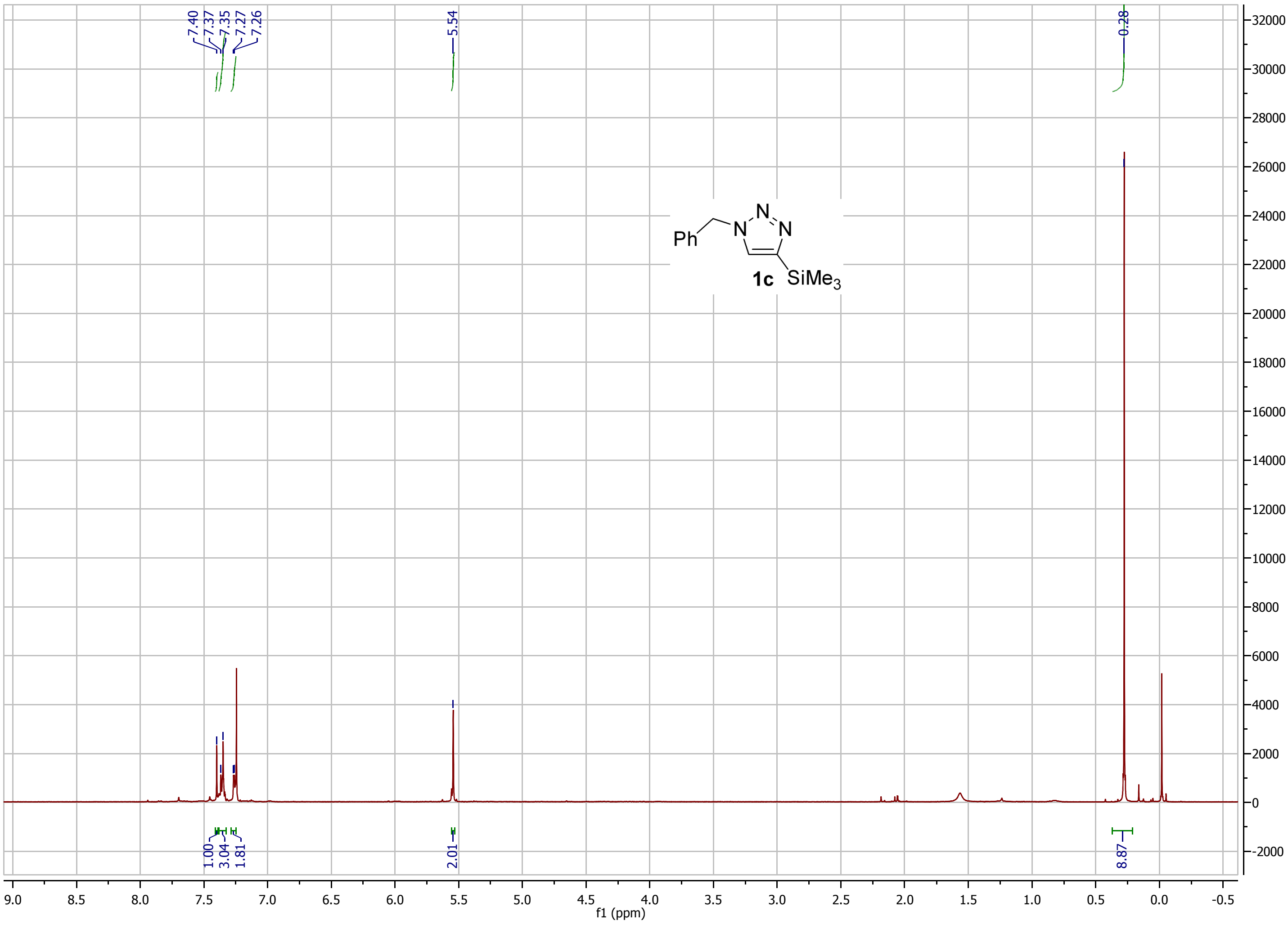

7.40
7.37
7.35
7.27
7.26
5.54
0.28
Ph
N
N
N
1c
SiMe3
1.00
3.04
1.81
2.01
8.87
f1 (ppm)

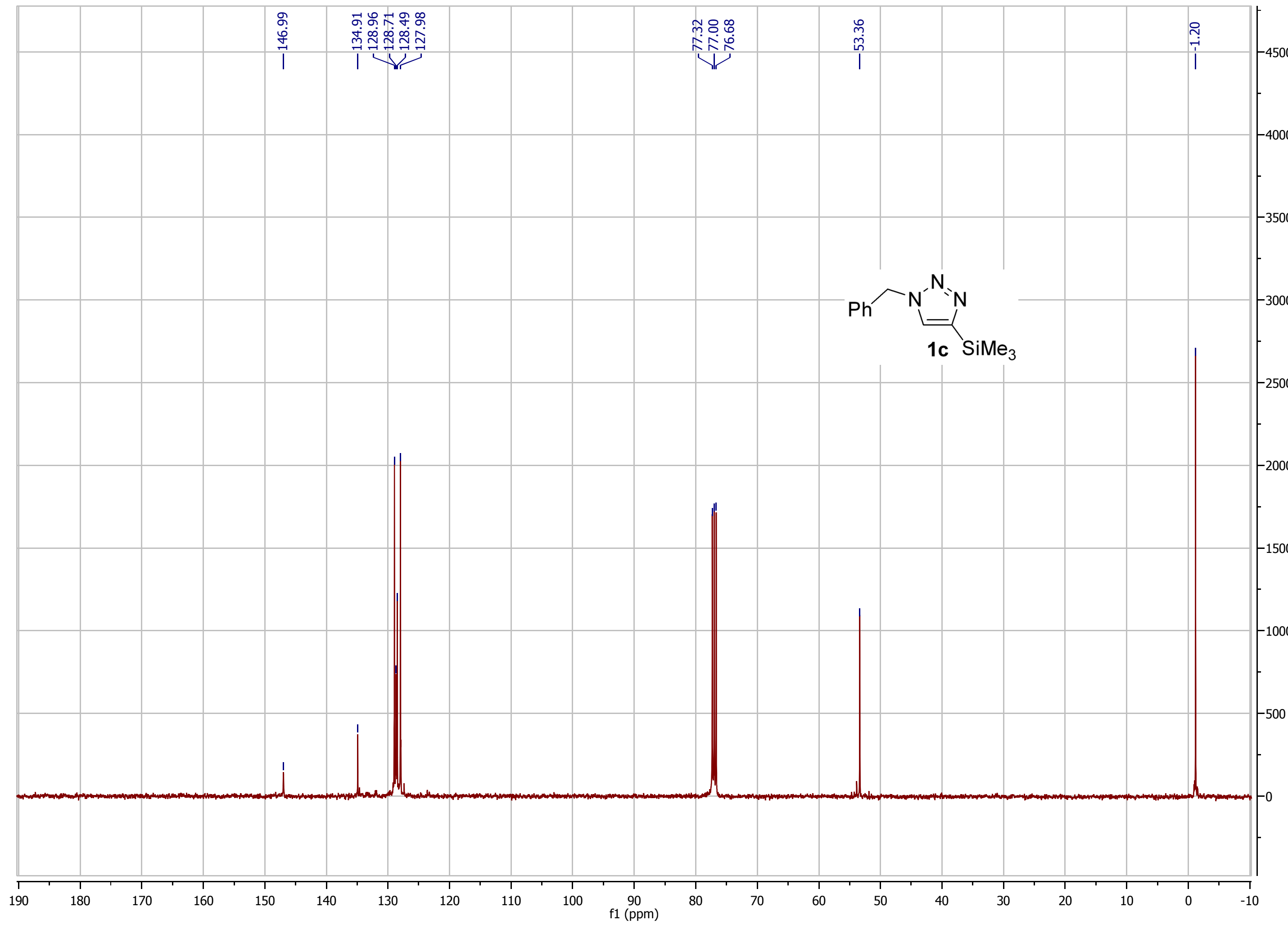

146.99
134.91
128.96
128.71
128.49
127.98
77.32
77.00
76.68
53.36
-1.20
Ph
N
N
N
1c
SiMe3
f1 (ppm)

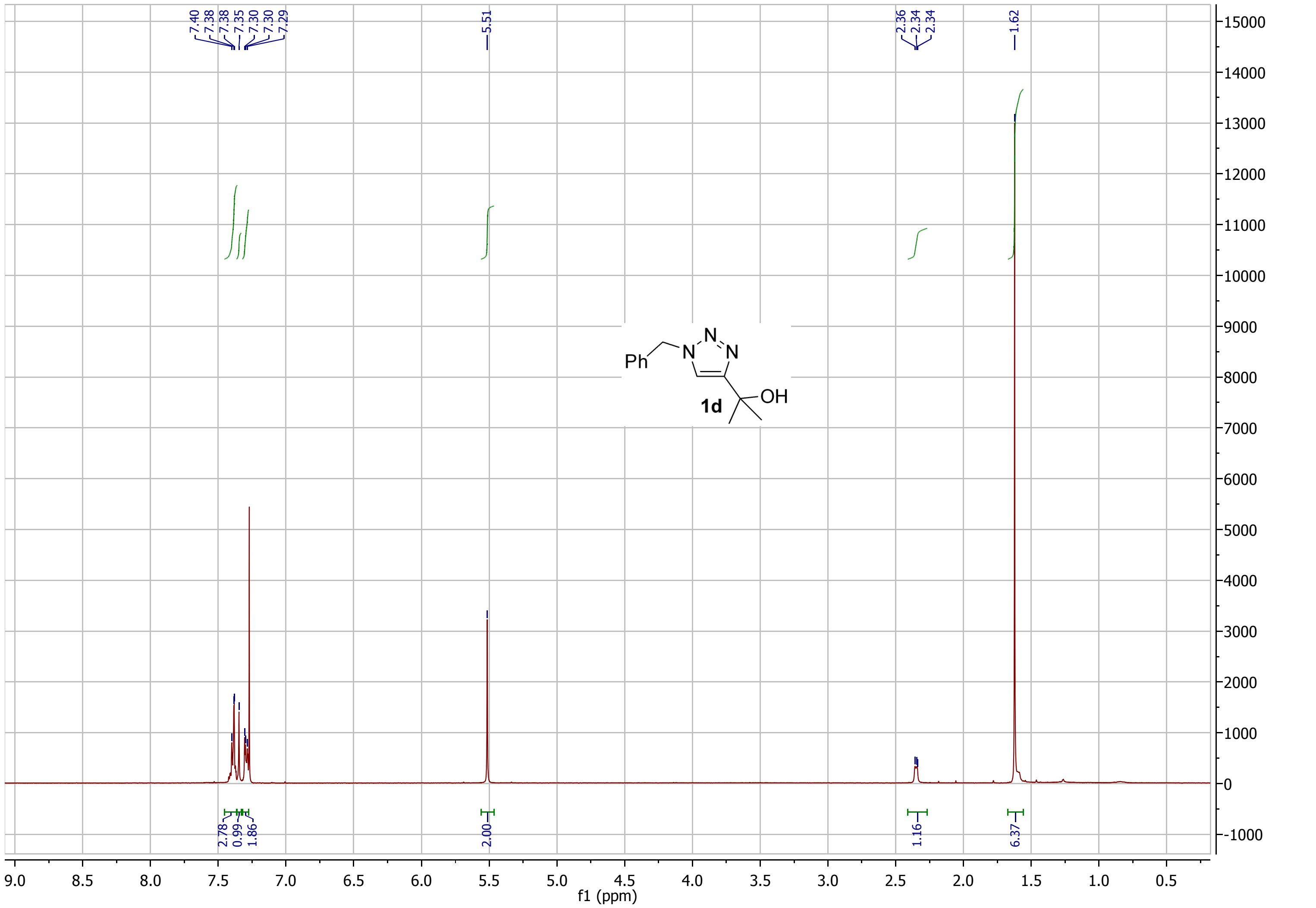
7.40
7.38
7.38
7.35
7.30
7.30
7.29
5.51
2.36
2.34
2.34
1.62
Ph
N
N
N
OH
1d
2.78
0.99
1.86
2.00
1.16
6.37
f1 (ppm)

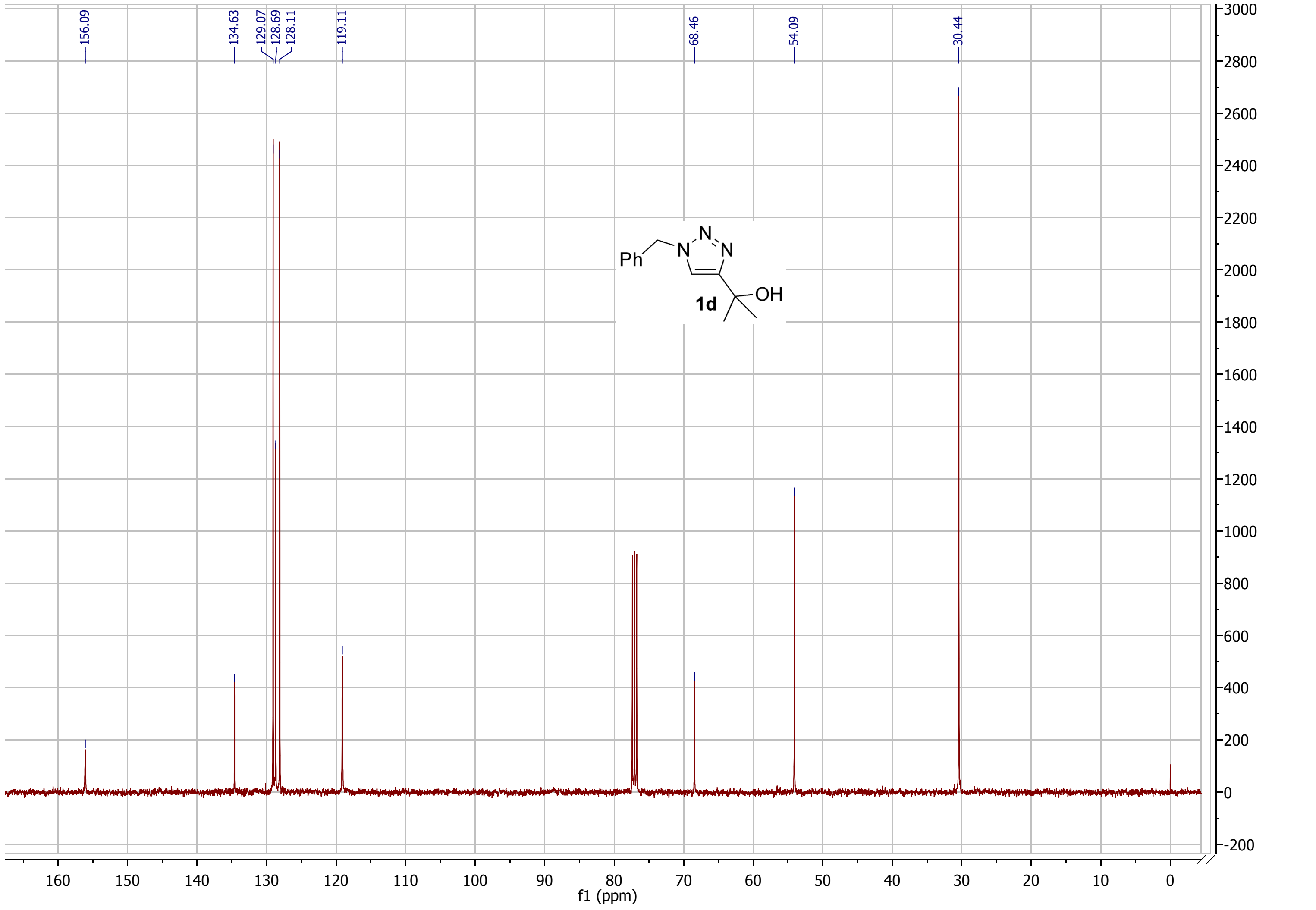
156.09
134.63
129.07
128.69
128.11
119.11
68.46
54.09
30.44
Ph
N
N
N
OH
1d
f1 (ppm)

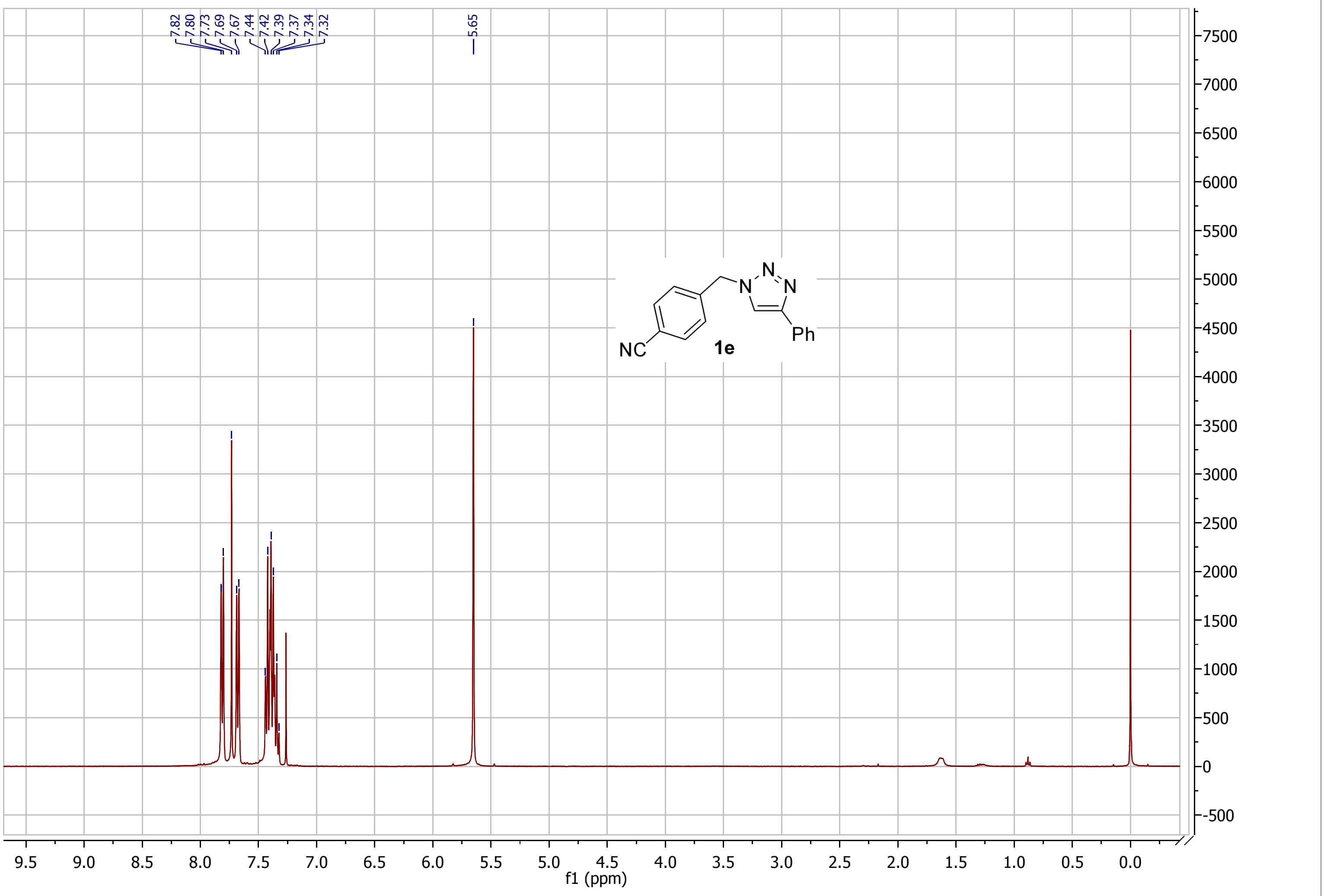
7.82
7.80
7.73
7.69
7.67
7.44
7.42
7.39
7.37
7.34
7.32
5.65
NC
1e
Ph
f1 (ppm)

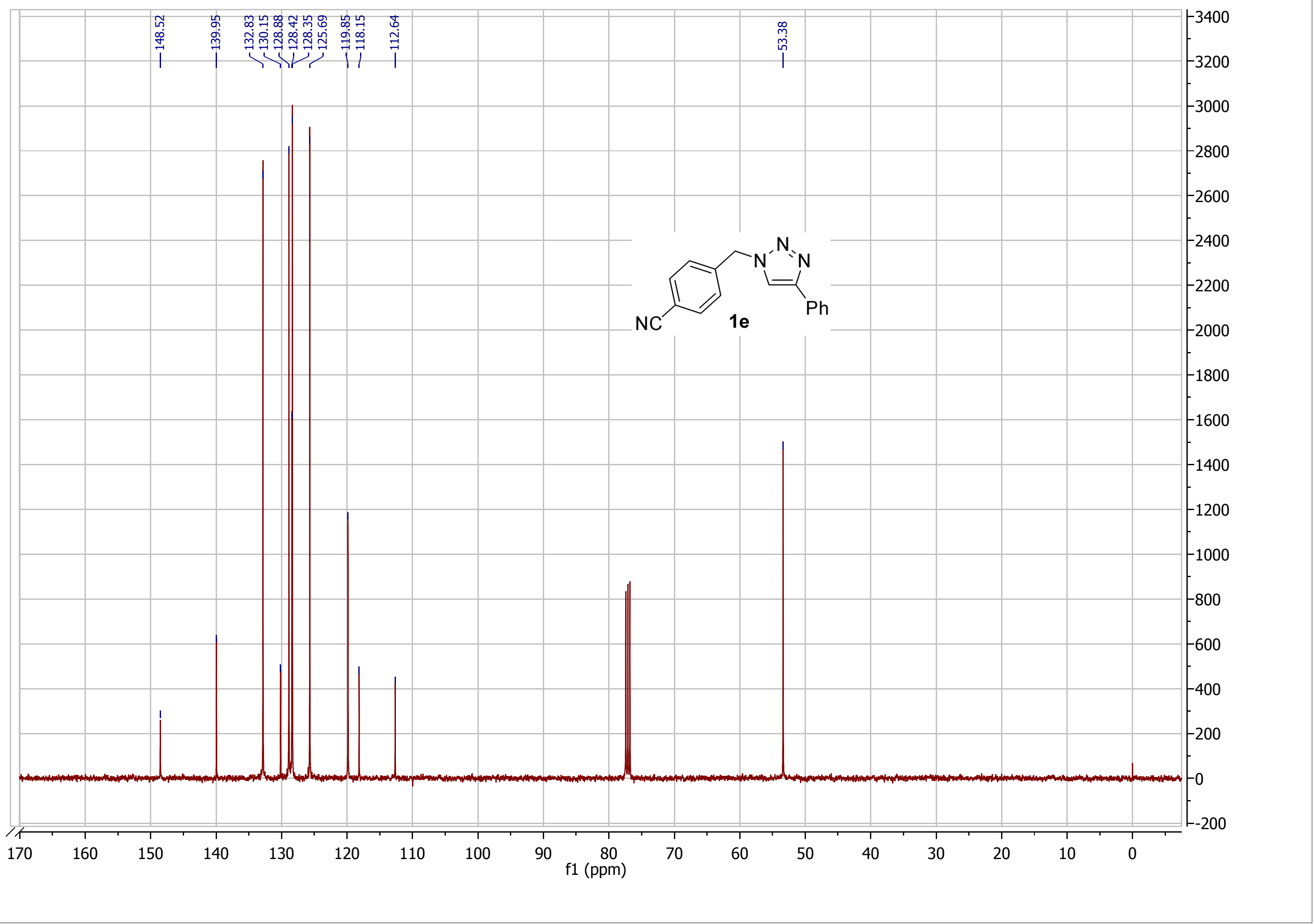
148.52
139.95
132.83
130.15
128.88
128.42
128.35
125.69
119.85
118.15
112.64
53.38
NC
1e
Ph
f1 (ppm)

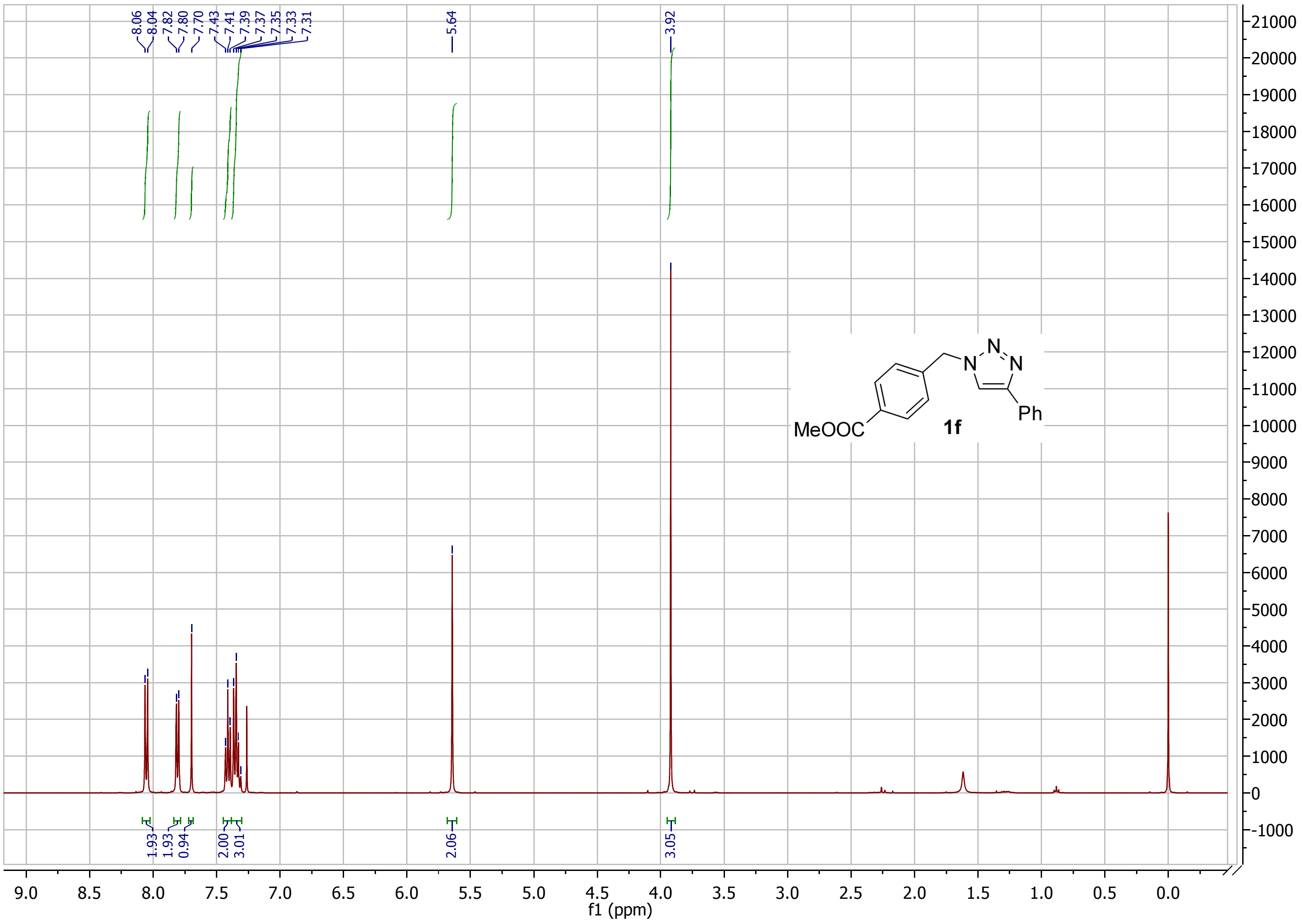
8.06
8.04
7.82
7.80
7.70
7.43
7.41
7.39
7.37
7.35
7.33
7.31
5.64
3.92
MeOOC
1f
Ph
1.93
1.93
0.94
2.00
3.01
2.06
3.05
f1 (ppm)

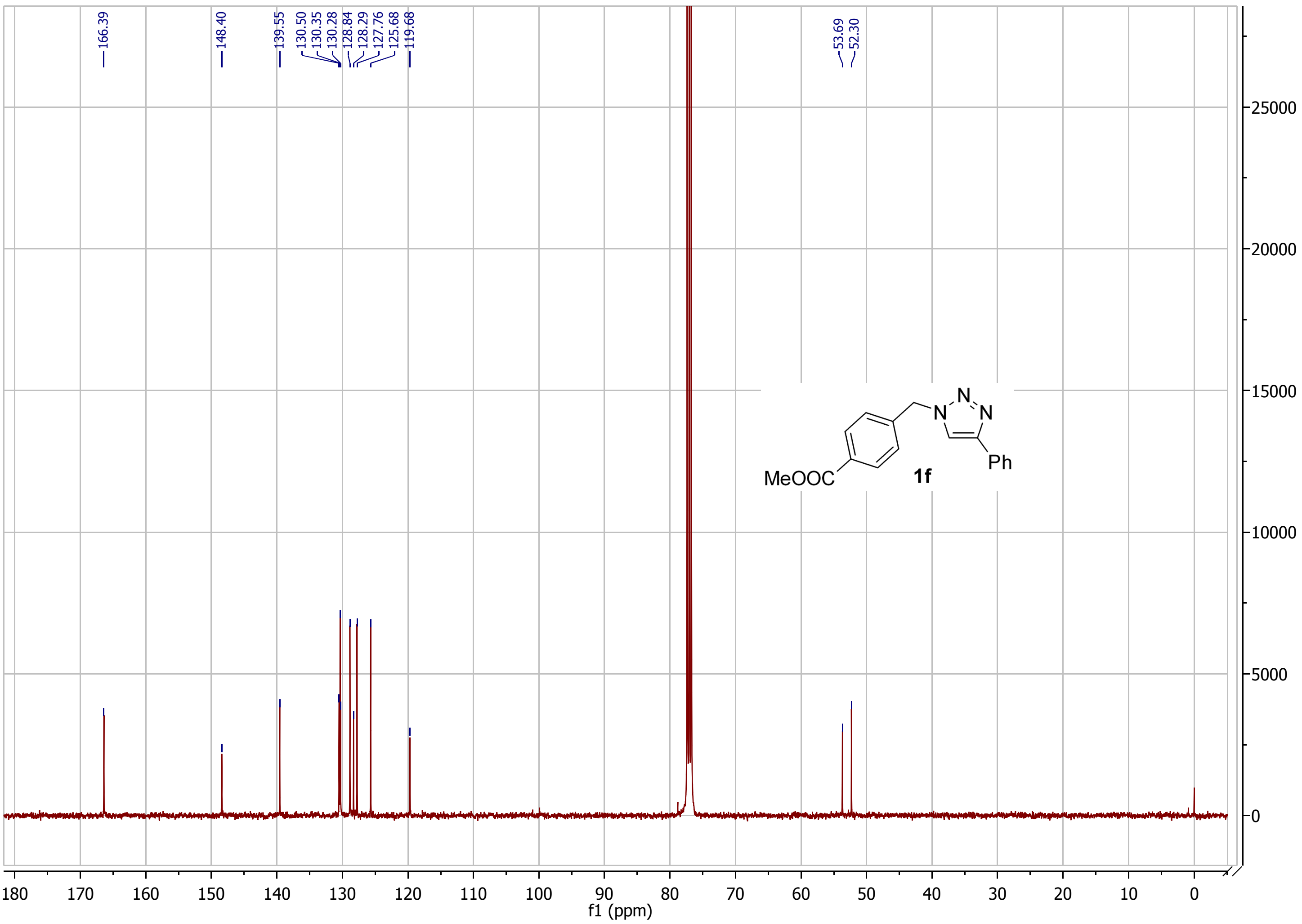
166.39
148.40
139.55
130.50
130.35
130.28
128.84
128.29
127.76
125.68
119.68
53.69
52.30
MeOOC
1f
Ph
f1 (ppm)

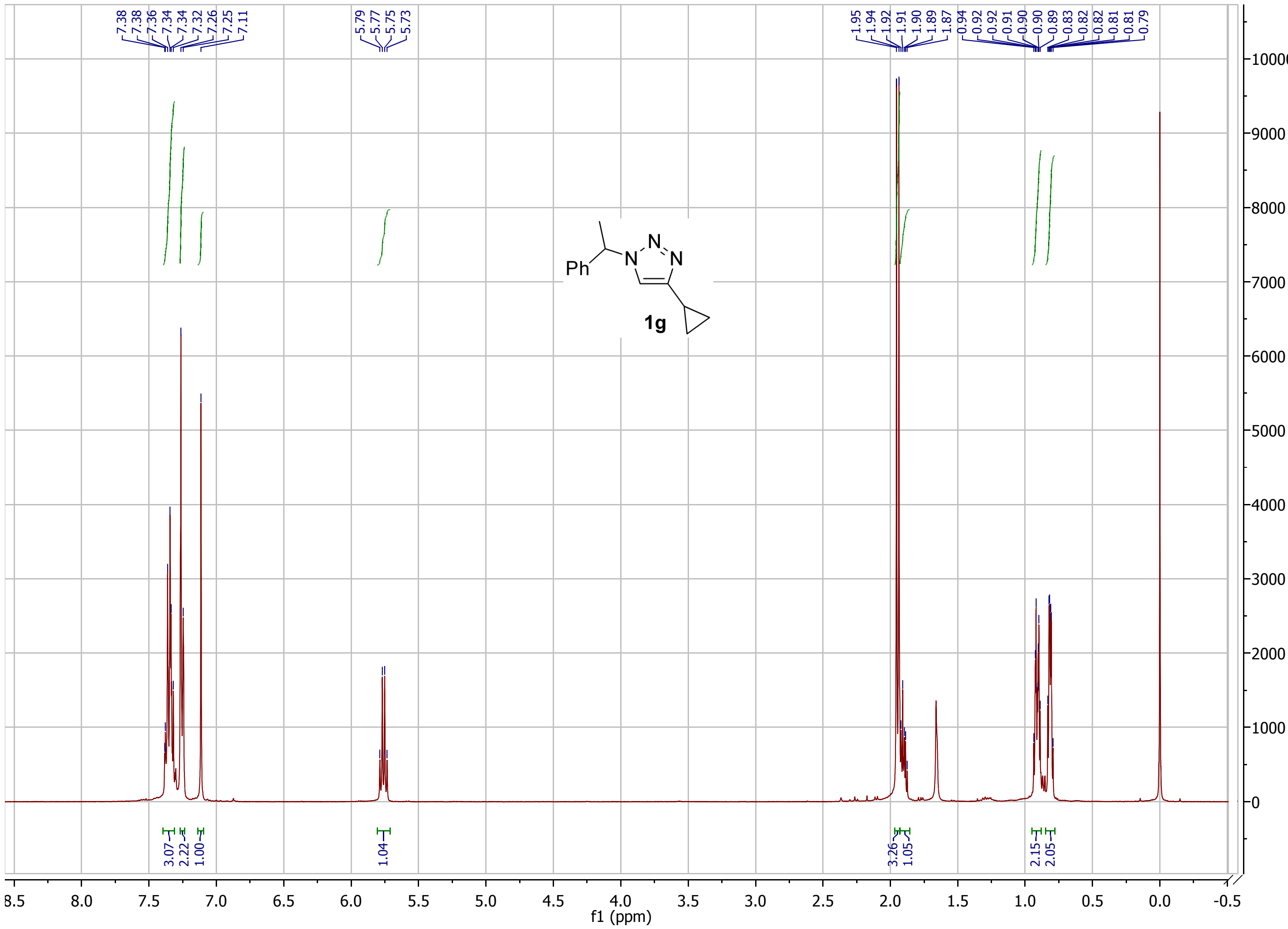
7.38 7.38 7.36 7.34 7.34 7.32 7.26 7.25 7.11
5.79 5.77 5.75 5.73
1.95 1.94 1.92 1.91 1.90 1.89 1.87 0.94 0.92 0.92 0.91 0.90 0.90 0.89 0.83 0.82 0.82 0.81 0.81 0.79
Ph
N
N
N
1g
3.07 2.22 1.00 1.04 3.26 1.05 2.15 2.05
f1 (ppm)

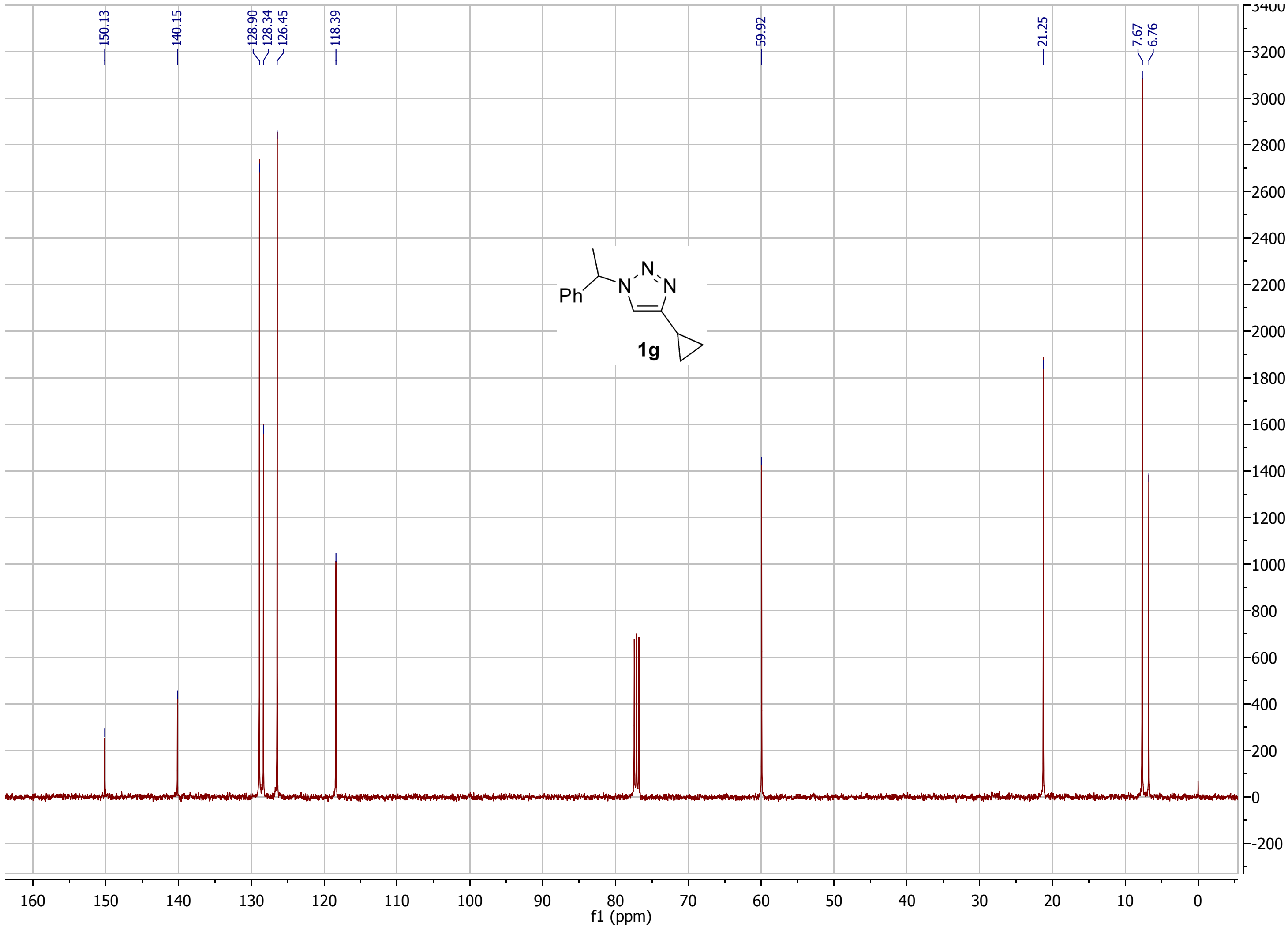
150.13 140.15 128.90 128.34 126.45 118.39 59.92 21.25 7.67 6.76
Ph
N
N
N
1g
f1 (ppm)

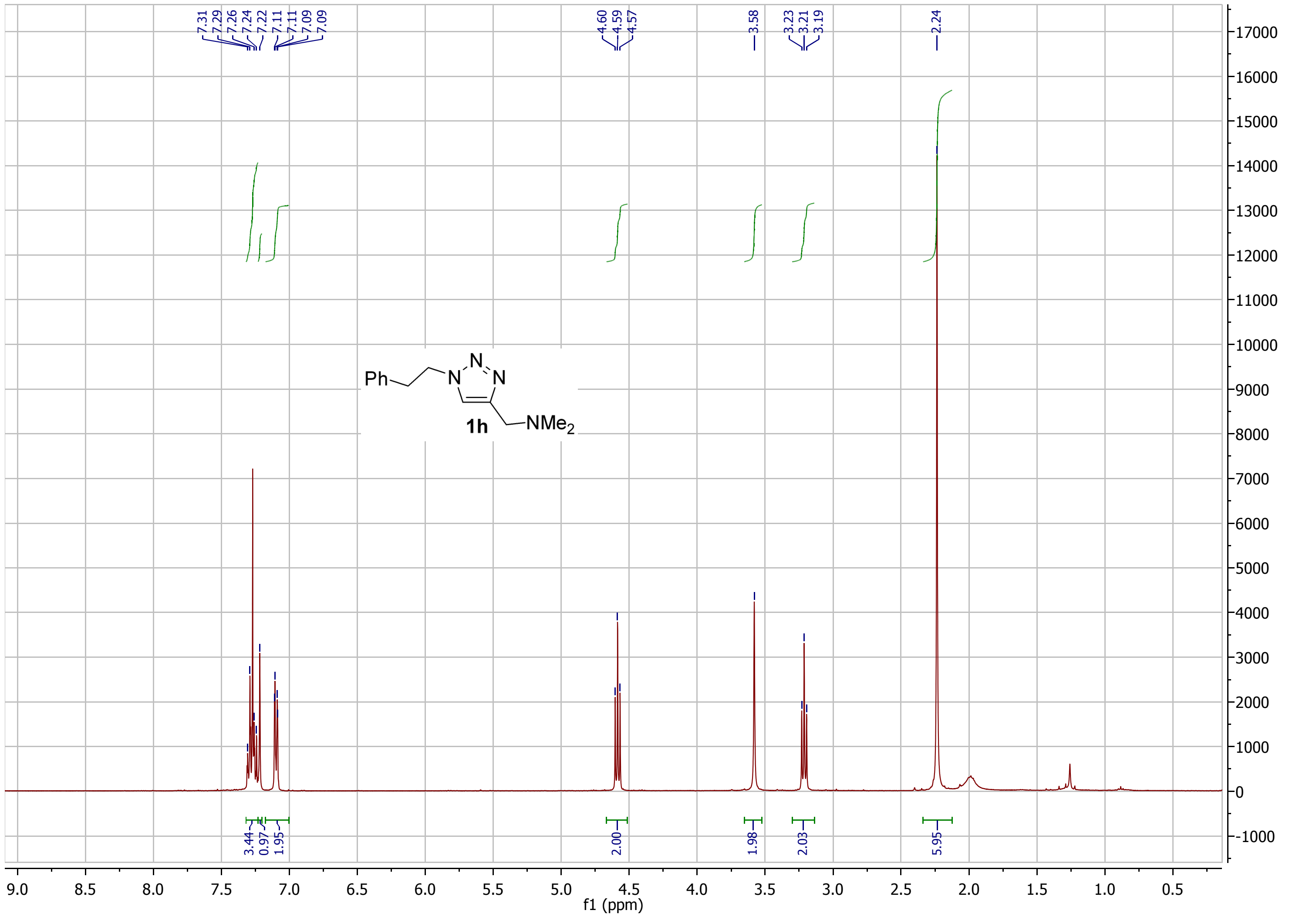
7.31
7.29
7.26
7.24
7.22
7.11
7.11
7.09
7.09
4.60
4.59
4.57
3.58
3.23
3.21
3.19
2.24
Ph
N
N
N
1h
NMe2
3.44
0.97
1.95
2.00
1.98
2.03
5.95
f1 (ppm)

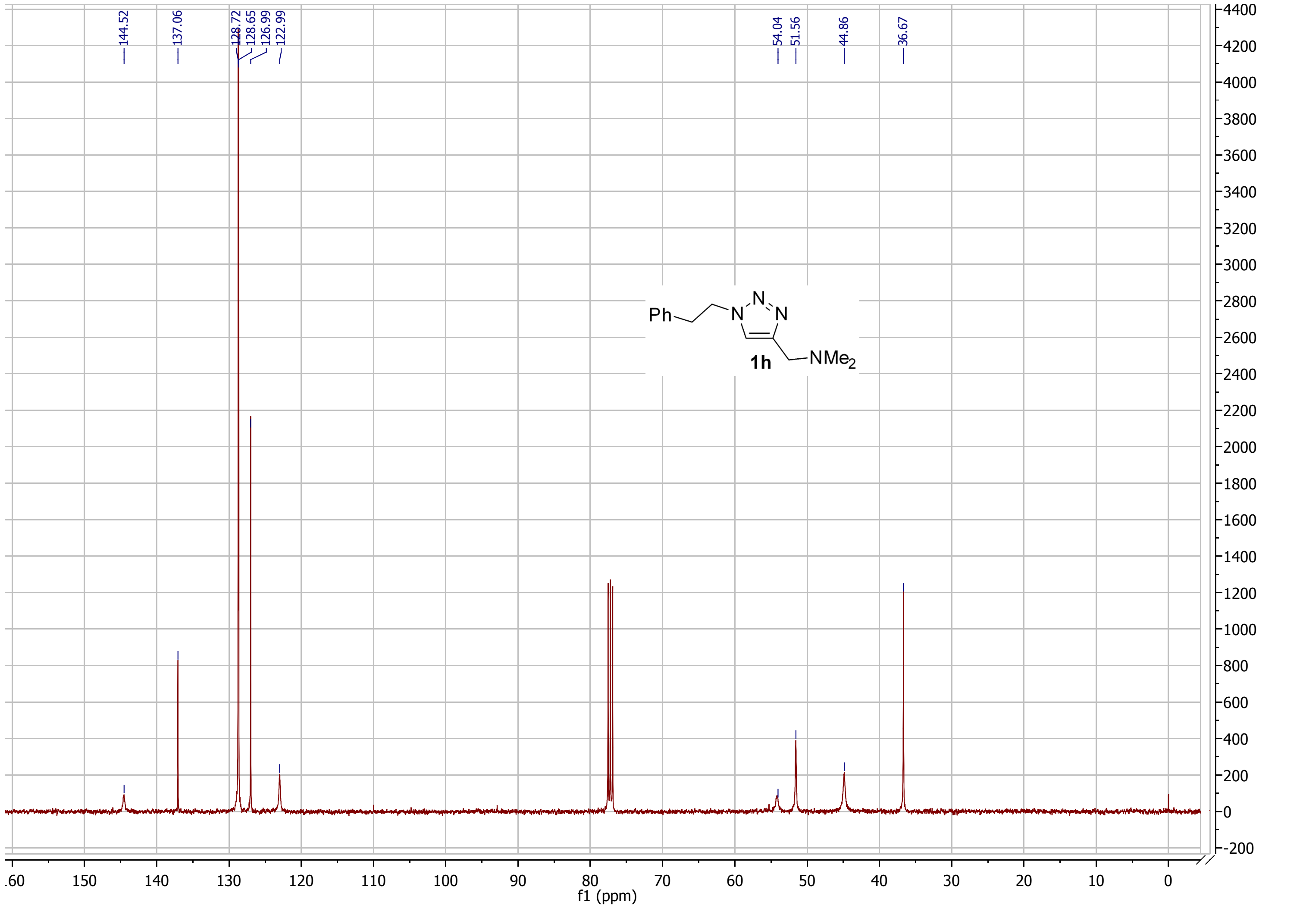
144.52
137.06
128.72
128.65
126.99
122.99
54.04
51.56
44.86
36.67
Ph
N
N
N
1h
NMe2
f1 (ppm)

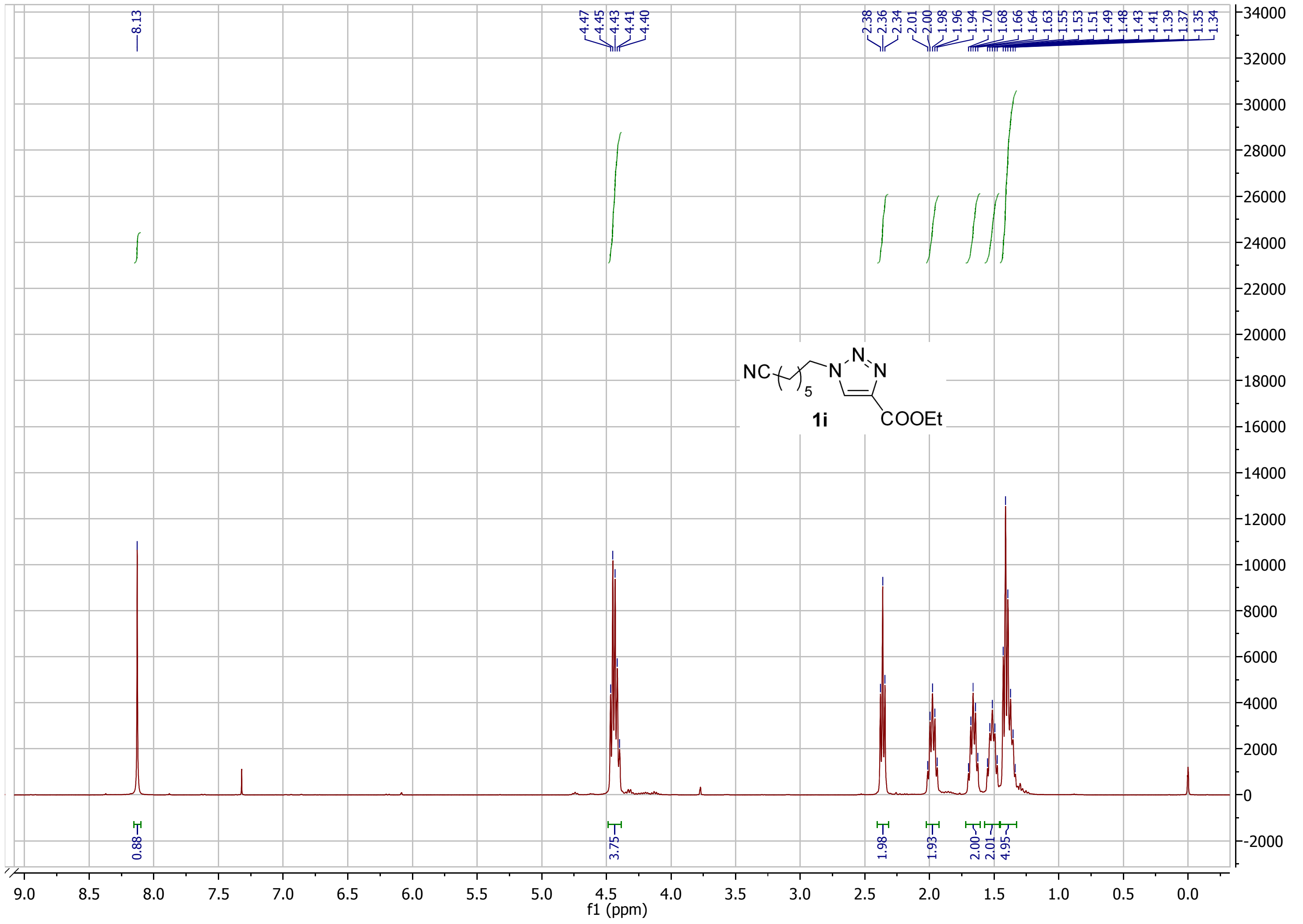
8.13
4.47
4.45
4.43
4.41
4.40
2.38
2.36
2.34
2.01
2.00
1.98
1.96
1.94
1.70
1.68
1.66
1.64
1.63
1.55
1.53
1.51
1.49
1.48
1.43
1.41
1.39
1.37
1.35
1.34
NC
5
N
N
N
1i
COOEt
0.88
3.75
1.98
1.93
2.00
2.01
4.95
f1 (ppm)

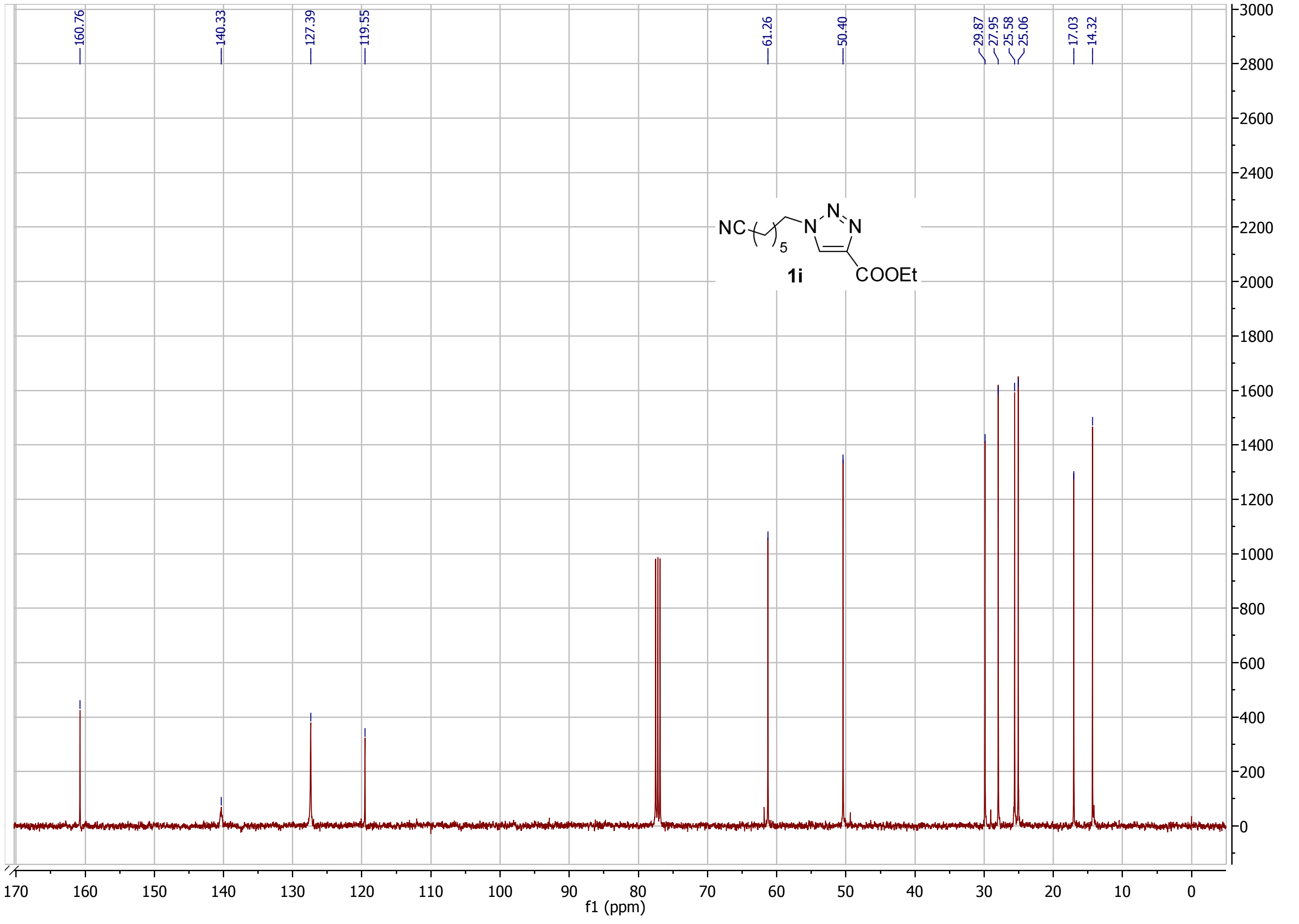
160.76
140.33
127.39
119.55
61.26
50.40
29.87
27.95
25.58
25.06
17.03
14.32
NC
5
N
N
N
1i
COOEt
f1 (ppm)

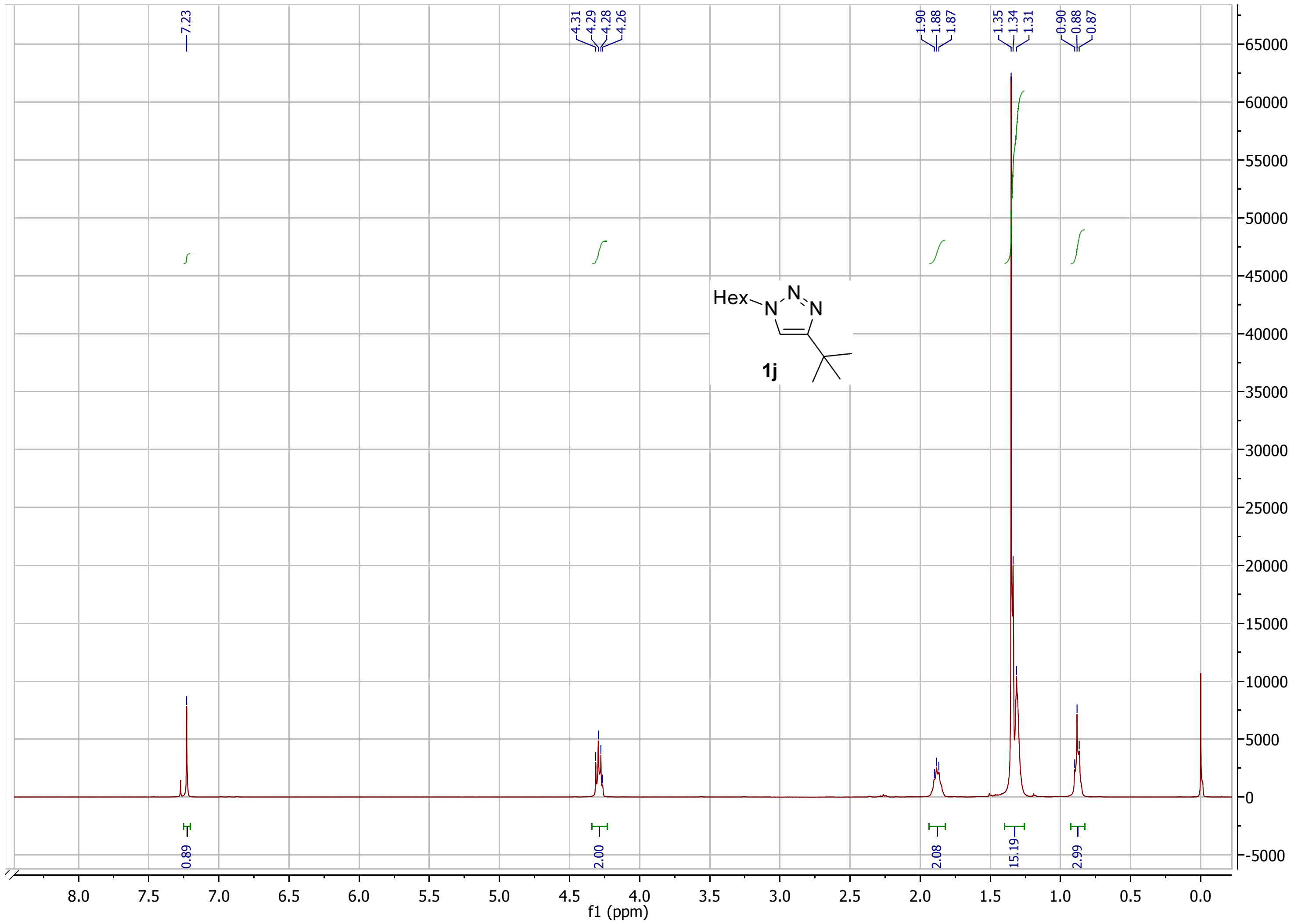

7.23
4.31
4.29
4.28
4.26
1.90
1.88
1.87
1.35
1.34
1.31
0.90
0.88
0.87
Hex
N
N
N
1j
0.89
2.00
2.08
15.19
2.99
f1 (ppm)

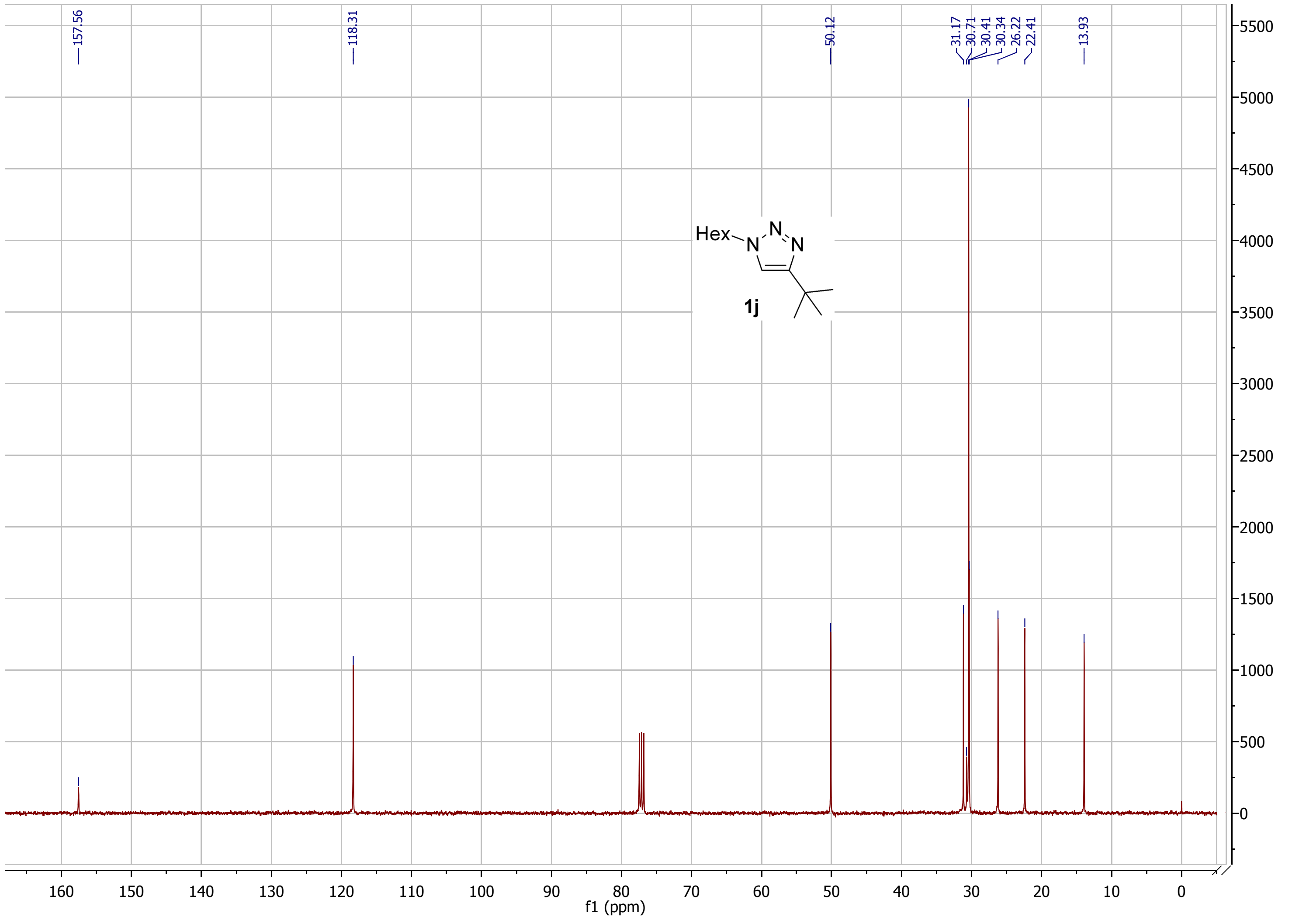

157.56
118.31
50.12
31.17
30.71
30.41
30.34
26.22
22.41
13.93
Hex
N
N
N
1j
f1 (ppm)

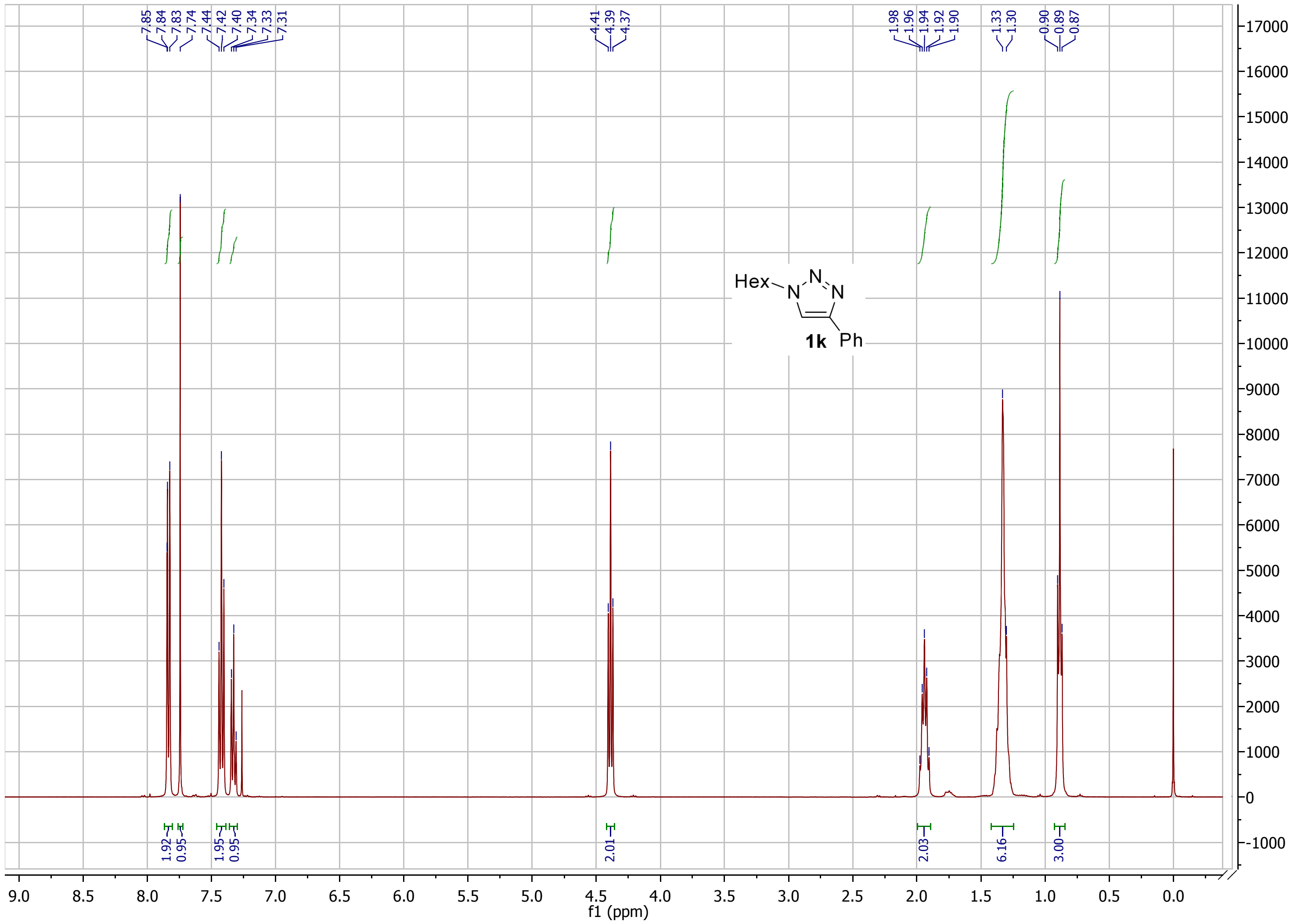

7.85
7.84
7.83
7.74
7.44
7.42
7.40
7.34
7.33
7.31
4.41
4.39
4.37
1.98
1.96
1.94
1.92
1.90
1.33
1.30
0.90
0.89
0.87
Hex
N
N
N
1k
Ph
1.92
0.95
1.95
0.95
2.01
2.03
6.16
3.00
f1 (ppm)

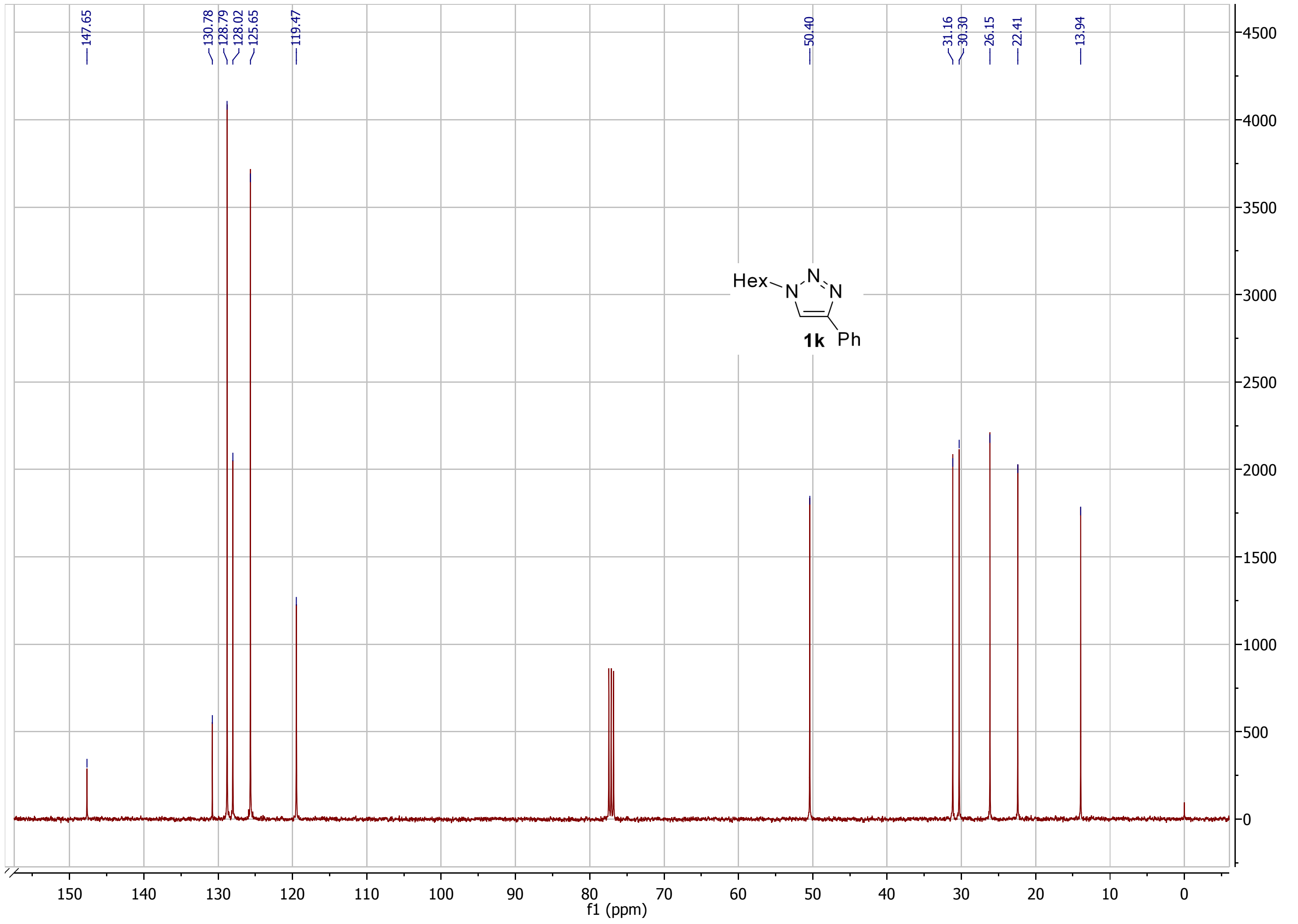

147.65
130.78
128.79
128.02
125.65
119.47
50.40
31.16
30.30
26.15
22.41
13.94
Hex
N
N
N
1k
Ph
f1 (ppm)

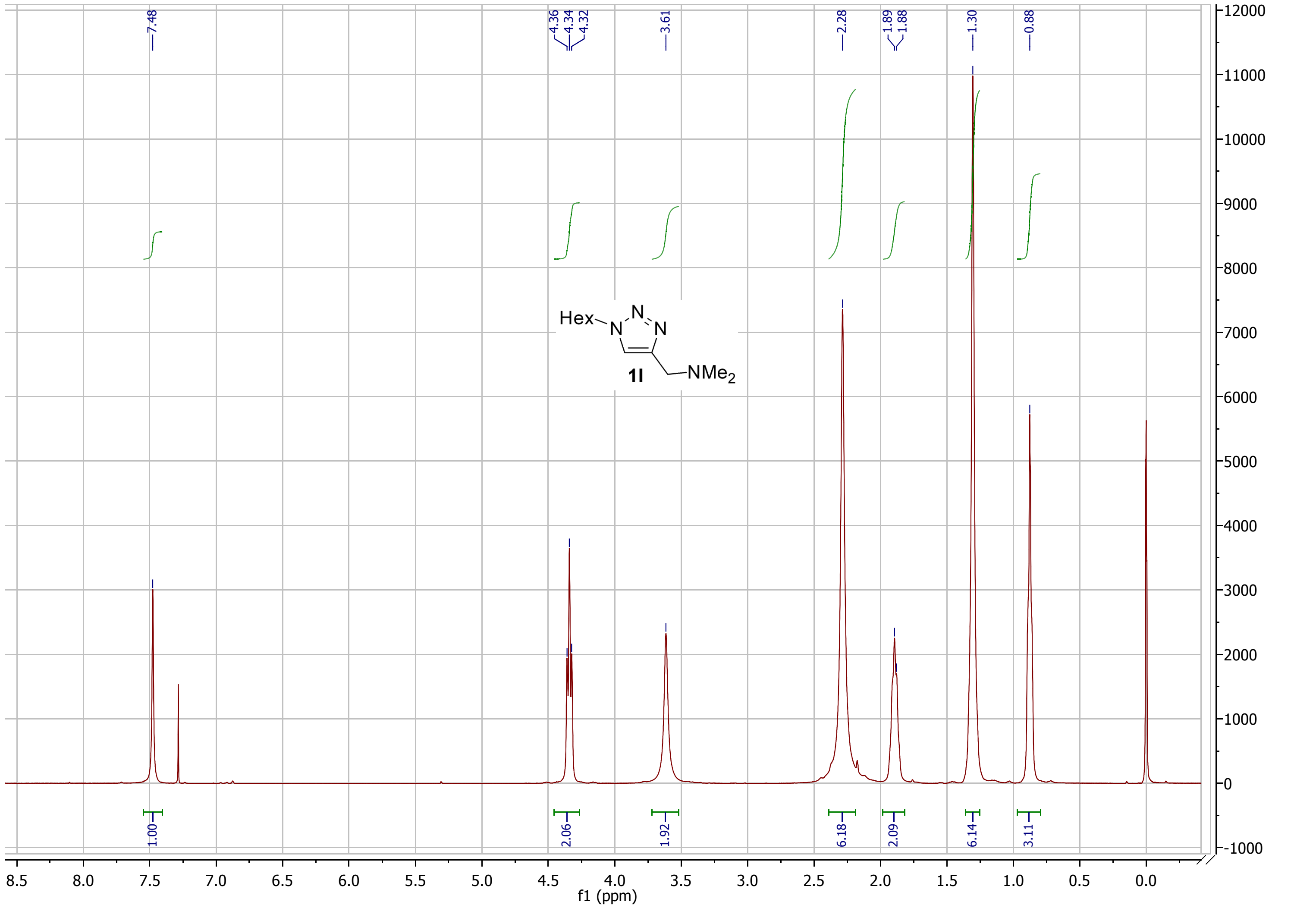

7.48
4.36
4.34
4.32
3.61
2.28
1.89
1.88
1.30
0.88
Hex
N
N
N
1l
NMe2
1.00
2.06
1.92
6.18
2.09
6.14
3.11
f1 (ppm)

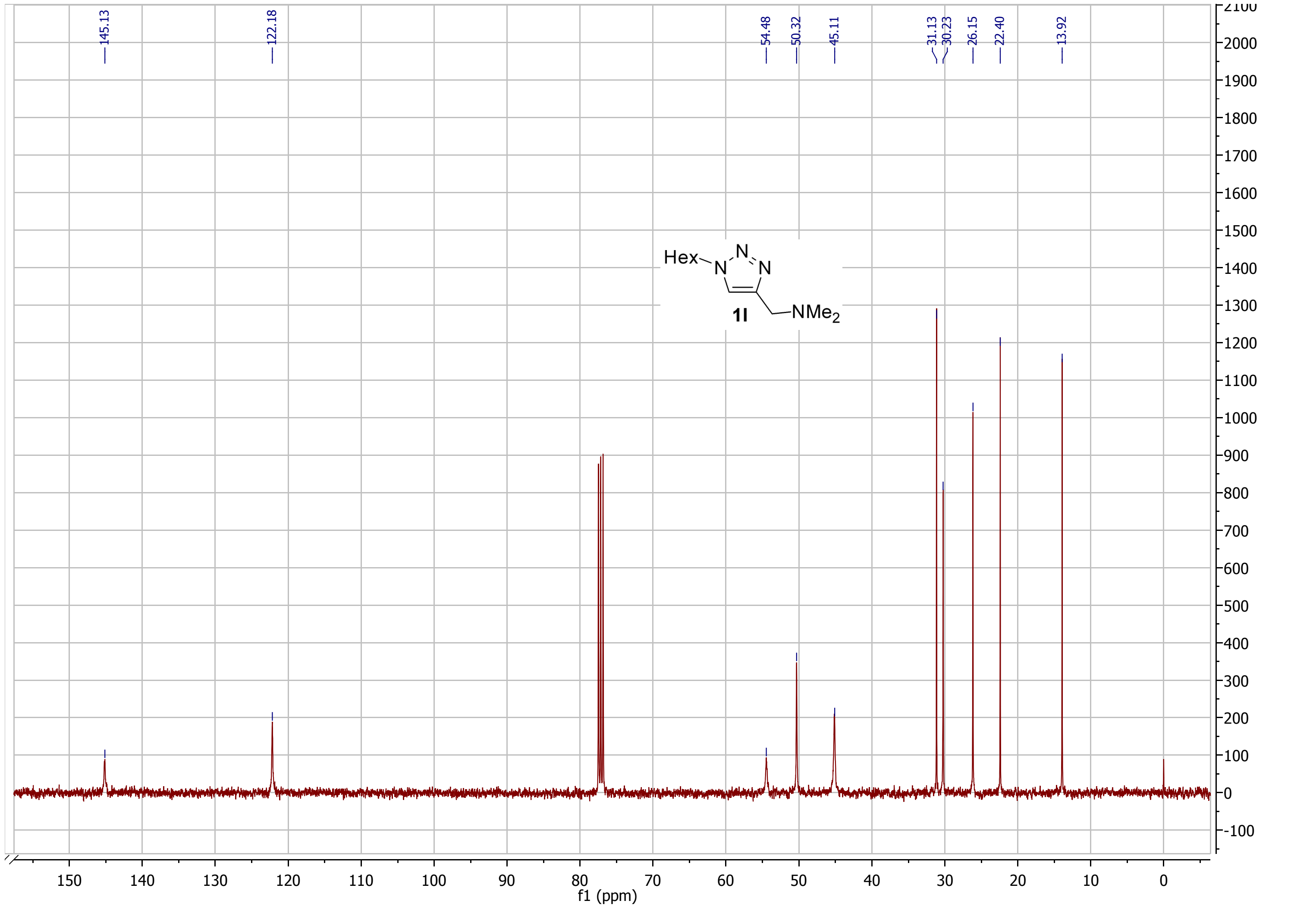

145.13
122.18
54.48
50.32
45.11
31.13
30.23
26.15
22.40
13.92
Hex
N
N
N
1l
NMe2
f1 (ppm)

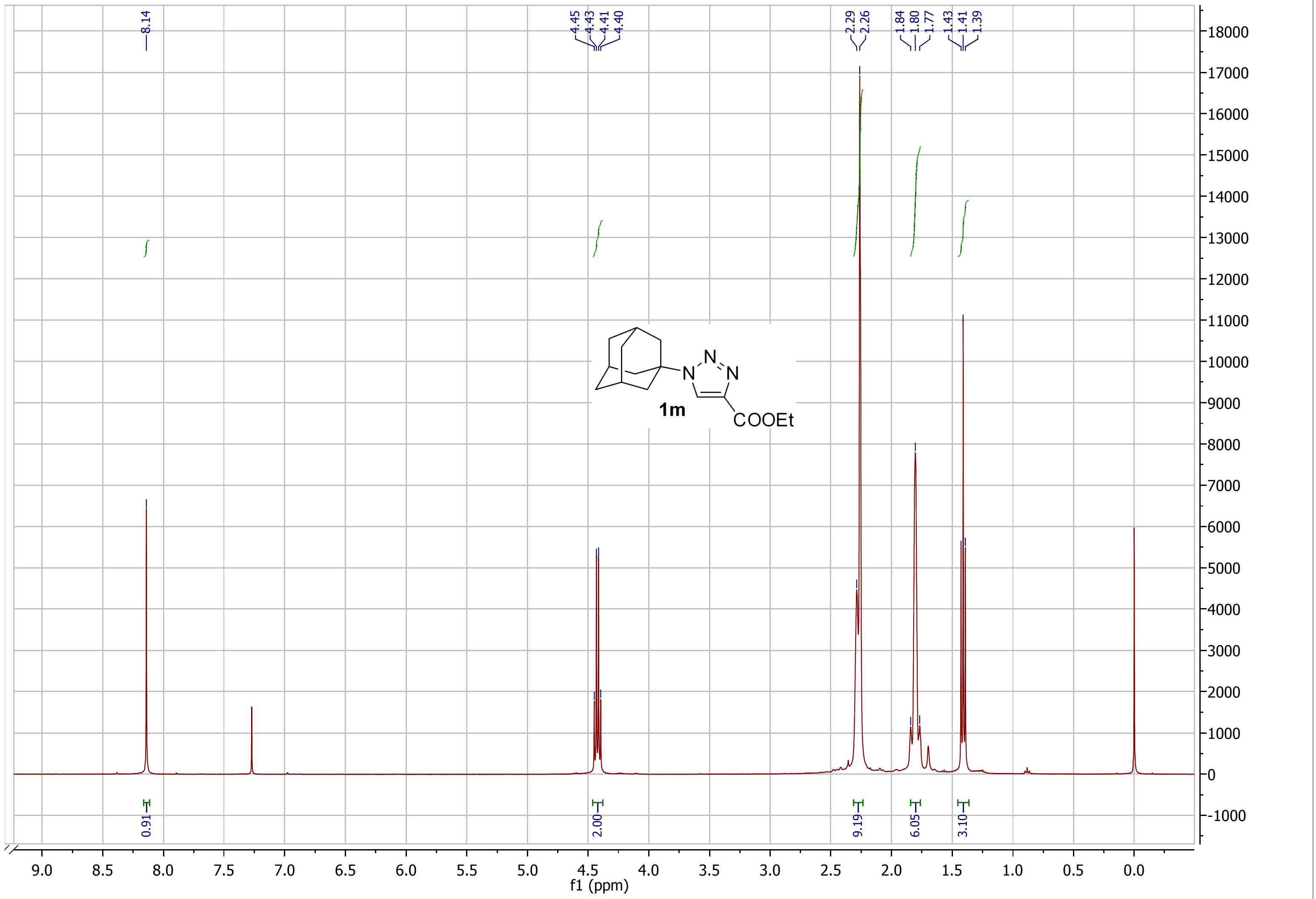

8.14
4.45
4.43
4.41
4.40
2.29
2.26
1.84
1.80
1.77
1.43
1.41
1.39
1m
COOEt
0.91
2.00
9.19
6.05
3.10
f1 (ppm)

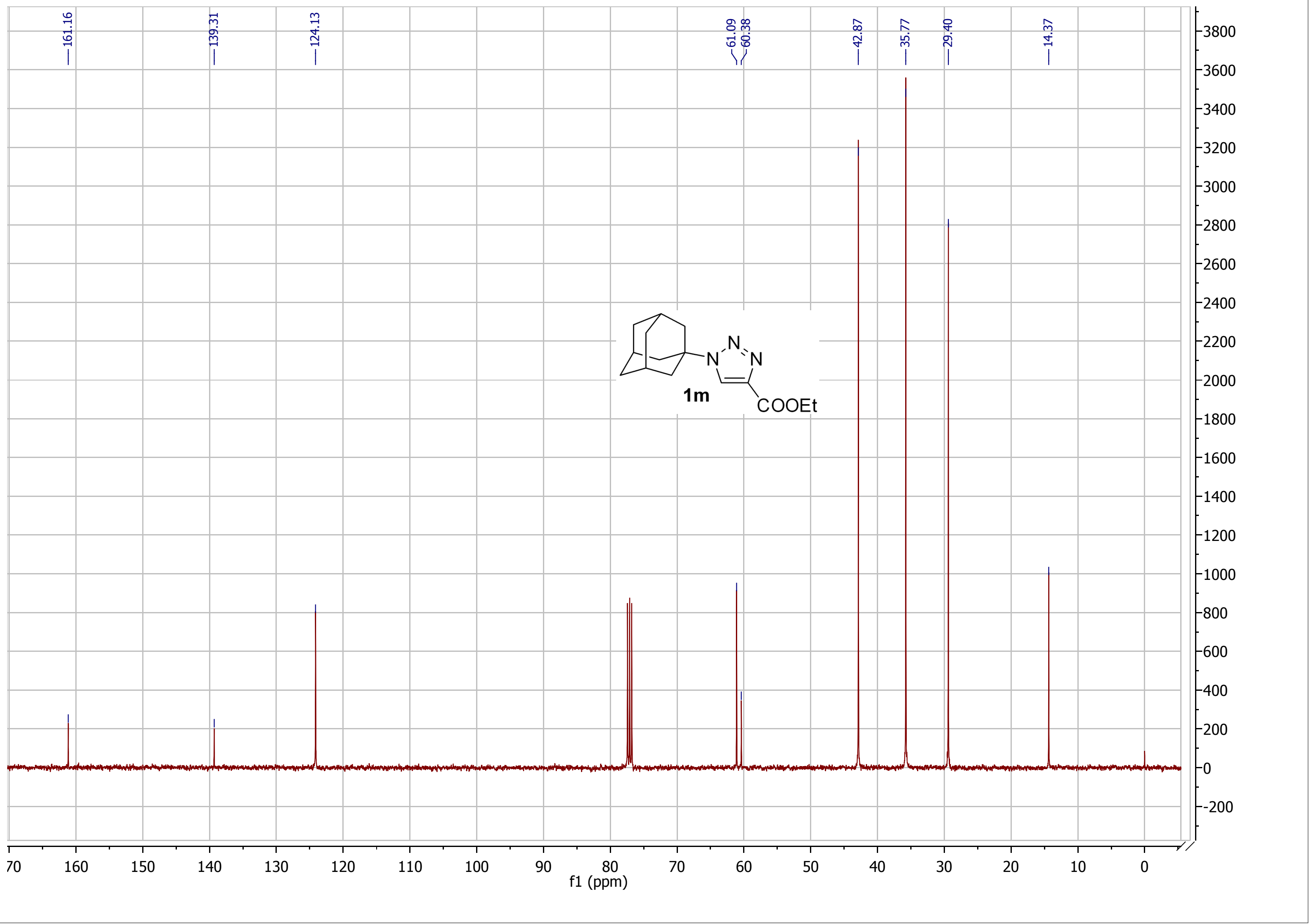

161.16
139.31
124.13
61.09
60.38
42.87
35.77
29.40
14.37
1m
COOEt
f1 (ppm)

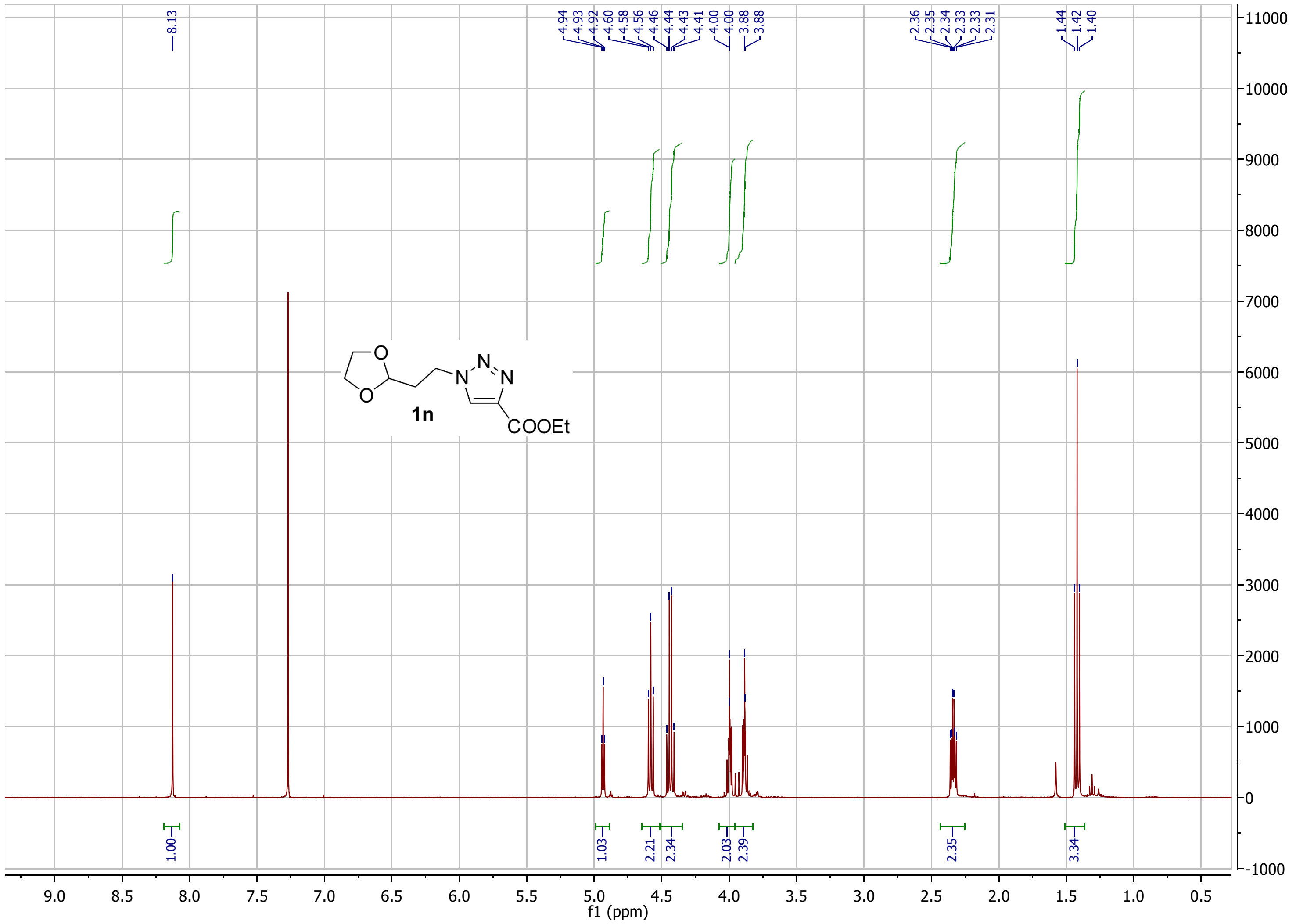
8.13
4.94
4.93
4.92
4.60
4.58
4.56
4.46
4.44
4.43
4.41
4.00
4.00
3.88
3.88
2.36
2.35
2.34
2.33
2.33
2.31
1.44
1.42
1.40
1n
COOEt
1.00
1.03
2.21
2.34
2.03
2.39
2.35
3.34
f1 (ppm)

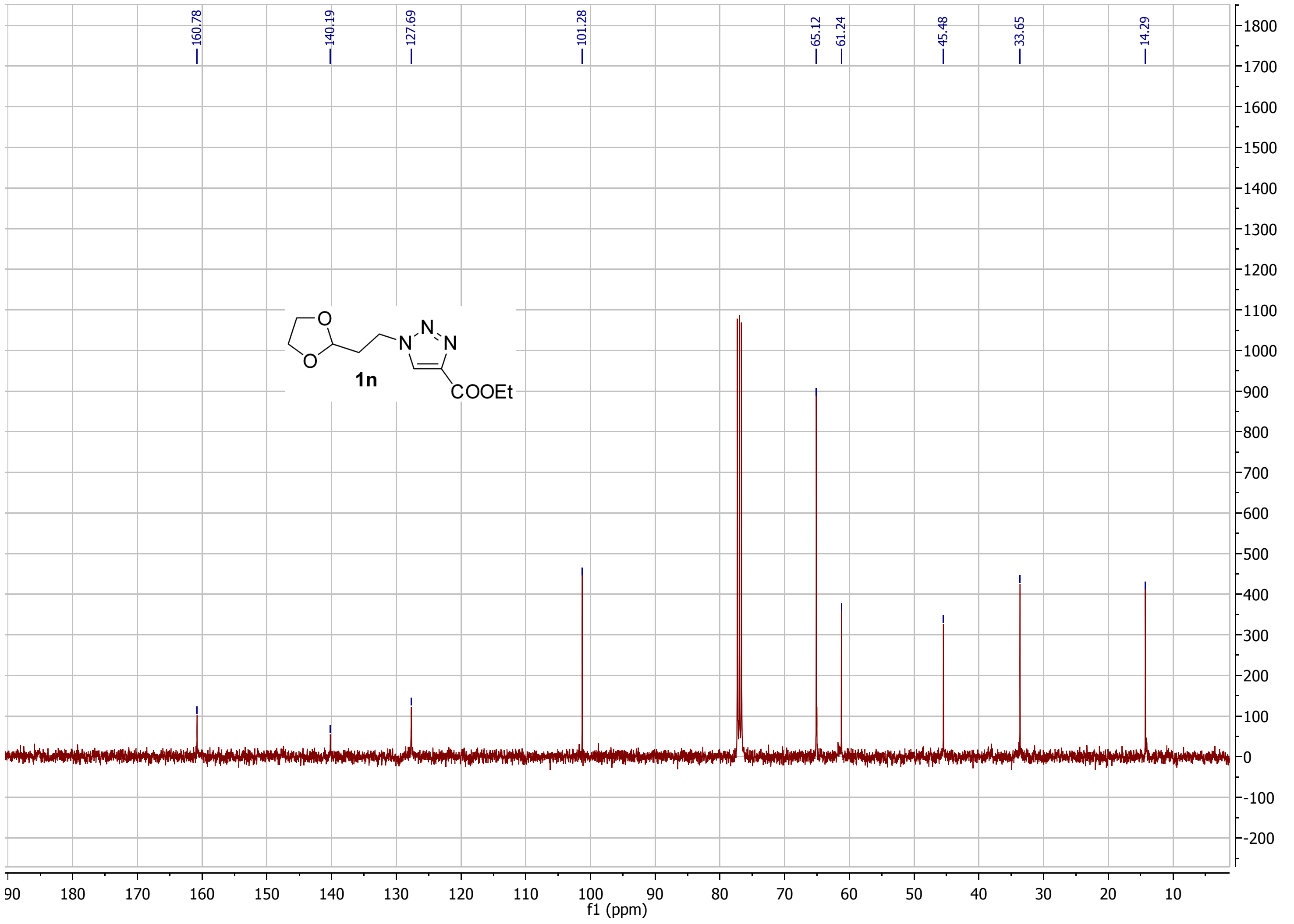
160.78
140.19
127.69
101.28
65.12
61.24
45.48
33.65
14.29
1n
COOEt
f1 (ppm)

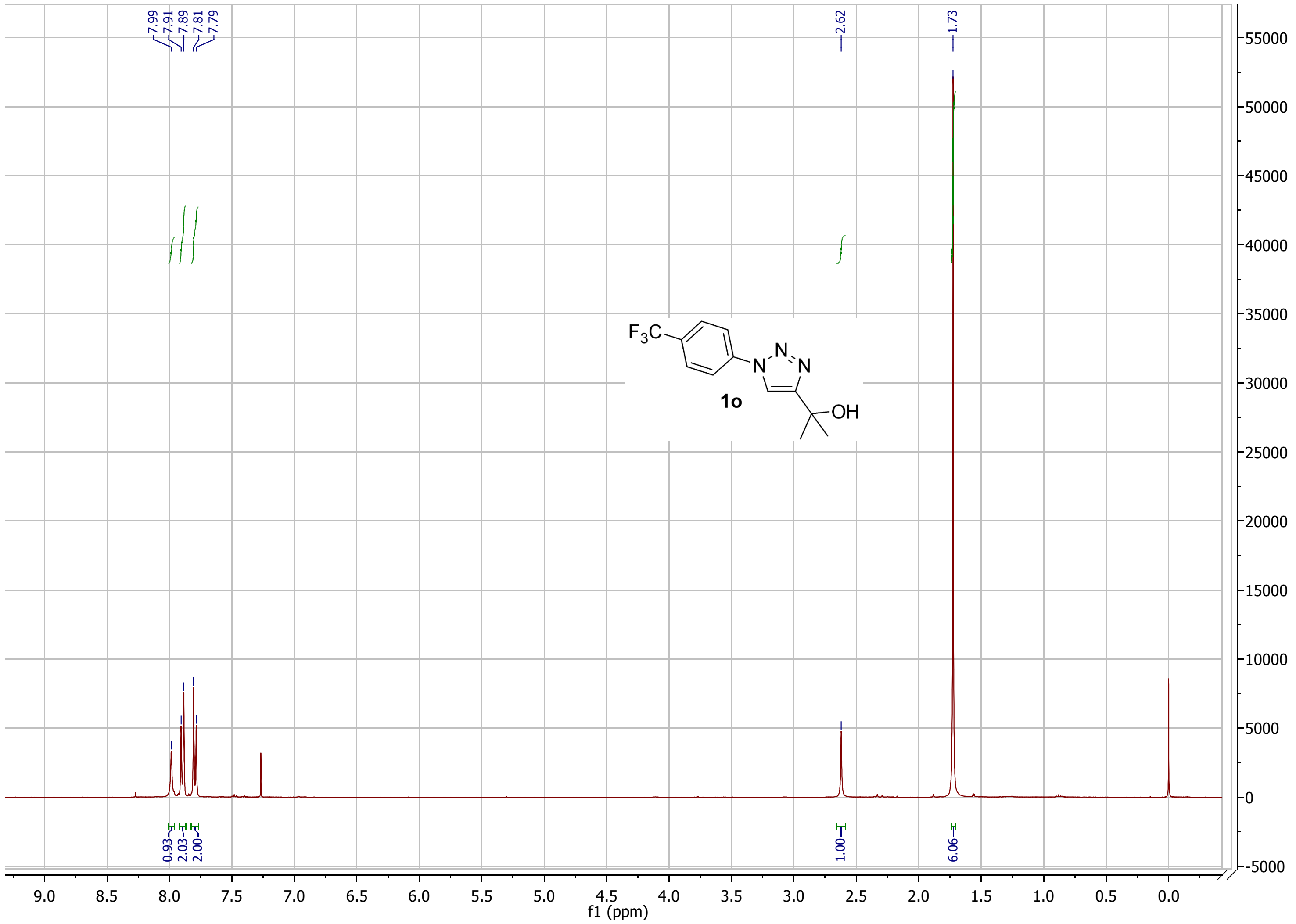

7.99
7.91
7.89
7.81
7.79
2.62
1.73
F3C
N
N
N
1o
OH
0.93
2.03
2.00
1.00
6.06
f1 (ppm)

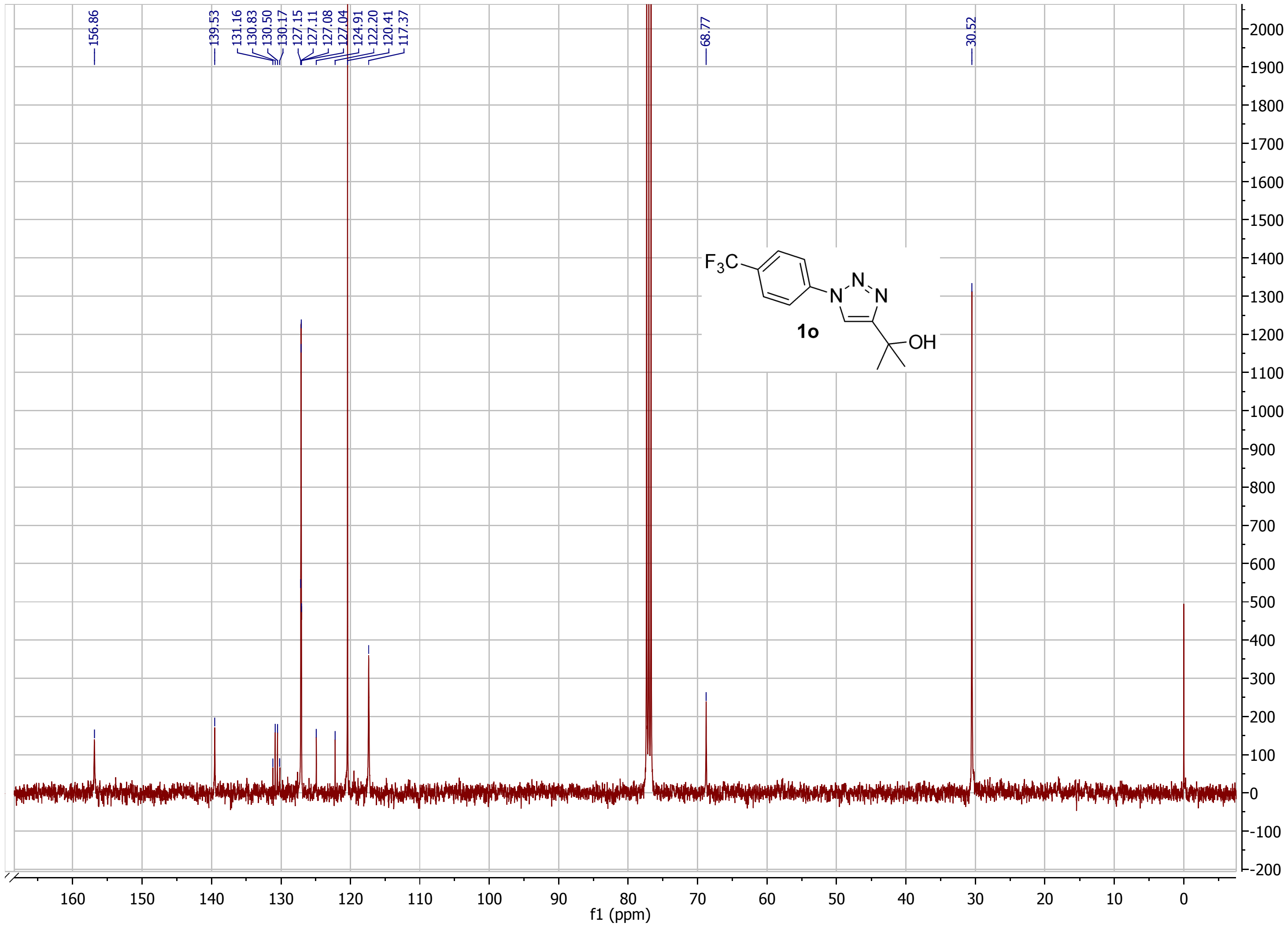

156.86
139.53
131.16
130.83
130.50
130.17
127.15
127.11
127.08
127.04
124.91
122.20
120.41
117.37
68.77
30.52
F3C
N
N
N
1o
OH
f1 (ppm)

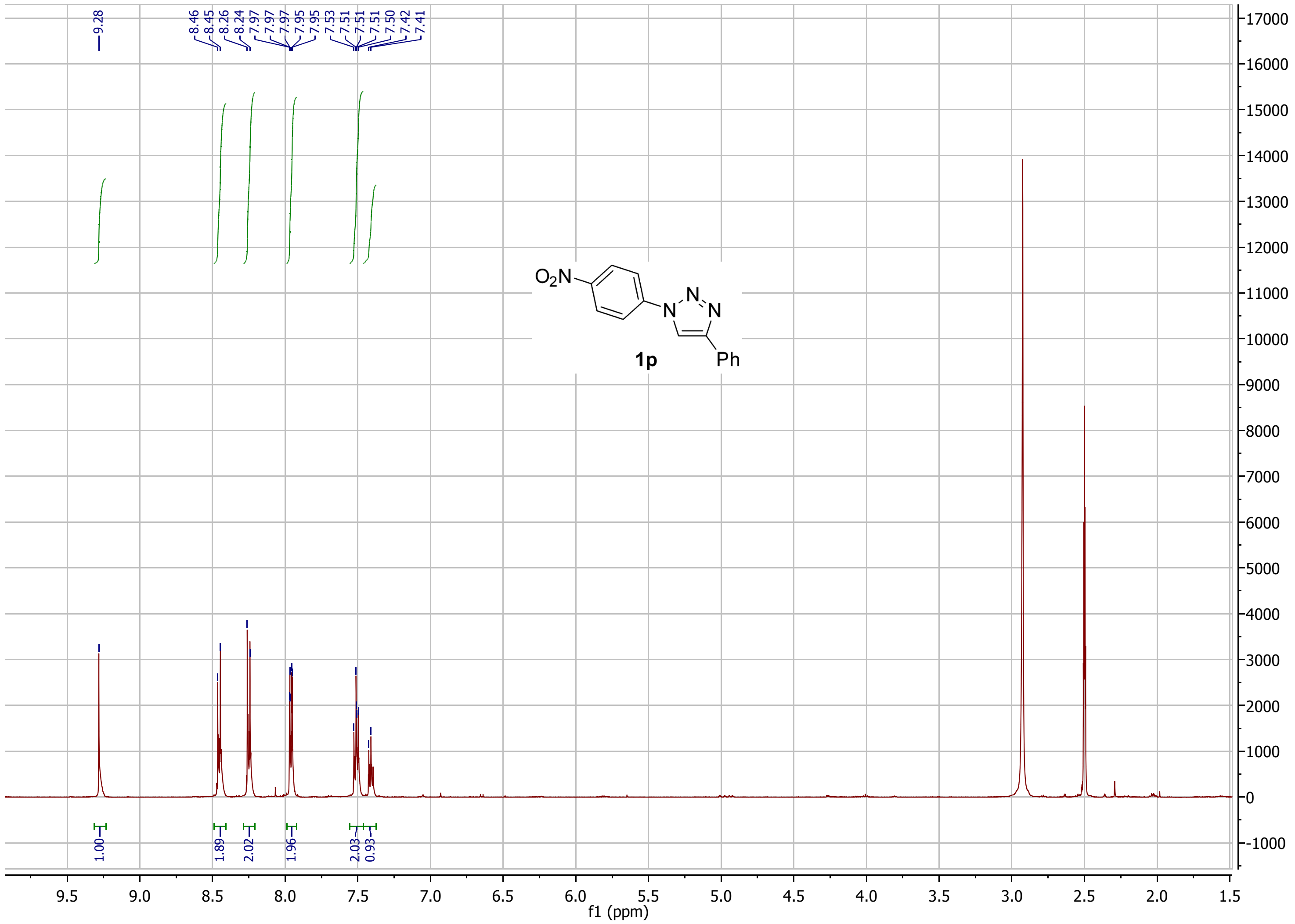

9.28
8.46
8.45
8.26
8.24
7.97
7.97
7.97
7.95
7.53
7.51
7.51
7.51
7.50
7.42
7.41
O_2N
N
N
N
Ph
1p
1.00
1.89
2.02
1.96
2.03
0.93
f1 (ppm)

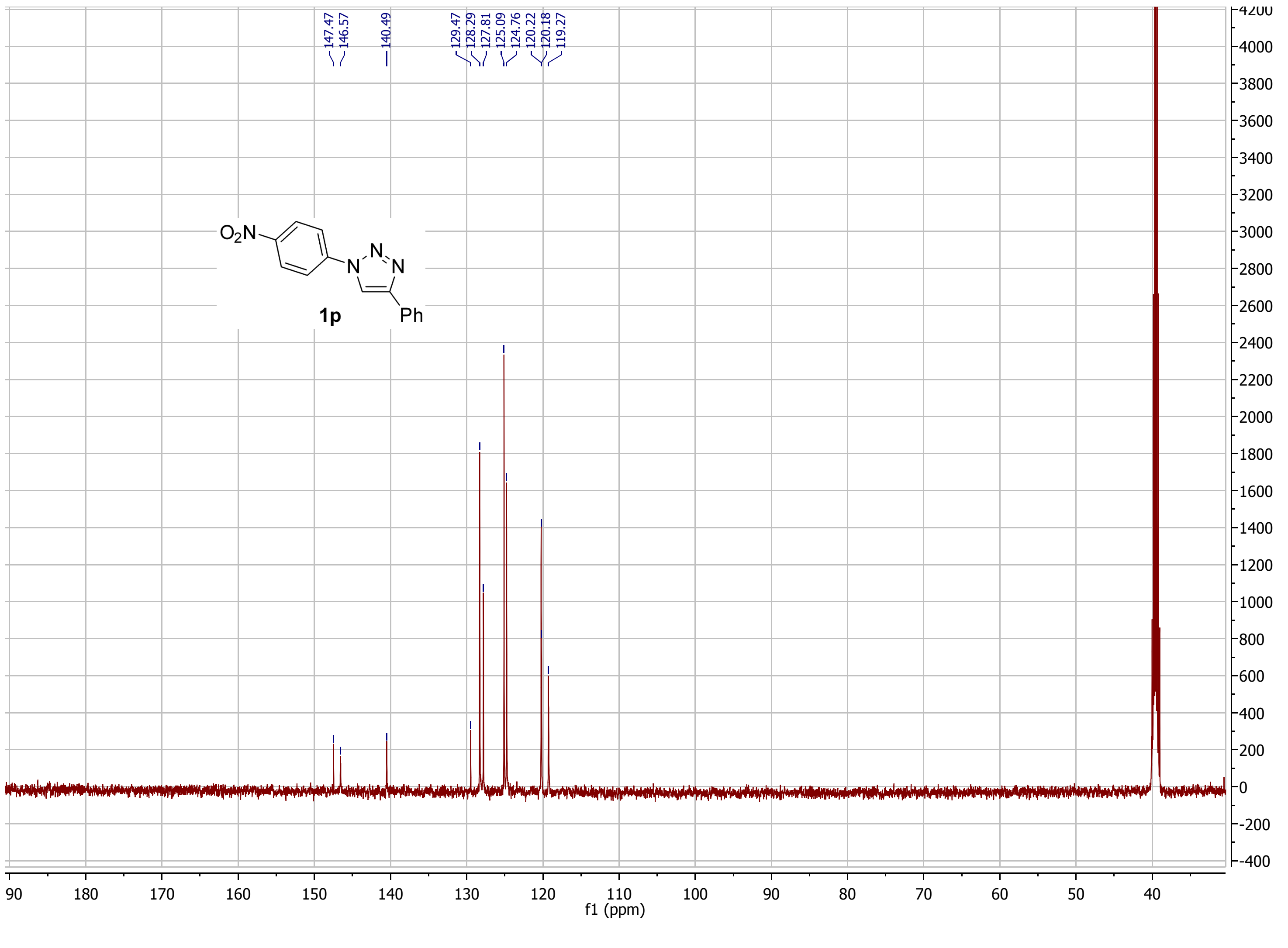

147.47
146.57
140.49
129.47
128.29
127.81
125.09
124.76
120.22
120.18
119.27
O_2N
N
N
N
Ph
1p
f1 (ppm)

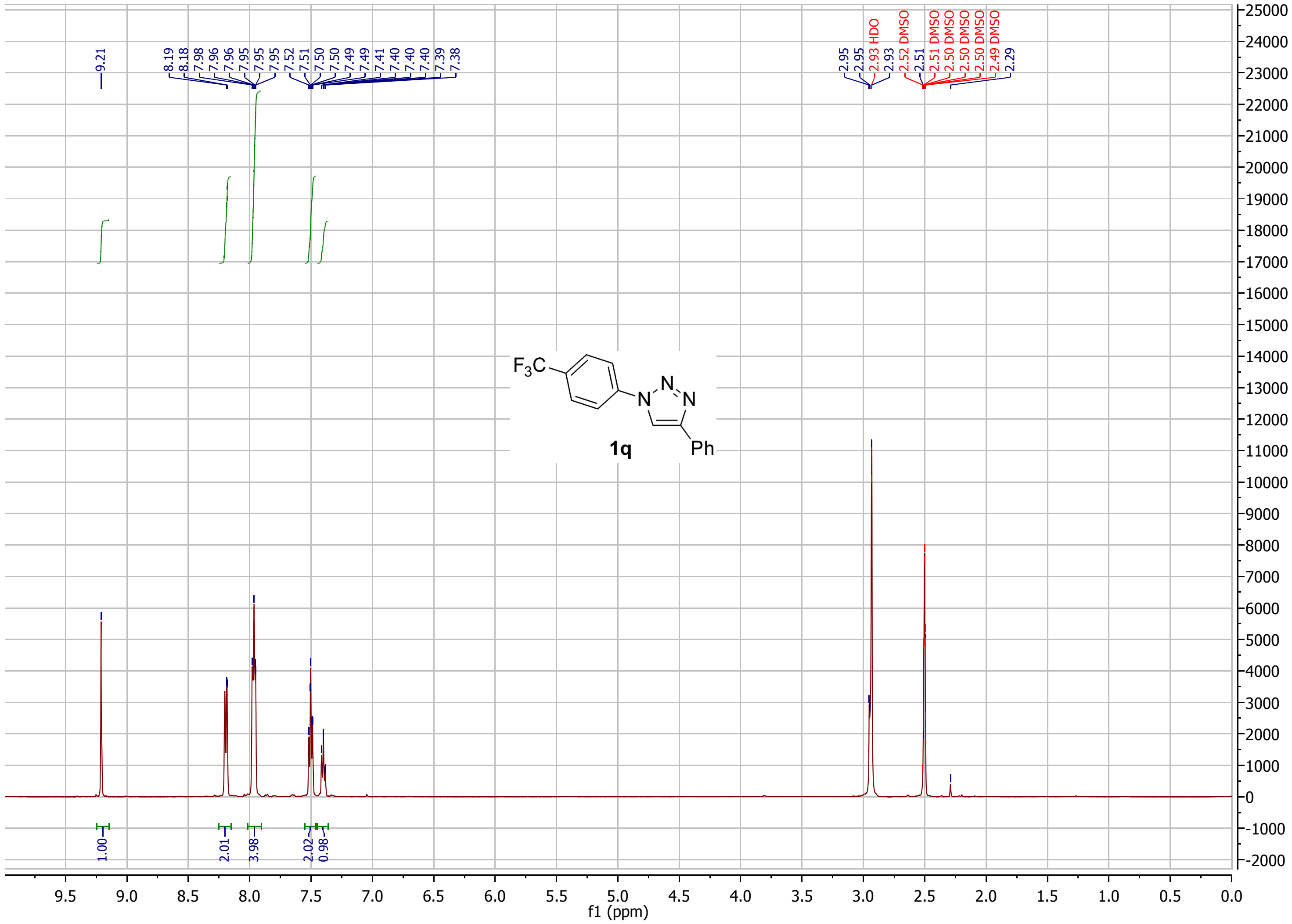
9.21
8.19
8.18
7.98
7.96
7.96
7.95
7.95
7.95
7.52
7.51
7.50
7.50
7.49
7.49
7.41
7.40
7.40
7.40
7.39
7.38
2.95
2.95
2.93 HDO
2.93
2.52 DMSO
2.51
2.51 DMSO
2.50 DMSO
2.50 DMSO
2.50 DMSO
2.49 DMSO
2.29
F3C
N
N
N
1q
Ph
1.00
2.01
3.98
2.02
0.98
f1 (ppm)

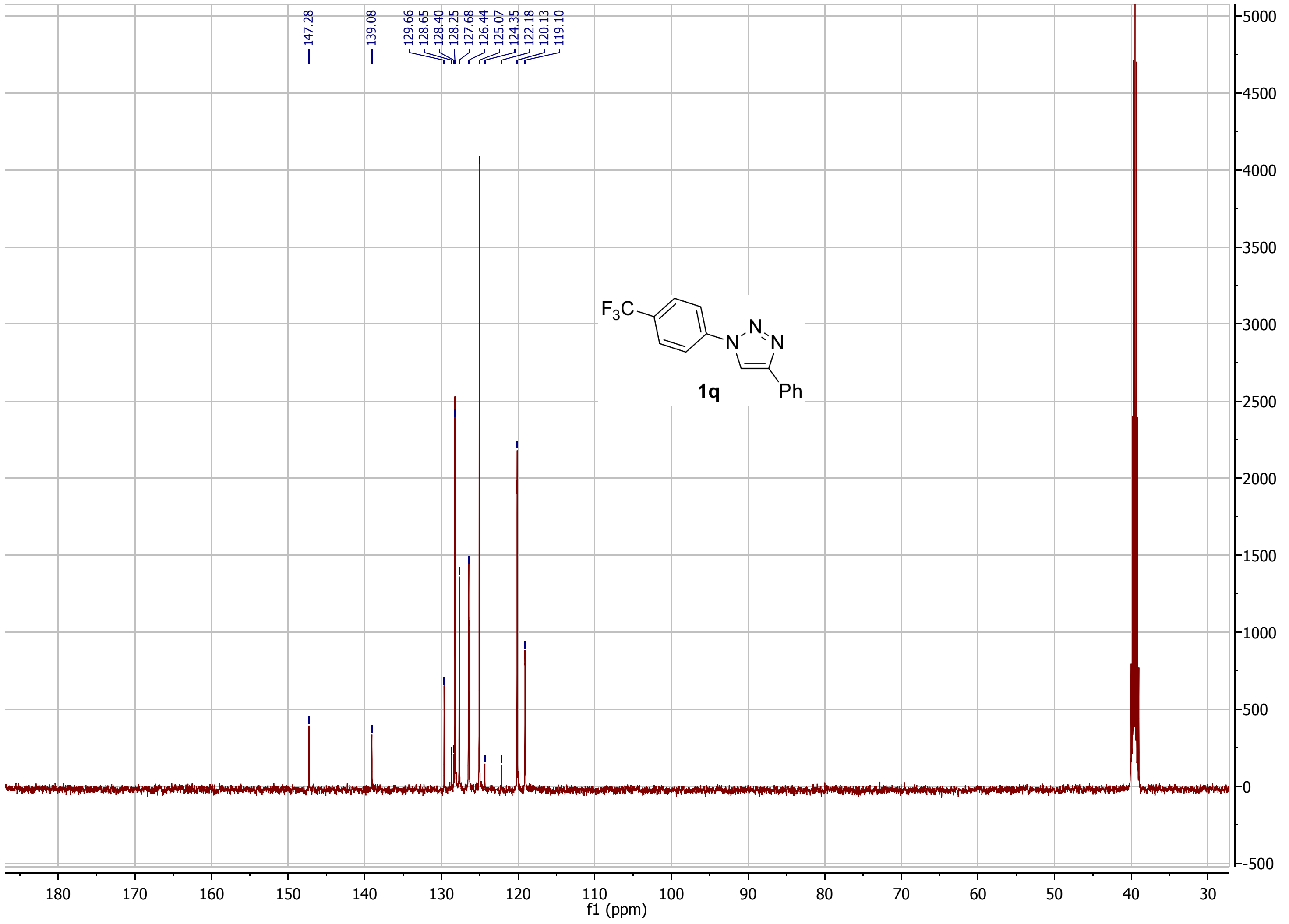
147.28
139.08
129.66
128.65
128.40
128.25
127.68
126.44
125.07
124.35
122.18
120.13
119.10
F3C
N
N
N
1q
Ph
f1 (ppm)

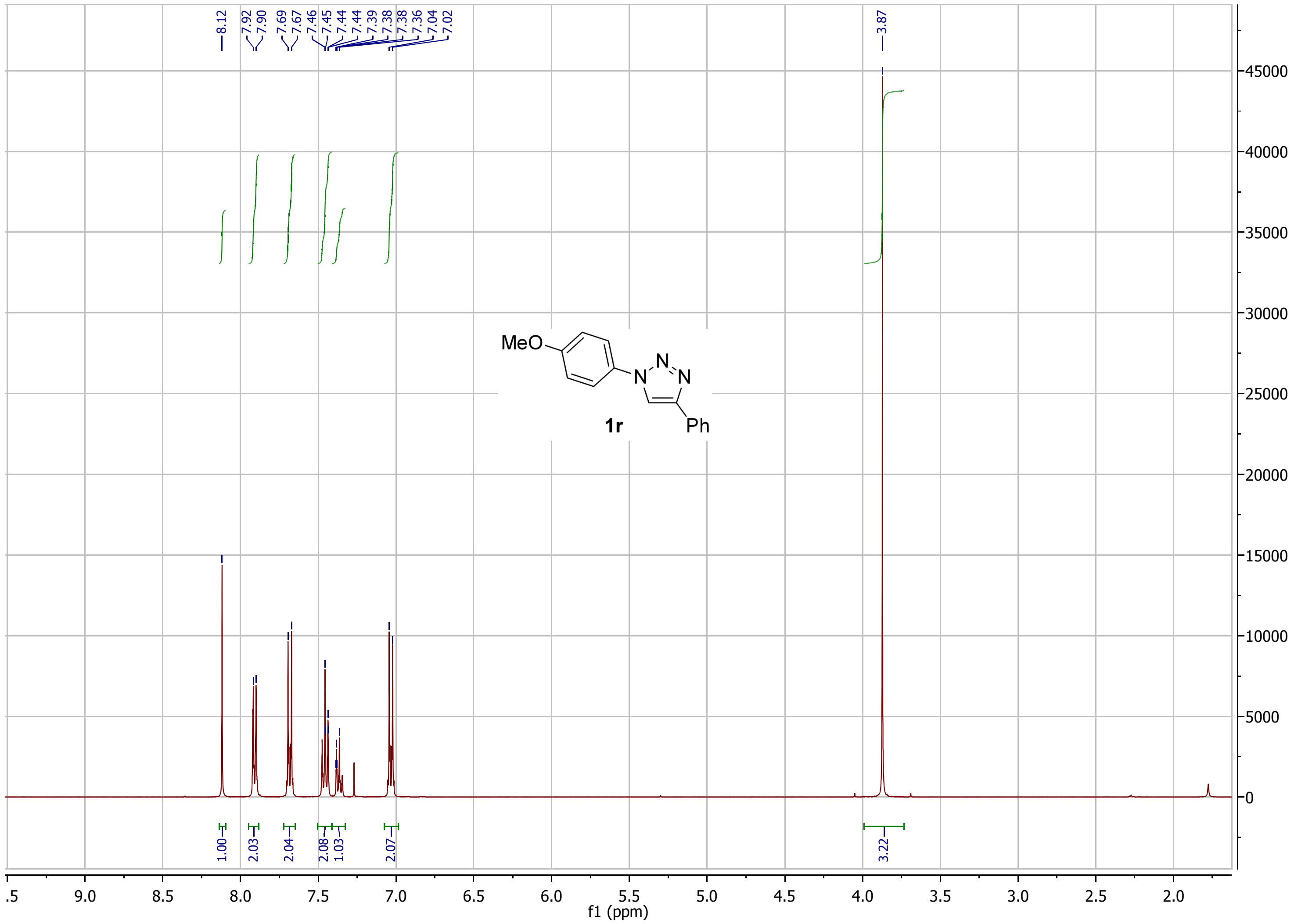

8.12
7.92
7.90
7.69
7.67
7.46
7.45
7.44
7.44
7.39
7.38
7.38
7.36
7.04
7.02
3.87
MeO
N
N
N
1r
Ph
1.00
2.03
2.04
2.08
1.03
2.07
3.22
f1 (ppm)

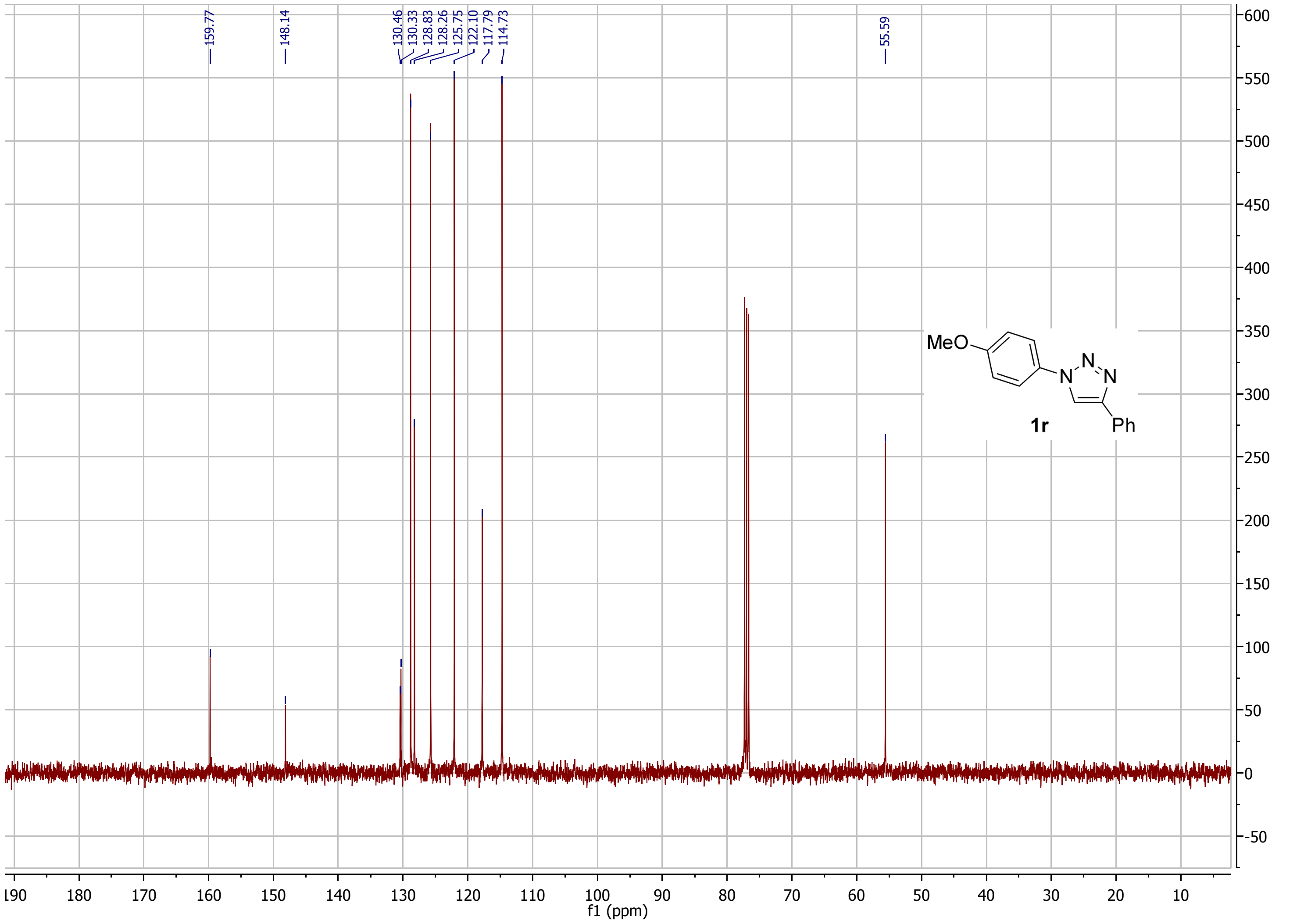

159.77
148.14
130.46
130.33
128.83
128.26
125.75
122.10
117.79
114.73
55.59
MeO
N
N
N
1r
Ph
f1 (ppm)

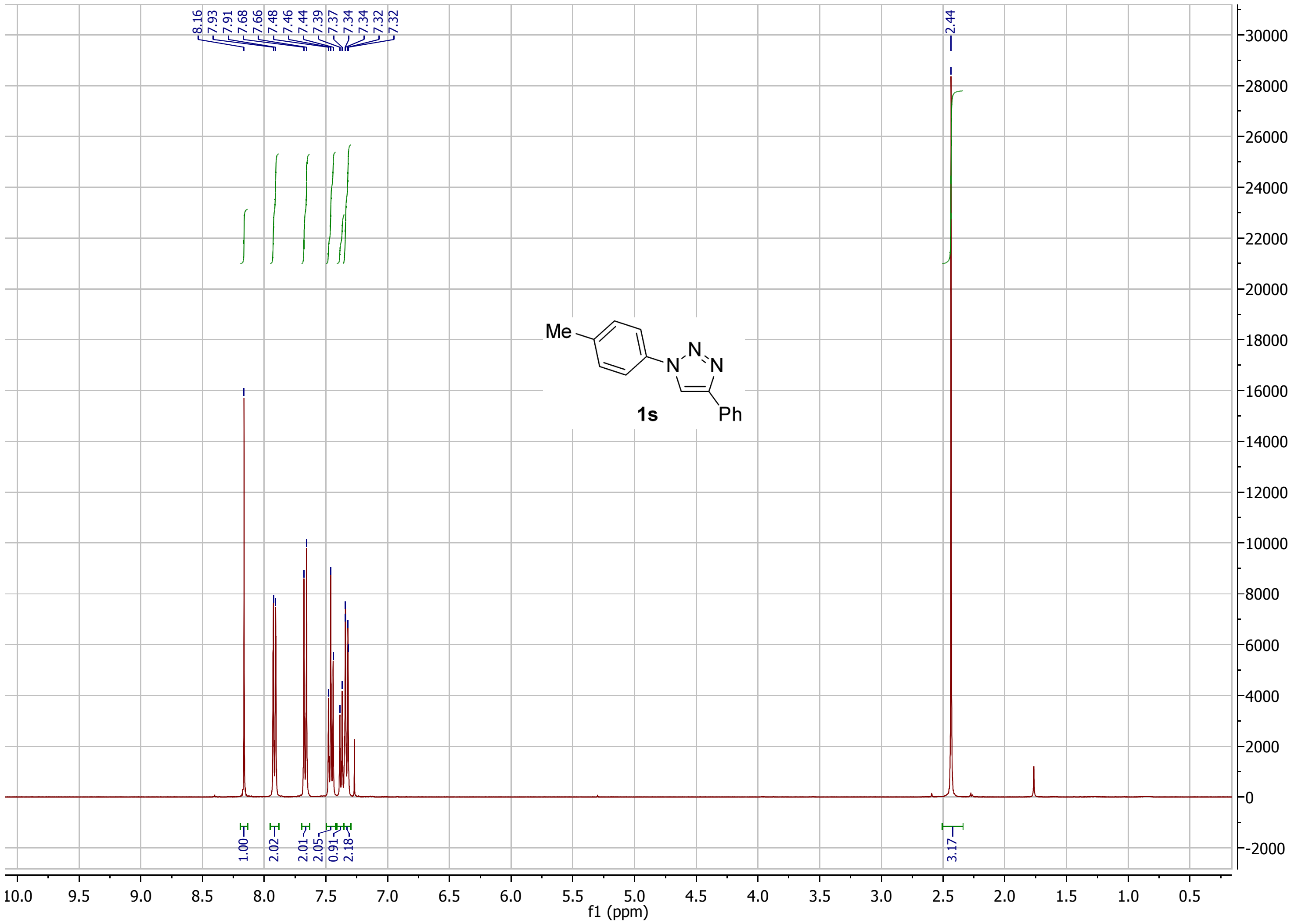
8.16
7.93
7.91
7.68
7.66
7.48
7.46
7.44
7.39
7.37
7.34
7.34
7.32
7.32
2.44
Me
N
N
N
1s
Ph
1.00
2.02
2.01
2.05
0.91
2.18
3.17
f1 (ppm)

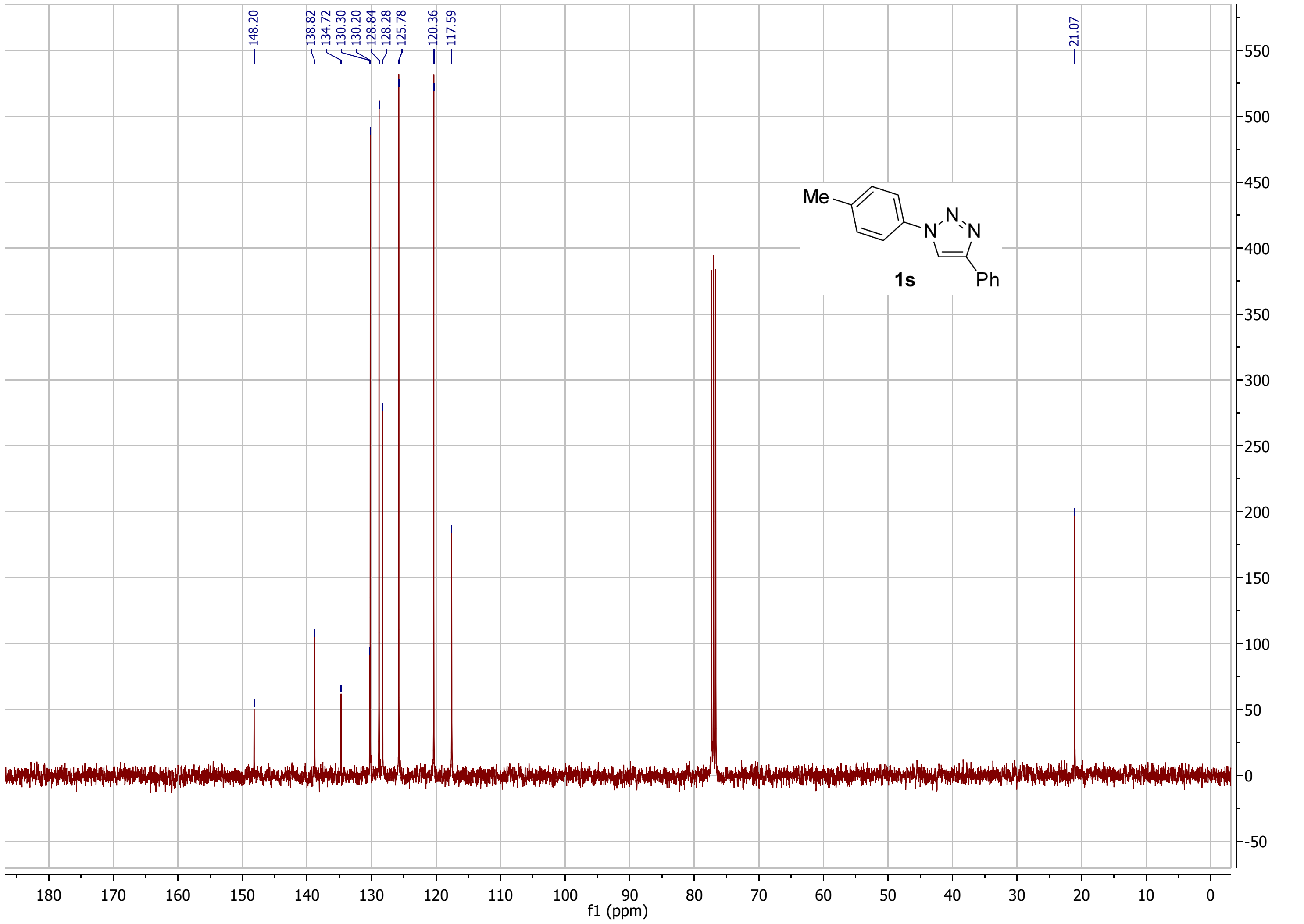
148.20
138.82
134.72
130.30
130.20
128.84
128.28
125.78
120.36
117.59
21.07
Me
N
N
N
1s
Ph
f1 (ppm)
